# Highly Efficient Approach to High-Molecular Weight Polyhydroxyurethanes

**DOI:** 10.1021/acs.macromol.5c02932

**Published:** 2026-04-24

**Authors:** Sergei V. Zubkevich, Abdurrahman Beter, Arpan Datta Sarma, Reiner Dieden, Vincent Berthé, Alexander S. Shaplov, Daniel F. Schmidt

**Affiliations:** 87145 Luxembourg Institute of Science and Technology (LIST), Functional Polymeric and Particulate Materials Unit, 5, Avenue des Hauts-Fourneaux, L-4362 Esch-sur-Alzette, Luxembourg

## Abstract

Conventional methods for synthesizing thermoplastic polyurethanes (TPUs) typically rely on isocyanates and tin-based catalysts, both of which pose significant environmental and safety risks. To mitigate these issues, alternative routes for synthesizing nonisocyanate polyurethanes (NIPUs) have been explored since the 1980s. However, most reported approaches yield polymers with molecular weights below 35 kg/mol and only a few exceptions reaching 50–70 kg/mol, often requiring oligomeric monomers or postmodification. This limitation has been a major barrier to the broader application and production of NIPUs. In this study, we demonstrate a direct polyaddition strategy for synthesizing high-molecular-weight poly­(hydroxyurethane)­s (PHUs, a subclass of NIPUs) from low-mass difunctional monomers without relying on macromonomers, oligomers, catalysts, or postcondensation/modification steps. The key to this achievement was the rational design of the monomers. The reactivity of cyclic carbonates was enhanced by incorporating aromatic rings into the structure of 7,7,7′,7′-tetramethyl-6,6′,7,7′-tetrahydro-5,5′-spirobi­[indeno­[5,6-*d*]­[1,3]­dioxole]-2,2′-dione. Model reactions of this bis­(cyclic carbonate) with secondary amines revealed that steric and electronic effects are decisive with alicyclic secondary amines markedly outperforming their linear analogues. Further pairing of activated aromatic bis­(cyclic carbonate) with alicyclic secondary diamines enabled nearly quantitative monomer conversion under mild, catalyst-free conditions (at 50 °C), yielding linear PHUs with a record-high degree of polymerization (DP_n_) of up to 220 and a number-average molecular weight (*M*_n_) of 105 kg/mol. This strategy was further extended to another aromatic cyclic carbonate, 5,5′-(9*H*-fluorene-9,9-diyl)­bis­(benzo­[*d*]­[1,3]­dioxol-2-one), producing PHUs with *M*_n_ values of up to 100 kg/mol.

## Graphical abstract

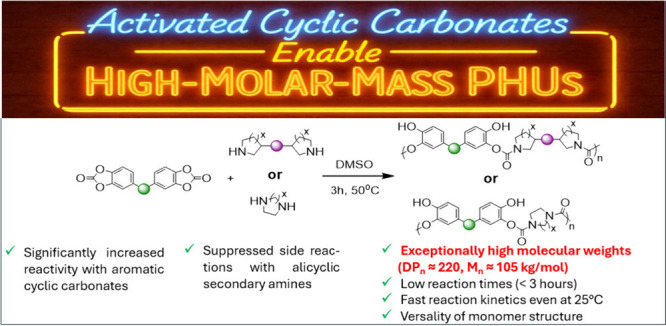

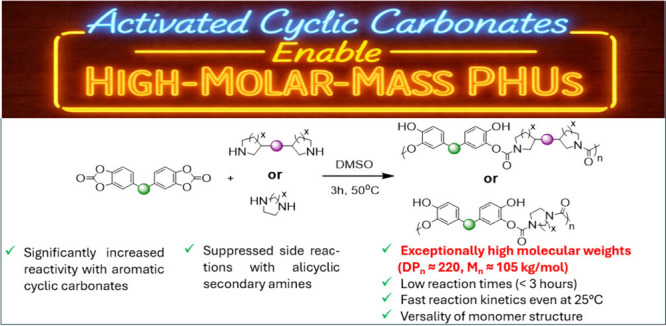

## Introduction

Polyurethanes (PUs) are a widely used and highly versatile family of plastics with global production reaching 24.7 million tons in 2021.^1^ Thanks to the vast array of available precursors, PUs are employed in a broad spectrum of applications, including foams, adhesives, automotive components, medical devices, sporting goods, coatings, and varnishes.^2^

Despite the widespread production of PUs, this industry relies heavily on toxic intermediates derived from nonrenewable fossil fuel-based resources. The conventional synthetic pathway to linear PUs involves the reaction of diols with diisocyanates, the latter representing highly toxic compounds that pose severe respiratory and dermal hazards, potentially leading to chronic illness or even death upon overexposure.^3^ Those concerns have resulted in the labeling of various isocyanates in REACH Annex XVII as carcinogenic, mutagenic, and/or reprotoxic.^4^ As a consequence, considerable research efforts in both academia and industry have focused on developing alternative synthetic routes for producing polyurethanes that avoid the use of hazardous isocyanates, the so-called nonisocyanate polyurethanes (NIPUs). In the subsequent 60+ years, however, not a single NIPU has seen widespread commercial adoption, for several reasons: (1) lack of availability of commercially produced monomers; (2) low molecular weights of the synthesized NIPUs^5–8^ (Table S1 and Figure 1); (3) poor mechanical properties (brittle films); (4) high water uptake (typically 30–35%) in the popular subclass of NIPUs known as poly­(hydroxyurethane)­s (PHUs) due to the presence of two hydroxyl groups per repeating unit, resulting in significant moisture-induced variations in properties.^9^ The inability to achieve high molecular weights in particular (and the limitations this places on both mechanical performance and rheological properties) has driven researchers to explore more efficient synthetic pathways.^5–8,10,11^ Accordingly, this review is specifically aimed at comparing the existing synthetic strategies for NIPU formation with respect to the number-average molar mass (*M*_n_) and the degree of polymerization (DP_n_). We fully acknowledge that the approaches reported in the literature (Table S1 and Figure 1) offer distinct advantages in other aspects, such as the reaction conditions, monomer scope, sustainability, or functional group tolerance. However, these considerations fall outside the defined scope of the present comparative analysis.

**Figure 1:**
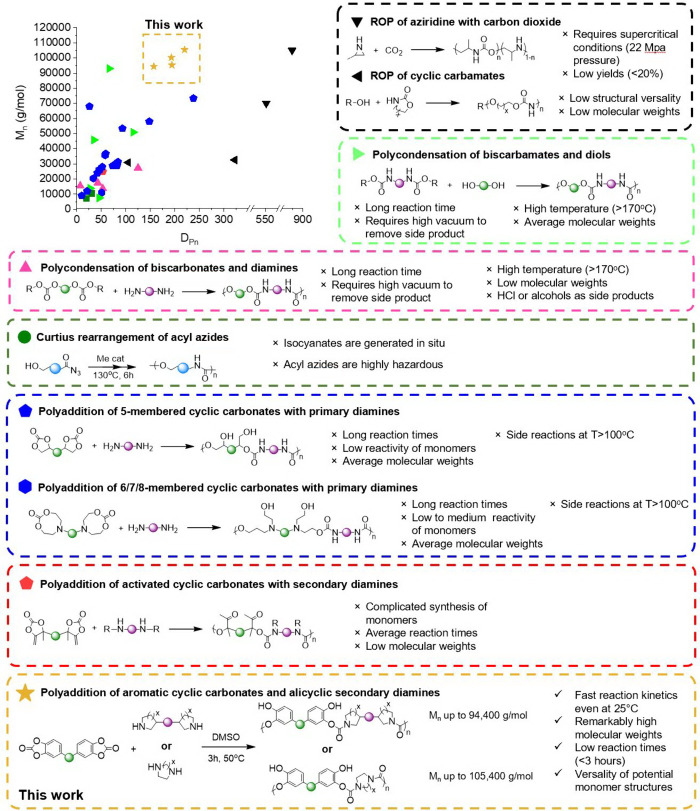
Comparison of reported maximum number-average molecular weights (*M*_n_) and degrees of polymerization (DP_n_) of nonisocyanate polyurethanes obtained by various synthetic approaches.

To date, four principal synthetic routes for linear NIPUs have been reported in the literature: polycondensation, rearrangement, ring-opening polymerization (ROP), and polyaddition (summarized in Figure 1 and Table S1). When comparing the molecular weight of NIPUs, it is important to consider not only the number-average molar mass but also the degree of polymerization. This distinction is critical, as oligomeric monomers can be incorporated into the polymerization process, effectively increasing the overall molar mass without necessarily reflecting a higher DP_n_. Thus, both parameters must be evaluated to provide an accurate picture of the efficacy of the polymerization and the properties of the resulting material.

One approach to synthesizing NIPUs involves the ROP of aliphatic cyclic carbamates^12–14^ or aziridines.^15–17^ ROP of aziridines with CO_2_ can yield high-molecular-weight polymers (*M*_n_ up to 150 kg/mol) with high DP_n_ (up to 1500) without generating any side products (Figure 1, Table S1, entry 1).^15,17^ At the same time, the methodology used to determine the molecular weights of these NIPUs raises certain concerns. This is further supported by another study in which the synthesis of the same polymers using the same monomer bearing an aziridine ring yielded only oligomeric products (Table S1, entry 39).^16^ Moreover, this reaction typically requires elevated temperatures (>170 °C) and pressures (22 MPa), which limits its practicality.^15^ Adding to this, the synthesis of cyclic carbamates often relies on phosgene-based processes, and the inherent toxicity of aziridines presents a significant safety concern. ROP of cyclic carbamates typically yields *M*_n_ within 7.5–33 kg/mol (Table S1, entries 15, 37) with DP_n_ ranging from 46 to 322.^6^ Additionally, structural diversity is limited, as only a few commercially available monomers exist: two substituted aziridines (methyl and phenyl) and two or three aliphatic cyclic carbamates (1,3-oxazinan-2-one, 6-acetylbenzo­[*d*]­oxazol-2­(3*H*)-one, etc.).

Within the polycondensation category, several approaches are reported, including reactions between activated biscarbonates and diamines^18–24^ (Figure 1 and Table S1, entries 20, 27, 28, 30, 35) or activated biscarbamates and polyols^25–31^ (Table S1, entries 6, 11, 12, 17, 24, 29, 36) via transurethanization (Figure 1). However, these methods typically require the use of phosgene or its derivatives for precursor synthesis. Additionally, polycondensation reactions often generate side products such as HCl or alcohols, which pose significant challenges for industrial scalability and environmental compliance. In the case of transurethanization between biscarbonates and diamines (Table S1, entries 20, 27, 28, 30, 35), the resulting NIPUs typically exhibit *M*_n_ values in the range of 13–18 kg/mol, with rare exceptions reaching 24 ^24^ and 27 kg/mol,^23^ respectively. The corresponding DP_n_ generally falls between 40 and 66 repeating units. In contrast, transurethanization between activated biscarbamates and polyols enables the attainment of significantly higher molecular weights, with *M*_n_ values ranging from 40 to 50 kg/mol^26,27^ (Table S1, entries 11, 12, 17), and an absolute record of 93 kg/mol accompanied by a DP_n_ of 66 ^29^ (Table S1, line 6).

The synthesis of NIPUs can be performed via Curtius rearrangement of acyl azides (Figure 1 and Table S1, entries 33, 38).^32–36^ These transformations generate isocyanates in situ, which subsequently react with alcohols to form PUs. Although the isocyanates are not introduced directly, their formation is inherent to the process. More critically, the starting materials, namely, acyl azides and hydroxamic acids, are highly hazardous and pose significant safety concerns. Additionally, the highest reported *M*_n_ reached only 10 kg/mol^6,33^ with a DP_n_ of 31 via this approach (Table S1, entry 33).

The final synthetic route for producing NIPUs involves polyaddition reactions between cyclic carbonates and amines (Figure 1 and Table S1, entries 7–10, 13–14, 16, 18–19, 21–22, 25–26, 31–32, 34).^37–48^ This approach is considered the most promising for NIPU synthesis,^5–8,49^ as it completely eliminates the use of isocyanates and phosgene. In addition, cyclic carbonates are generally nontoxic, nonvolatile, and significantly less sensitive to moisture than isocyanates. In contrast, isocyanates readily react with water to generate unstable carbamic acids that decompose into amines and CO_2_; the resulting amines can further react with residual isocyanate groups to form urea linkages, thereby leading to undesirable side reactions. Thus, cyclic carbonates offer safer and more convenient handling and storage without the need for special precautions. Moreover, the polyaddition process does not generate volatile organic compounds (VOCs), making it highly suitable for environmentally friendly coating applications.^6–8,49^ The resulting polymers are termed PHUs due to the presence of both primary and secondary hydroxyl groups along the polymer backbone. The synthesis of linear PHUs (Figure 1) via the reaction of cyclic carbonates and amines (aminolysis) was suggested as an alternative to PU formation as far back as 1957.^50^ For PHUs synthesized from small-molecule monomers, the vast majority of publications report average molecular weights not exceeding 15–20 kg/mol,^6^ with DP_n_ values ranging from 10 to 80 (Table S1). Two studies described PHUs with *M*_n_ values reaching 36 kg/mol^42,43^ (Table S1, entries 13–14), and one demonstrated an Mn value of 53 kg/mol and a DP_n_ of 93 ^41^ (Table S1, entry 10). This value can be increased up to 70 kg/mol^39^ (Table S1, entries 7–8) by employing oligomeric monomers, although this often results in a significant decrease in the degree of polymerization.

Several strategies have been explored to increase the molecular weight of linear PHUs via polyaddition: (1) enhancing the reactivity of bis­(cyclic carbonate)­s (BCCs) by replacing 5-membered cycles with more reactive 6-, 7-, or 8-membered analogs;^38,43,46^ (2) increasing monomer concentration by shifting from solution polyaddition to bulk reactive extrusion;^37,51^ and (3) introducing catalysts and hydrogen-bond disruptors.^51–53^ Despite these efforts, the inherent reactivity of BCCs, and consequently the polymerization kinetics, remained low. Combined with the occurrence of side reactions above 100 °C,^54–57^ these factors limited the *M*_n_ of the resulting PHUs. Recently, the group of Jérôme and Detrembleur reported the activation of BCC monomers through the introduction of an exocyclic double bond as an activating substituent.^58^ Two bis-α-alkylidene cyclic carbonate monomers were designed: one bearing internal exocyclic olefins and the other, external ones. The exceptional reactivity of these BCCs toward primary and secondary amines arises from the exocyclic olefinic group, which directs the selective ring-opening of the cyclic carbonate via enol formation. This enhanced reactivity allowed the aminolysis temperature to be reduced from 100 to 25 °C; however, the resulting PHUs reached only moderate molecular weights, with *M*_n_ limited to 25 kg/mol and a DP_n_ of 53 ^58^ (Figure 1 and Table S1, entry 23).

In a recent study, Tabanelli and co-workers investigated the reactivity of catechol carbonate with various amines.^59^ They found that both primary and secondary amines react rapidly with catechol carbonate at 25 °C in the absence of catalysts, achieving full conversion within seconds.^59^ Furthermore, while primary amines induced a second attack on the resulting carbonate moiety, leading to urea formation accompanied by catechol elimination (Figure 2A), the secondary amines afforded stable 2-hydroxyphenylcarbamates (Figure 2B).

**Figure 2:**
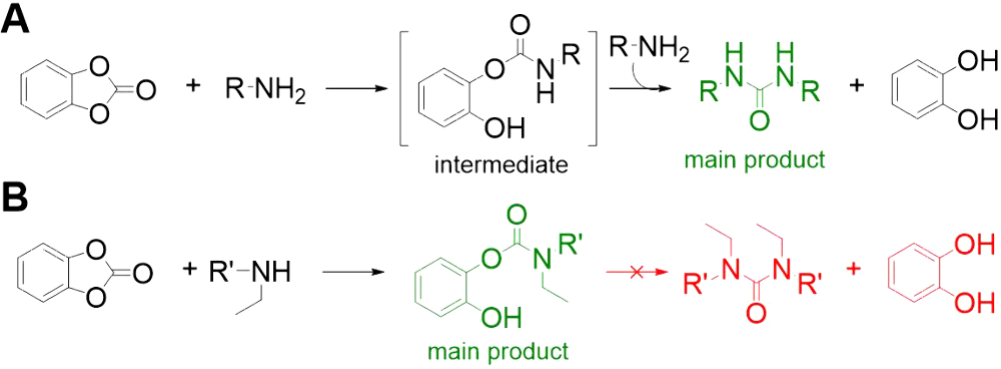
Reactions of catechol carbonate with primary and secondary amines.^59^

Inspired by the work of Tabanelli,^59^ we adopted a similar strategy to activate BCC monomers by directly fusing cyclic carbonates to the aromatic rings (Figure 3A) and then, for the first time,^60^ examined their reactivity in polyaddition with various secondary amines, with the aim of obtaining high-molecular-weight thermoplastic PHUs (Figure 3C,D). We would like to emphasize that the approach proposed in the present work is fundamentally distinct from that reported by Jérôme and Detrembleur.^58^ In our case, the unsaturated functionality within the cyclic carbonate moiety is embedded in an aromatic ring system (Figure 3A), which renders it electronically and chemically different from the systems previously described. Consequently, their reactivity profile and activation mode are not directly comparable.

**Figure 3:**
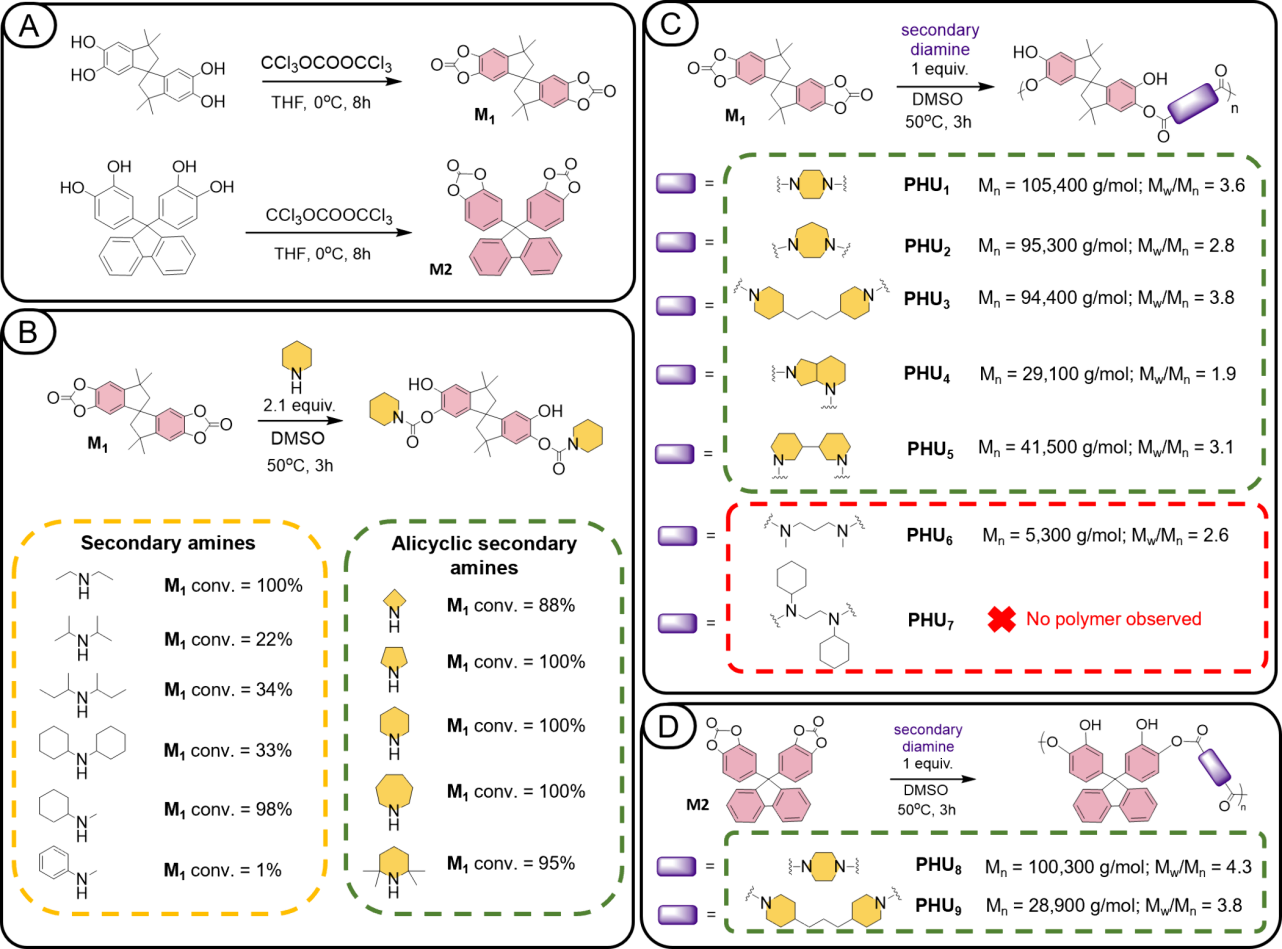
(A) Synthetic route to aromatic cyclic carbonate monomers **M**_**1**_ and **M**_**2**_; (B) simplified scheme for the preparation of model compounds from **M**_**1**_ with linear and alicyclic secondary amines; (C) simplified scheme for the synthesis of **PHU**_**1**_-**PHU**_**7**_ polymers from **M**_**1**_; (D) synthetic route to **PHU**_**8**_ and **PHU**_**9**_ polymers from **M**_**2**_.

## Results and Discussion

### Synthesis of Activated Bis­(Cyclic Carbonate)­s

First, two aromatic BCC monomers, **M**_**1**_ and **M**_**2**_ (Figure 3A), were synthesized from commercially available molecules containing two ortho-phenol moieties by reaction with triphosgene in THF at 0 °C. The reaction proceeded rapidly and afforded good yields (68–88%) after recrystallization. As the primary objective of this study was not the optimization of monomer synthesis from a sustainability perspective, triphosgene was employed for the reasons of synthetic convenience and reproducibility. Nevertheless, **M**_**1**_ and **M**_**2**_ cyclic carbonate monomers can also be prepared via more sustainable routes, for example, through transcarbonation reactions using dimethyl carbonate (DMC)^61,62^ or diphenyl carbonate (DPC). However, as the precursors used in this study were insoluble in DMC, achieving comparable yields and purity by such methods would therefore require further optimization that was beyond the scope of the current report. Monomers **M**_**1**_ and **M**_**2**_ were isolated as white crystals, and their structures and purity levels were confirmed by NMR (Figures S2–S3 and S6–S7) and IR spectroscopy (Figures S4 and S8) as well as by elemental analysis. A comprehensive attribution of the NMR spectra is presented in the Supporting Information (Figures S2–S3 and S6–S7). FTIR spectroscopy was carried out to complement the NMR analysis. Here, the conversion of catechol rings into cyclic carbonates was evidenced by the disappearance of the broad OH stretching bands at 3200–3500 cm^–1^ and the phenolic C–O stretching bands at 1279–1299 cm^–1^. Concurrently, new absorptions appeared at 1804, ∼1830, and ∼1860 cm^–1^, corresponding to the C=O stretching of carbonate groups, as well as at 764 and 927–956 cm^–1^, attributable to the asymmetric and symmetric C–O–C stretching vibrations of the carbonate moiety (Figures S4 and S8). The sharp melting peaks observed in the DSC traces (Figures S1 and S5) further supported the high purity of these monomers with **M**_**1**_ and **M**_**2**_ exhibiting melting points of 269.3 and 240.0 °C, respectively. Notably, DSC analysis of **M**_**1**_ was carried out using a crucible sealed in an argon-filled glovebox as its melting point exceeded the onset of degradation in air.

### Model Reactions

In the next step, model reactions between **M**_**1**_ and various monofunctional secondary amines were systematically investigated (Figures 3B, S7, and S8). The reactions were carried out directly in DMSO-*d*_6_ at a concentration of 0.2 mol/L and 50 °C. To compensate for the volatility of some monofunctional amines, a slight excess of amine was used (amine:BCC = 2.1:1.0, molar ratio). Reaction conversions were determined by ^1^H NMR based on the ratio of the signals at 6.03–6.05 ppm (two aromatic protons of reacted **M**_**1**_) to those at 7.40–7.46 ppm (two aromatic protons of unreacted **M**_**1**_).

In the case of unhindered aliphatic amines, such as diethylamine, full conversion was achieved within minutes (Figure S7a). Increasing steric hindrance, as in diisopropylamine and diisobutylamine, reduced conversions after 5 h to 80% and 44%, respectively (Figures S9b–c). The strong steric effect was further confirmed with dicyclohexylamine, which afforded only 36% conversion (Figure S9d). Further confirming the importance of sterics, replacing one of the cyclohexyl substituents with a methyl, as in *N*-methylcyclohexanamine, once more provided quantitative conversion (Figure S9e). In contrast, lowering the electron density on the amine nitrogen, as observed when switching from *N*-methylcyclohexanamine to *N*-methylaniline, almost entirely suppressed the reaction (Figures S9e,d). Interestingly, reactions of **M**_**1**_ with cyclic aliphatic amines showed that, apart from the thermodynamically stabilized four-membered azetidine, the five-, six-, and seven-membered cyclic amines all reacted quantitatively within minutes at 50 °C (Figure S10). It can be concluded that nearly all of the selected alicyclic amines outperformed most of the linear secondary amines, likely due to their structural features and higher basicity. Overall, these results suggest that the most promising candidates for rapid polyaddition with **M**_**1**_ are cyclic aliphatic amines containing more than four carbon atoms in the ring. Finally, before proceeding to polyaddition, it was essential to elucidate the structure of the products formed in the model reactions. For this purpose, the product of the reaction between **M**_**1**_ and one of the most promising cyclic amines, the six-membered piperidine, was selected for NMR analysis (Figure S11). According to ^1^H NMR, the reaction afforded the desired disubstituted urethane, formed as a mixture of three isomers: the two symmetrical isomers (A and C) and the one bearing a stereocenter (B).

Although the signals corresponding to isomer B can be readily identified due to the 4 differentiated signals of the same intensity, all aromatic protons of isomers A, B, and C could be unambiguously assigned using a combination of 2D NMR techniques (for details, see Figures S11–S14). The molar ratio of A/B/C was determined to be 0.65:1:0.38 (Figure S11).

### Polyaddition

Based on the results of the model reaction studies, piperazine was selected as the diamine partner for the **M**_**1**_ monomer in the synthesis trial of **PHU**_**1**_ (Figure 3C). We first optimized the reaction conditions by varying the solvent, reaction time, and monomer concentration (Figure 4). A detailed analysis of all experiments (Figure 4) revealed that the optimal conditions for the synthesis of **PHU**_**1**_ with the highest molecular weight and yield are as follows: a combined monomer concentration of 3.00 mol/L, reaction temperature of 50 °C, reaction time of 3 h, and DMSO as the solvent. The choice of reaction temperature warrants further clarification. Although the model reactions between catechol carbonate and secondary amines reported by Tabanelli et al.^59^ clearly demonstrate that aminolysis of activated aromatic cyclic carbonates can proceed efficiently at room temperature, all polyaddition experiments in the present study were performed at 50 °C. While room-temperature reactivity is feasible in principle, the formation of high-molecular-weight PHUs under such conditions was strongly constrained by the rapid increase in solution viscosity. A moderate elevation of the temperature was therefore required to preserve processability and to reach higher degrees of polymerization. Importantly, this observation underscores a key advantage of the strategy proposed herein: the intrinsic reaction kinetics is sufficiently fast that it no longer constitutes the rate-limiting factor in defining the processing window. Instead, simple engineering considerations, primarily the ability to efficiently stir the increasingly viscous reaction mixture, govern the optimal temperature. Under these conditions, 50 °C represents the lowest practical temperature that ensures convenient polymerization without the need for further heating as typically required for less reactive cyclic carbonates..^5–8^ Although **PHU**_**1**_ synthesized in DMF exhibited a higher molecular weight than that obtained in DMSO (Figure 4A), the latter was chosen for further reactions, as it represents a “greener” alternative. The structure of synthesized **PHU**_**1**_ samples was further confirmed by ^1^H and ^13^C NMR (Figures S15–S16).

**Figure 4:**
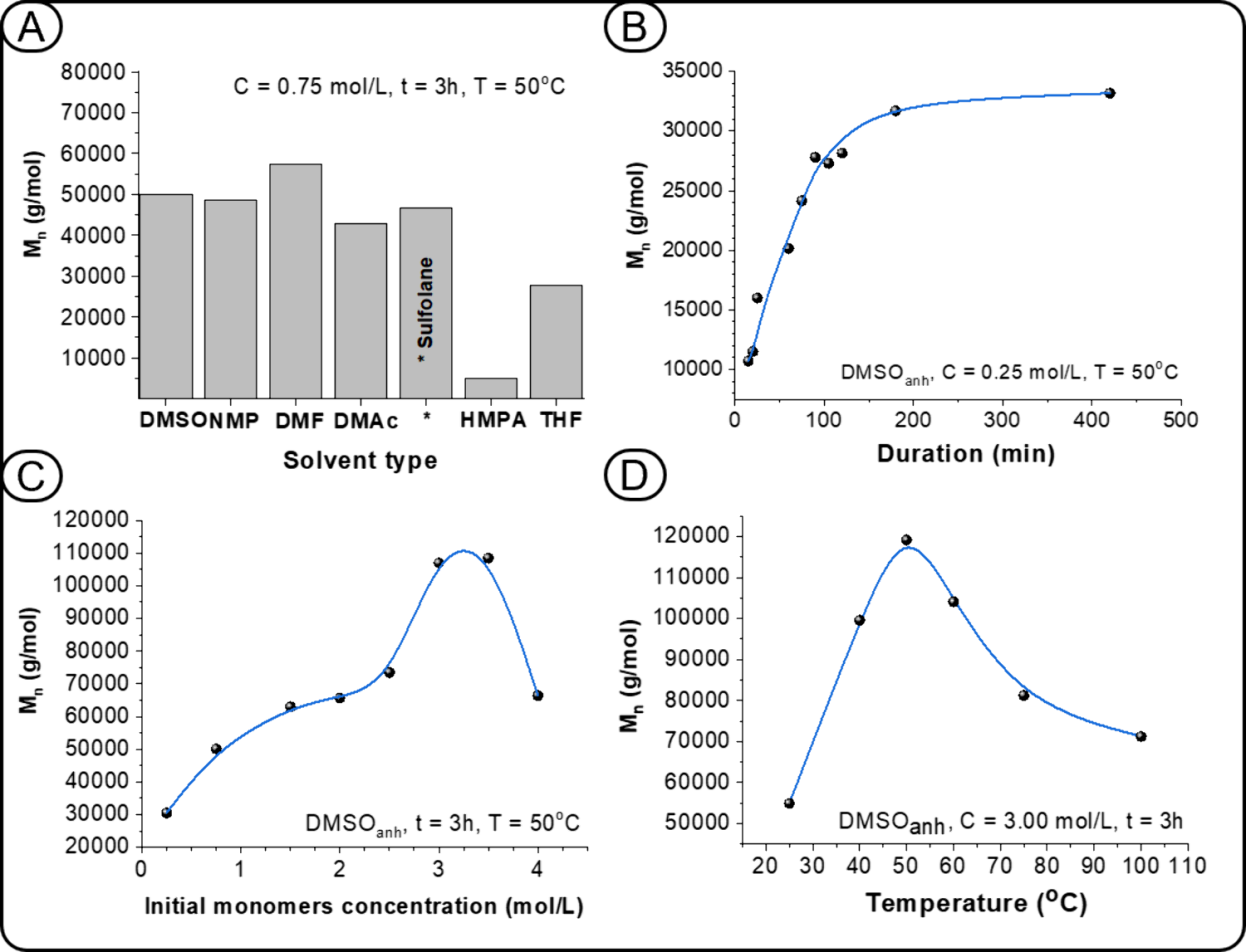
Optimization of the reaction parameters for **PHU**_**1**_ synthesis: influence of (A) solvent type, (B) reaction time, (C) monomer concentration, and (D) temperature.

The number-average molecular weights of the **PHU**_**1**_ samples were determined by GPC and reached up to *M*_n_ = 119 kg/mol (Figures 4 and S36, Table 1). Since PHUs contain pendant hydroxyl groups, they may exhibit the so-called *polyelectrolyte effect*, which can lead to an apparent overestimation of *M*_n_ values. To verify the absence of this effect, intrinsic viscosities were measured in neat DMF (Figure S37) and in DMF containing a low-molecular-weight salt, Li­(CF_3_SO_2_N)_2_. In both cases, no polyelectrolyte effect was observed, confirming the appropriate choice of solvent. Although the *M*_n_ values obtained by GPC may still be somewhat uncertain due to calibration with PMMA standards, the measured intrinsic viscosity of 0.68 dL/g supports the formation of high-molecular-weight **PHU**_**1**_. To further eliminate the influence of calibration standards, the absolute molecular weight (*M*_sD_) was determined for the same **PHU**_**1**_ sample by sedimentation–diffusion analysis in DMF (Table S3). While the obtained value (*M*_sD_ = 71 kg/mol) was somewhat lower than that determined by GPC (*M*_n_ = 105 kg/mol), it nonetheless represents a record-high molecular weight for PHUs synthesized by the polyaddition of low-molecular-weight monomers (Figure 1). The determined *M*_n_ corresponds to a degree of polymerization (DP_n_) of approximately 220 monomer units, ranking the obtained **PHU**_**1**_ as the second highest molecular weight linear NIPU reported in the literature (Table S1). It can be assumed that the high molecular weights of PHUs result from the combined influence of several factors, specifically, the enhanced reactivity of aromatic cyclic carbonates^59^ and the much more limited reaction pathways open to secondary alicyclic amines^63,64^ due to increased steric hindrance. Together, these effects significantly promote the main polyaddition reaction while effectively suppressing the side reactions.

**Table 1:** Selected Properties of the Synthesized PHUs

Sample^a^	Yield (%)^b^	*M*_n_ (g/mol)^c^	*M*_w_/*M*_n_ ^c^	DP_n_ ^d^	*T*_g_ (°C)^e^	*T*_g_ (°C)^f^	*T*_onset_ (°C)^g^
**PHU**_**1**_	95	105 400^h^	3.6	220	n.d.	253	225
**PHU**_**2**_	87	95 300	2.8	194	231	263	205
**PHU**_**3**_	80	94 400^i^	3.8	157	193	226	260
**PHU**_**4**_	82	29 100	1.9	56	258	262	235
**PHU**_**5**_	65	41 500	3.1	74	216	242	240
**PHU**_**6**_	80	5300	2.6	11	103	125	180
**PHU**_**8**_	96	100 300	4.3	193	215	259	205
**PHU**_**9**_	93	28 900	3.8	45	194	207	265

a PHU_7_ was obtained as a low-molecular weight pasty mass and was not investigated further.

b Isolated yield of polymers.

c Determined by GPC in 0.1 M Li­(CF_3_SO_2_)_2_N solution in DMF at 50 °C with PMMA standards.

d Degree of polymerization.

e Determined by DSC with a heating rate of 5 °C/min.

f Determined by TMA in He with a heating rate of 5 °C/min.

g Determined by TGA in air with a heating rate of 5 °C/min.

h *M*_sD_ = 71 200 g/mol (by sedimentation analysis in DMF), [η]_DMF_ = 0.68 dL/g.

i *M*_sD_ = 82 500 g/mol (by sedimentation analysis in DMF), [η]_DMF_ = 0.90 dL/g.

The synthesis of PHUs was further extended to four additional alicyclic secondary diamines (Figure 3C): 1,4-diazacycloheptane (**PHU**_**2**_), 1,3-di­(piperidin-4-yl)­propane (**PHU**_**3**_), octahydro-1*H*-pyrrolo­[3,4-*b*]­pyridine (**PHU**_**4**_), and 3,3′-bipiperidine (**PHU**_**5**_). The structure of all synthesized **PHU** samples was confirmed by ^1^H and ^13^C NMR (Figures S17–S25 and S27–S30). All of the selected diamines yielded PHUs with relatively high molecular weights, according to GPC analysis (Table 1).

The *M*_n_ values of **PHU**_**2**_ and **PHU**_**3**_ were comparable to those obtained with piperazine (**PHU**_**1**_), reflecting the structural similarity of the corresponding diamines. To confirm the molecular weight of **PHU**_**3**_, its characterization was repeated using two independent methods: viscometry and sedimentation–diffusion analysis (Table 1). The intrinsic viscosity of 0.90 dL/g (Figure S38), together with the calculated *M*_sD_ value of 83 kg/mol, provides unequivocal evidence of the high molecular weight of **PHU**_**3**_. In contrast, conformational constraints introduced by the rigid structures in **PHU**_**4**_ and **PHU**_**5**_ are believed to have led to their reduced molecular weights of 29 and 42 kg/mol, respectively (Table 1).

The *M*_w_/*M*_n_ ratios of the synthesized high-molecular-weight PHUs ranged from 1.9 to 4.3, depending on the monomer structure employed (Table 1 and Figure S3). In several cases, the dispersity exceeded the value of 2.0 predicted by Flory’s theory for ideal step-growth polymerization. It should be emphasized, however, that Flory’s prediction relies on two fundamental assumptions: (i) equal reactivity of functional groups and (ii) molecular weight-independent reactivity throughout the course of polymerization. In the present system, deviation from the theoretical value can be rationalized primarily in relation to the second assumption. The polymerization is conducted in a polar aprotic solvent (DMSO) starting from a bis­(cyclic carbonate) monomer and a secondary alicyclic diamine. As the reaction proceeds and PHU chains are formed, the concentration of hydroxyl groups in the reaction medium increases substantially. This progressive buildup of hydroxyl functionalities is expected to markedly influence the solvation environment, dielectric properties of the medium, and the extent of hydrogen bonding between monomers and growing chains.^51–53^ Such evolving reaction conditions inevitably alter the effective reactivity of functional groups as a function of the chain length. Consequently, the assumption of constant, molecular weight-independent reactivity is no longer strictly valid. This provides a sound mechanistic basis for the observed deviation from Flory’s predicted dispersity of 2, even in the absence of significant side reactions.

The GPC traces of most PHUs synthesized in this study exhibit a bimodal molecular weight distribution (Figure S36). A first plausible explanation for this behavior is the formation of macrocyclic species, as discussed by Kricheldorf in the context of ring–chain and ring–ring equilibria in step-growth polymerizations.^65–67^ The presence of cyclic oligomers alongside linear chains naturally leads to bimodality and may also account for the deviations in dispersity observed above. Notably, **PHU**_**4**_ and **PHU**_**6**_, which do not display bimodality in the high-molecular-weight region (Figure S36), represent special cases. **PHU**_**4**_, although of high molecular weight, is derived from a highly rigid and asymmetric diamine. Such structural features are expected to strongly disfavor intramolecular cyclization, thereby suppressing the macrocycle formation. As a consequence, a monomodal molecular weight distribution is observed with a dispersity of 1.9, which is very close to the value predicted by Flory’s theory for ideal step-growth polymerization. In contrast, **PHU**_**6**_ is based on a flexible diamine and attains only low molecular weight. In this case, macrocycle formation is entropically disfavored, as effective intramolecular cyclization competes poorly with intermolecular growth at low chain lengths. This results in a monomodal distribution and a low dispersity (*M*_w_/*M*_n_ = 2.6), likely reflecting the increased contribution of side reactions in this system due to the acyclic nature of the amine component (see *vide infra*).

The second explanation of the observed bimodal molecular weight distribution (Figure S36) can be connected with the partial hydrolysis of the cyclic carbonate monomer **M**_**1**_ during the polyaddition reaction. Indeed, in a recent report on catechol carbonate chemistry,^61^ it was shown that this highly activated carbonate is prone to hydrolysis at elevated temperatures, even under rigorously anhydrous conditions. In that study, carbonate formation at 80 °C was strongly hindered by hydrolysis despite inert conditions; lowering the temperature to 60 °C and using excess alcohol improved, but did not eliminate, this effect.^61^ To confirm similar behavior, the cyclic carbonate monomer **M**_**1**_ was exposed in DMSO to trace amounts of water at 50 °C for 12 h (Figure S55) with the ^1^H NMR spectra providing clear evidence of hydrolysis. Given this moisture sensitivity, we propose that trace water, difficult to fully exclude due to the hygroscopic nature of DMSO and amines, was present in all reaction mixtures. At early stages, high concentrations of amine and activated carbonate ensure rapid polymerization to high molar mass. As the reaction proceeds, their concentrations decrease while water amount remains constant. At a critical point, water begins to compete for the remaining activated carbonate groups, partially deactivating them. Although insufficient to halt polymerization entirely, this process limits growth for a fraction of chains, while others continue to higher molar mass. If this hypothesis is correct, GPC traces should be unimodal at early reaction times, when hydrolysis is negligible and the chain statistics approximate Flory’s theory. A second mode would then emerge at later stages, as chains that continue to grow become distinguishable from those whose growth has been terminated by cyclic carbonate hydrolysis. Consistent with this hypothesis, analysis of time-resolved SEC data (Figure 4B) reveals the expected trend: a single peak at early stages (Figure S56, 10–60 min), followed by the emergence of a second peak in the high molar mass region after 90 min (Figure S56, 90 min).

Switching to linear secondary diamines, namely, to *N* ^1^,*N* ^3^-dimethylpropane-1,3-diamine and *N*,*N*′-dicyclohexyl-1,2-ethanediamine, led either to the formation of only low-molecular-weight **PHU**_**6**_ (Table 1) or, in the case of **PHU**_**7**_, to a mixture of low-molecular-weight species rather than a well-defined polymer. A detailed analysis of the ^1^H NMR spectrum of the products obtained from the **PHU**_**7**_ synthesis (Figure S26), together with the overlaid spectra of **PHU**_**6**_ and **PHU**_**7**_ (Figures S33–S35), allows the following conclusions to be drawn: (1) For the precipitated products of **PHU**_**7**_, the formation of cyclic urea (1,3-dicyclohexylimidazolidin-2-one) was unambiguously confirmed by the assignment of characteristic resonances in the 3.6–1.0 ppm region^68^ (Figures S26 and S35). (2) In the case of **PHU**_**6**_, discrete cyclic urea species could not be directly detected in the isolated oligomer; most likely, low-molecular-weight cyclic ureas were removed during the precipitation step (Figure S33). (3) For both **PHU**_**6**_ and **PHU**_**7**_, signals corresponding to unreacted cyclic carbonate end groups were identified (Figures S33 and S34). (4) For both systems, characteristic OH resonances attributable to ortho-diphenol (RR′C_6_H_2_(OH)_2_) moieties were clearly observed. Overall, in both cases, the majority of cyclic carbonate units appears to have reverted to the original ortho-diphenol structure, supporting the occurrence of carbonyl abstraction via secondary amine attack accompanied by stable cyclic urea (1,3-disubstituted imidazolidin-2-one) elimination (Figure 5). Such side reactions are not feasible in the case of alicyclic secondary diamines due to the steric strain imposed near the urethane bond.

**Figure 5:**
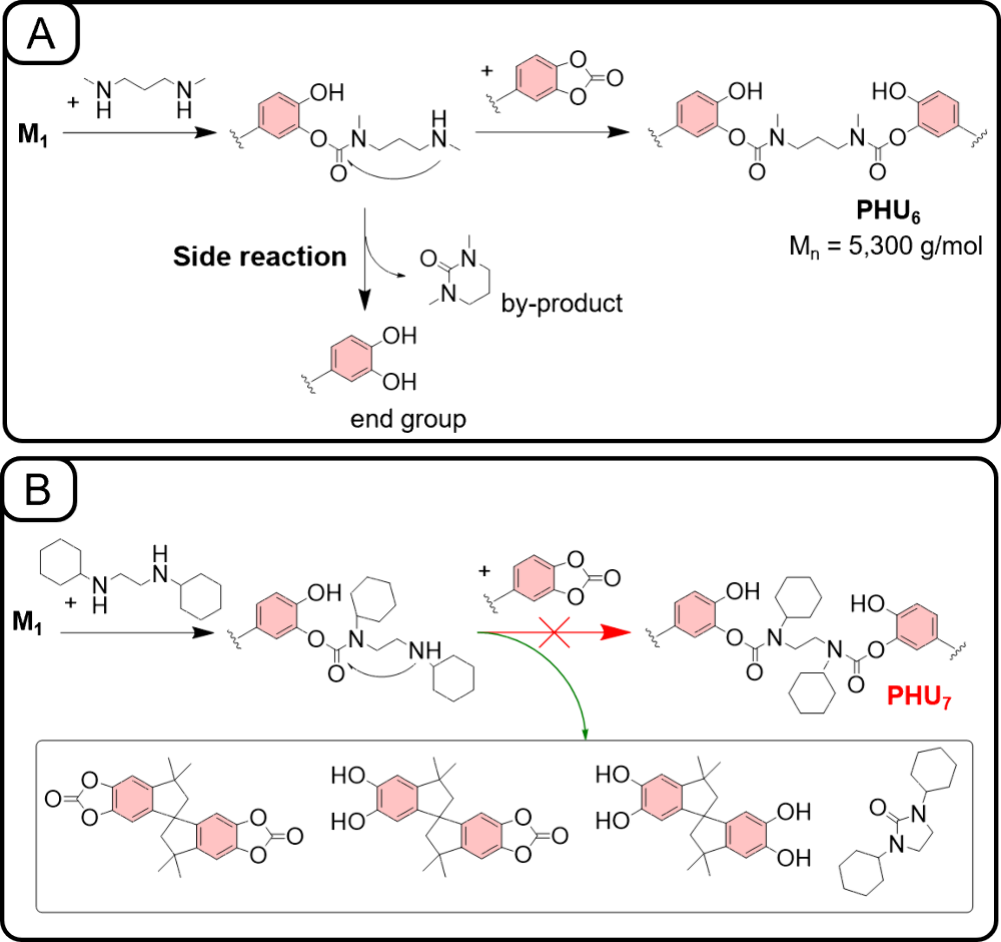
Suggested mechanisms of side reactions during the synthesis of **PHU**_**6**_ (A) and **PHU**_**7**_ (B) from linear secondary diamines.

The detailed analysis of the polymer microstructure and specifically the type of urethane linkages present in the polymer backbone may have provided additional insights on the reaction of cyclic dicarbonate **M**_**1**_ with different diamines. However, although the individual signals of each isomeric product could be clearly identified in the model reactions (Scheme S3, Figure S11), the resolution of the ^1^H NMR spectra obtained for the corresponding polymers was insufficient to allow unambiguous assignment and, in particular, reliable integration of the aromatic proton resonances in the 6–7 ppm region (Figures S15, S17, S19, S21, S23, and S25). To complement the NMR investigation, FTIR spectroscopy was performed on representative PHUs (Figure S31). For **PHU**_**8**_, two principal absorption bands were observed at 1604 cm^–1^ (amide II) and 1699 cm^–1^ (amide I), respectively. In the cases of **PHU**_**1**_ and **PHU**_**9**_, these bands were slightly shifted to 1614 cm^–1^ (amide II) and 1702 cm^–1^ (amide I, hydrogen-bonded C=O), accompanied by the appearance of a shoulder at 1720 cm^–1^, which can be assigned to free (non-hydrogen-bonded) amide I carbonyl groups.^69^ A recent report focused on identification and quantification of urethane and urea groups via FTIR,^69^ and these absorptions can be confidently attributed to hydrogen-bonded and nonbonded urethane groups. In contrast, no spectroscopic evidence for urea formation was detected (see, for comparison, the FTIR spectra of 1.3-diethylurea in Figure S32).

In general, the synthesis of PHUs via polyaddition at elevated temperatures is accompanied by a substantial number of side reactions.^42,70^ In the present work, the mesomeric effect of the aromatic rings in monomer **M**_**1**_ enhanced the electrophilicity of its cyclic carbonate groups, thereby increasing their reactivity.^59^ At the same time, the alicyclic amines, which are more basic than their linear analogues, readily reacted with the activated biscyclic carbonates of **M**_**1**_ even at room temperature. As a result, the polyaddition reaction temperature could be significantly reduced from the conventional 100–120 °C^5–8^ to 50 °C in this study. This significant reduction in temperature likely suppressed side reactions, enabling high conversions and affording PHUs with high molecular weights.

To further test the hypothesis presented above, a second aromatic BCC, designated as **M**_**2**_, was synthesized and included in this study (Figure 3D). The design of **M**_**2**_ was based on the incorporation of catechol fluorene, a representative of the monomers used in the synthesis of so-called cardo-polymers (a term introduced by Vygodskii^71^) in which cyclic units are fused to a single atom within the polymer backbone. The incorporation of cardo groups, such as the fluorene moiety in **M**_**2**_, typically imparts heterochain polymers with enhanced thermal stability, excellent solubility, and high glass-transition temperatures (*T*_g_).^71–73^ **M**_**2**_ was subjected to polyaddition with the two diamines that had previously yielded the highest-molecular-weight PHUs with **M**_**1**_, namely, piperazine and 1,3-di­(piperidin-4-yl)­propane (Figure 3D). The resulting polymers, **PHU**_**8**_ and **PHU**_**9**_, exhibited high molecular weights of 100 and 29 kg/mol (Table 1 and Figure S30), respectively, although these values were lower than those of the corresponding **PHU**_**1**_ and **PHU**_**3**_ (Table 1). This reduction can likely be attributed to the spatial orientation of **M**_**2**_, which results in some steric hindrance between the aromatic rings bearing cyclic carbonate groups.

Overall, the careful selection of diamines enabled, in this work, the synthesis of high-molecular-weight PHUs with DP_n_ values ranging from 45 to 220 monomer units. Notably, four of the obtained polymers (**PHU**_**1**_, **PHU**_**2**_, **PHU**_**3**_, and **PHU**_**8**_) can be ranked among the five highest-molecular-weight linear NIPUs reported to date (Table S1).

### PHU Thermal Properties

Thermal stability tests of the synthesized PHUs in air revealed onset weight-loss temperatures (*T*_onset_) in the range of 200–265 °C (Table 1 and Figure S54). PHUs obtained from diamines in which the two cyclic amine groups were separated by a flexible alkyl spacer showed the highest degradation temperatures in air (**PHU**_**3**_: 260 °C and **PHU**_**9**_: 265 °C). Thermal stability measured under an inert atmosphere (N_2_) was higher by 15–35 °C (Figure S54).

DSC and TMA analyses were used to determine the glass transition temperatures (*T*_g_) of the PHUs (Table 1 and Figures S39–S53). All samples exhibited high *T*_g_ values in the range of 193–263 °C. The highest *T*_g_ values were observed for PHUs synthesized from diamines in which both nitrogen atoms were incorporated within the same alicyclic ring. In contrast, PHUs derived from diamines with two rings separated by a flexible spacer (**PHU**_**3**_ and **PHU**_**9**_) showed the lowest *T*_g_ values.

For a polymer to be melt-processable, it must be possible to exceed both its *T*_g_ and, where applicable, its crystalline melting temperature (*T*_m_) without approaching temperatures at which significant thermal degradation occurs. All PHUs synthesized in this work are fully amorphous (Figures S47–S53), meaning that they do not exhibit a distinct melting transition but instead undergo gradual softening over a broad temperature range above their *T*_g_. For such amorphous systems, the effective melt-processing window is defined by the temperature interval between *T*_g_ and the onset of thermal degradation (*T*_onset_). As summarized in Table 1, most of the PHUs prepared display *T*_g_ values that are close to or even exceed their *T*_onset_, effectively eliminating a practical processing window. The only exceptions are **PHU**_**3**_, **PHU**_**6**_, and **PHU**_**9**_. However, **PHU**_**6**_ possesses a molecular weight that is too low to be of practical relevance for melt processing. Thus, **PHU**_**3**_ and **PHU**_**9**_ remain the only viable candidates for melt-processable materials within this series, thereby demonstrating the importance of selecting/further designing diamines when applying this PHU synthesis method.

## Conclusions

In this work, two activated aromatic bis­(cyclic carbonate) monomers (**M**_**1**_ and **M**_**2**_) were synthesized in high yield and purity, as confirmed by NMR, IR, and elemental analyses. Model reactions of **M**_**1**_ with secondary diamines revealed that steric hindrance and electronic effects play a decisive role in reactivity, with alicyclic secondary amines significantly outperforming linear analogues in this regard. In particular, five- to seven-membered cyclic amines exhibited rapid (within minutes) and quantitative conversions at 50 °C, highlighting their potential for efficient PHU synthesis. Guided by these results, piperazine was identified as a promising partner for polyaddition with **M**_**1**_, enabling the synthesis of PHUs under mild, catalyst-free conditions. Optimization of reaction parameters established DMSO as the preferred solvent, affording high yields and record-high molecular weights of up to *M*_n_ ≈ 105 kg/mol (DP_n_ ≈ 220).

The approach was successfully extended to other secondary alicyclic diamines, confirming that this structural motif is critical for achieving high molecular weights, while linear diamines afforded only oligomeric or low-molecular-weight PHUs, likely due to cyclization-type side reactions. The enhanced electrophilicity of **M**_**1**_, combined with the high basicity of alicyclic amines, allowed the polyaddition to proceed efficiently at 50 °C, a substantial reduction compared to conventional routes that typically require 100–120 °C. The synthesis of **M**_**2**_, containing a cardo fluorene structure, further demonstrated the versatility of this strategy for the synthesis of high molecular weight PHUs.

Overall, these findings highlight the crucial influence of both bis­(cyclic carbonate) and diamine selection on the polyaddition rate and molecular weight of the resulting PHUs. A novel strategy was proposed for the rapid synthesis of PHUs with record-high molecular weights based on the activation of cyclic carbonate monomers through fusion with aromatic rings followed by reaction with secondary alicyclic amines. This approach offers a promising pathway to address one of the major challenges hindering the industrial application of NIPUs.

## Supplementary Material



## Notes

Detailed materials and methods, along with the synthesis and characterization of two aromatic bis­(cyclic carbonate) monomers (**M**_**1**_ and **M**_**2**_) and their corresponding polymers (PHUs); TMA, DSC, and TGA analyses of PHUs, as well as their molecular weight determination by GPC, sedimentation–diffusion analysis, and intrinsic viscosity measurements; an investigation of model reactions and their kinetics, together with a comparison of the reported molecular weights and degrees of polymerization for linear NIPUs available in the literature to date (PDF)
