# Supplementary material for: Highly Efficient Approach to High-Molecular Weight Polyhydroxyurethanes
Source: Macromolecules. 2026 Apr 24;59(9):5140–50. doi: 10.1021/acs.macromol.5c02932 (PMC13174187; doi:10.1021/acs.macromol.5c02932)
Supplement: Supplementary file 1 [file ma5c02932_si_001.pdf]

Supporting information for

Highly efficient approach to high-molecular weight polyhydroxyurethanes

Sergei V. Zubkevich*,†, Abdurrahman Beter†, Arpan Datta Sarma†, Reiner Dieden†, Vincent Berthé†, Alexander S. Shaplov*,† and Daniel F. Schmidt*,†

† Abdurrahman Beter, Sergei V. Zubkevich, Vincent Berthe, Reiner Deiden, Alexander S. Shaplov, Daniel F. Schmidt
Luxembourg Institute of Science and Technology (LIST)
5 Avenue des Hauts-Fourneaux, L-4362 Esch-sur-Alzette, Luxembourg
E-mail: S.Z.: zubkevich.sergey@gmail.com; A.S.: alexander.shaplov@list.lu; D.S.: daniel.schmidt@list.lu.

Table of Contents

I. Materials 2
II. Methods 2
III. Previously reported high molecular weight NIPUs. 5
IV. Synthesis and characterization of cyclic carbonates. 12
III.1. 7,7,7',7'-tetramethyl-6,6',7,7'-tetrahydro-5,5'-spirobi[indeno[5,6-d][1,3]dioxole]-2,2'-dione (**M₁**). 12
III.2. 5,5'-(9H-fluorene-9,9-diyl)bis(benzo[d][1,3]dioxol-2-one) (**M₂**). 15
V. Kinetic experiments with various monoamines 18
V.1. Model reaction between **M₁** and piperidine 20
VI. Synthesis of PHUs. 23
VI.1. General method. 23
VI.2. Synthesis of PHU based on **M₁** and piperazine (**PHU₁**). 23
VI.3. Synthesis of PHU based on **M₁** and homopiperazine (**PHU₂**). 25
VI.4. Synthesis of PHU based on **M₁** and 1,3-di(piperidin-4-yl)propane (**PHU₃**) 26
VI.5. Synthesis of PHU based on **M₁** and octahydro-1H-pyrrolo[3,4-b]pyridine (**PHU₄**). 28
VI.6. Synthesis of PHU based on **M₁** and 3,3'-bipiperidine (**PHU₅**) 29
VI.7. Synthesis of PHU based on **M₁** and N^1,N^3-dimethylpropane-1,3-diamine (**PHU₆**) 31
VI.8. Attempted synthesis of PHU based on **M₁** and N^1,N^2-dicyclohexylethane-1,2-diamine (**PHU₇**) 32
VI.9. Synthesis of PHU based on **M₂** and piperazine (**PHU₈**). 33
VI.10. Synthesis of PHU based on **M₂** and 1,3-di(piperidin-4-yl)propane (**PHU₉**) 34
VII. Solubility of the obtained PHUs. 38
VIII. Possible side reactions during polyaddition of linear secondary diamines. 39
IX. Molecular weight determination via GPC 42
X. Molecular weight determination via sedimentation-diffusion analysis. 42
XI. TMA plots of PHUs. 43
XII. DSC plots of PHUs. 47
XIII. References 53

I. Materials

***Reagents**:* 5,5',6,6'-Tetrahydroxy-3,3,3',3'-tetramethyl-1,1'-spirobiindane (97 %, BLD Pharmatech), 4,4'-(9H-fluorene-9,9-diyl)bis(benzene-1,2-diol) (98 %, BLD Pharmatech), triethylamine (≥ 99 %, Sigma-Aldrich), bis(trichloromethyl)carbonate (> 96 %, Sigma-Aldrich), phosphorous pentoxide ((immobilized on silica, with indicator, Sicapent®, Sigma-Aldrich), lithium bis(trifluoromethanesulfonyl)imide (LiTFSI, 99.9 %, Solvionic), sodium hydroxide (> 98 %, Carl Roth) were used as received.

Diethylamine (> 99.5 %, Sigma-Aldrich), diisopropylamine (> 99.5 %, Sigma-Aldrich), diisobutylamine (99 %, Sigma-Aldrich), dicyclohexylamine (99 %, Sigma-Aldrich), N-methylaniline (> 98 %, TCI Europe), N-methylcyclohexylamine (> 99 %, TCI Europe), azetidine (98 %, BLD Pharmatech), pyrrolidine (≥ 99.5 %, Sigma-Aldrich), piperidine (≥ 99.5 %, Sigma-Aldrich), hexamethyleneimine (99 %, Sigma-Aldrich), 2,2,6,6-tetramethylpiperidine (98 %, BLD Pharmatech) were used for model reactions and were vacuum distilled with sodium hydroxide before using.

Piperazine (> 98 %, TCI Europe), homopiperazine (98 %, BLD Pharmatech), 1,3-di(piperidin-4-yl)propane (98 %, BLD Pharmatech), octahydro-1H-pyrrolo[3,4-b]pyridine (97 %, BLD Pharmatech), N^1,N^3-dimethylpropane-1,3-diamine (98 %, BLD Pharmatech), N,N'-dicyclohexyl-1,2-ethanediamine (> 98 %, TCI Europe) and 3,3'-bipiperidine (> 95 %, BLD Pharmatech) were vacuum distilled with sodium hydroxide before using.

Solvents: Isopropanol (> 99.5 %, SLR, Extra Pure, Fisher Scientific (Acros Organics)), methanol (> 99.5 %, Fisher Scientific (Acros Organics)), tetrahydrofuran (THF, 99.6 %, Fisher Scientific (Acros Organics)), dichloromethane (> 99.5 %, Fisher Scientific (Acros Organics)), diethyl ether (99+ %, Fisher Scientific (Acros Organics)), dimethylsulfoxide (DMSO, ≥ 99.9 %, anhydrous, Sigma-Aldrich), 1-methyl-2-pyrrolidone (NMP, 99.5 %, Extra Dry, AcroSeal, Fisher Scientific (Acros Organics)), dimethylformamide (99.7 %, HPLC grade, Fisher Scientific (Acros Organics)), dimethylformamide (99 %, extra pure, Fisher Scientific (Acros Organics)), N,N-dimethylacetamide (99.5 %, Fisher Scientific (Acros Organics)), sulfolane (99 %, Sigma-Aldrich), hexamethylphosphoramide (≥ 98 %, Sigma Aldrich), acetone (technical grade, Fisher Scientific (Acros Organics)), chloroform (99 %, Fisher Scientific (Acros Organics)), 1,1,2-trichloroethane (97 %, Sigma-Aldrich), acetonitrile (99.9 %, Extra Dry over Molecular Sieve, AcroSeal™, Fisher Scientific (Acros Organics)), cyclohexane, (99.5 %, Sigma-Aldrich), cyclohexanone (99.8 %, extra pure, Fisher Scientific (Acros Organics)), ethyl acetate (99.9 %, Fisher Scientific (Acros Organics)), toluene (99+ %, Fisher Scientific (Acros Organics)), dioxane (≥ 99.5 %, Carl Roth) were procured and used as received. Tetrahydrofuran (THF, 99.6 %, Fisher Scientific (Acros Organics)) used to synthesize cyclic carbonates was dried through SPS (MBRAUN, SPS-800) system before use. Ultrapure deionized water was obtained using Sartorius Arium® Comfort smart station.

NMR solvents and standards: dimethyl sulfoxide-d_6 (DMSO-d_6, 99.9 atom%D, Sigma-Aldrich) was dried over 3Å molecular sieves.

Gases: Argon alpha gas 2 (99.99999 %, Air Liquide) was used as received.

II. Methods

I.2.1. Nuclear magnetic resonance (NMR) spectra were recorded on an Avance III HD 600MHz (Bruker) spectrometer (^{1}H NMR at 600 MHz, ^{13}C NMR at 151 MHz) at 25°C (unless stated otherwise) in the indicated deuterated solvent and are listed in ppm. The signals corresponding to the residual protons and carbons of the deuterated solvent were used as an internal standard for ^{1}H and ^{13}C NMR, respectively. Traces of common non-deuterated solvents and impurities were identified according to *Fulmer et al.*[1]

I.2.2. Size exclusion chromatography (SEC) / gel permeation chromatography (GPC) was used to determine the number-average molecular weights ($M_{n(GPC)}$) and M_w/M_n ratios. Studies were performed using a 1200 Infinity gel permeation chromatograph (Agilent Technologies, USA) equipped with a PLgel 5µm MIXED-D column (Agilent Technologies, USA), PLgel 5µm (Agilent Technologies) pre-column and an integrated refractive index detector.

The system was operated at 50°C and 1.0 mL/min flow using 0.1 M $Li(CF_3SO_2)_2N$ (LiTFSI) solution in DMF as the eluent. Poly(methyl methacrylate) standards (EasiVial PM, Agilent Technologies, M_p = 0.55-1558 kg/mol) were used to perform calibration.

Degree of polymerization (DP_n) was calculated with the GPC data using the following equation:

$$DP_n = {M_n}/{(M_{CC} + M_{amine})} \qquad \textbf{(eq.S1)}$$

Where M_n is the number average molecular weight of PHU polymer determined using GPC, M_{CC} is the molecular weight of the cyclic dicarbonate monomer and M_{amine} is the molecular weight of the amine monomer used in the synthesis of various PHU polymers.

I.2.3. Determination of molecular weights by sedimentation-diffusion analysis was performed using a Beckman XLI analytical ultracentrifuge (ProteomeLabTM Protein Characterization System) in a two-sector cell with an optical path length of 12 mm at a rotor speed of 40000 rpm. Both $\mathbf{PHU_1}$ and $\mathbf{PHU_3}$ investigated in this study were purified by precipitation and subsequently dried under mentioned conditions prior to measurement of their molecular weight (M_{sD}). Importantly, the samples analyzed were taken from the same batches as those used for the corresponding GPC measurements.

To account for the concentration dependence of the sedimentation coefficient, experiments were conducted at three solution concentrations ranging from 0.38 to 0.1 g/dL. The sedimentation coefficients s_0 at infinite dilution were calculated from the plot described by the formula:

$$s^{-1}=s_0^{-1}(1+k_s c) \qquad \textbf{(eq.S2)}$$

where s is the sedimentation coefficient at a given concentration and k_s is the Gralen concentration coefficient.

Translational diffusion was studied using a Tsvetkov polarization-interferometric diffusometer at concentrations of 0.06 g/dL. Viscosimetric studies were conducted in an Ostwald capillary viscometer over a range of concentrations corresponding to relative flow times of the solution and solvent of 2–1.15. The intrinsic viscosity values were calculated from extrapolation of Huggins and Kraemer plots to zero concentration.

Absolute molecular masses were calculated using the Svedberg equation with the obtained experimental values of s_0, D_0 and $(1-\upsilon\rho_0)$:

$$M_{sD} = \frac{RT}{(1-\upsilon\rho_0)} \cdot \frac{s_0}{D_0} \qquad \textbf{(eq.S3)}$$

where s_0 is the sedimentation coefficient at infinite dilution, D_0 is the translational diffusion coefficient, R is the gas constant, υ is the partial specific volume of macromolecules, ρ_0 is the solvent density, T is the absolute temperature in K, and $(1-\upsilon\rho_0)$ is is the buoyancy factor, determined densitometrically. The errors in determining the primary experimental values are no more than 1-2%, while for the molecular mass the error is 4%.

I.2.4. Intrinsic viscosity. Reduced and relative viscosities ($\eta_{red} = \eta_{sp}/c=(\eta_{rel}-1)/c$ with $\eta_{rel}=t/t_o$, where t and t_o are the flow times of the polymer solution and the solvent, respectively; η_{rel} – is a relative viscosity) were determined using an Ubbelohde-type capillary viscometer at 25.0 °C. The intrinsic viscosities $[\eta]$ were obtained by extrapolation to zero concentration according to Huggins[2] and Schulz-Blaschke[3].

I.2.5. Thermogravimetric analysis (TGA) was carried out in air using a TGA2 STARe System (Mettler Toledo) at a heating rate of 5°C/min. The onset weight loss temperature (T_{onset}) was determined as the point in the TGA curve at which a significant deviation from the horizontal was observed. The resulting temperature was then rounded to the nearest 5°C.

I.2.6. Differential Scanning Calorimetry (DSC) was performed on a DSC 300 Caliris Select DSC (NETZSCH). For monomers, two heating-cooling cycles with a heating rate of 5 °C/min were carried out for each sample under a N_2 atmosphere; melting temperature (T_m) was determined from the first heating curve while crystallization temperature (T_{cr}) was determined from the second cooling curve. For simplicity T_m and T_{cr} were taken from the peak maxima. For polymers, three heating-cooling cycles (1st and 2nd at 5 °C/min, 3rd at 10 °C/min) were carried

out for each sample under a N_2 atmosphere; the first cycle served to eliminate the process history of the specimens, while glass transition temperatures (T_g) were determined during the second and third heating cycles at 5 °C/min and 10 °C/min, respectively.

III. Previously reported high molecular weight NIPUs.

Table S1. Comparison of the molecular weights and polymerization degrees (DP_n) for the selected linear NIPUs reported in the literature and for those obtained in the present work (updated on August, 2025).

№	Polymerization approach	Monomer 1	Monomer 2	M_n (g/mol)	M_w/ M_n	DP_n [a]	Note	Ref.
1	ROP of aziridine with carbon dioxide	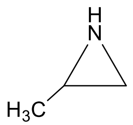	CO_2	150000[b]	n.d.	~1500	Requires supercritical conditions (22 MPa pressure); low yields (<20%); urethane content is only 0.54; M_w by light scattering method; Author notes unexpected GPC result (possible interaction with column).	4,5
2	Polyaddition of 5-BCC/di-amines	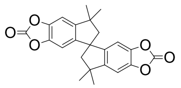	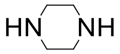	105400	3.60	220	**This work**	
3	Polyaddition of 5-BCC/di-amines	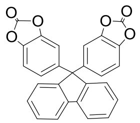	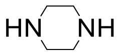	100300	4.3	211	**This work**	
4	Polyaddition of 5-BCC/di-amines	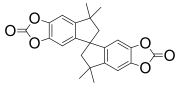	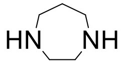	95300	2.8	194	**This work**	
5	Polyaddition of 5-BCC/di-amines	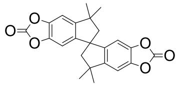	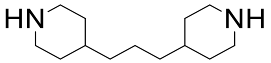	94400	3.8	157	**This work**	

6	Polycondensation of biscarbamates and diols	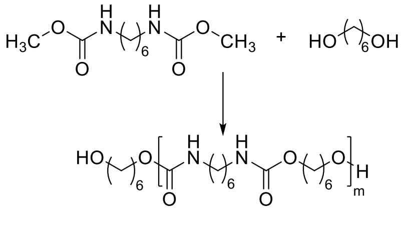	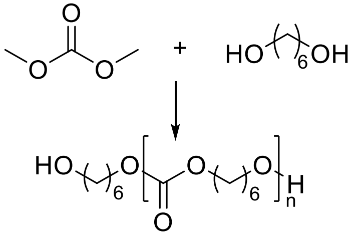	93000	2.00	65.9	Multistep synthesis; Use of oligomeric monomers with unknown degree of polymerization.	6
7	Polyaddition of 5-BCC/di-amines	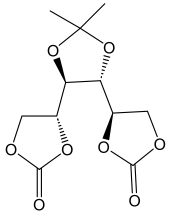	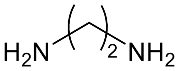	73333	1.50	238	Author notes unexpected GPC results (possible interaction with column)	7
8	Polyaddition of 5-BCC/di-amines	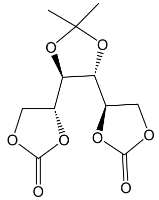	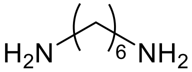	58000	1.50	148	Author notes unexpected GPC result (possible interaction with column)	7
9	Polyaddition of 5-BCC/di-amines	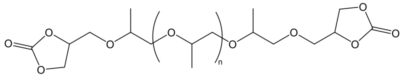 M_n = 630	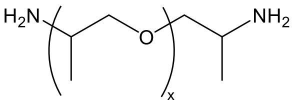 Jeffamine, M_n = 2000	68000	2.30	26		8
10	Polyaddition of 5-BCC/di-amines	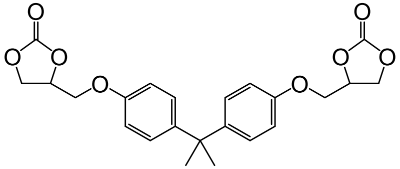	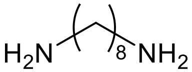	53400	1.38	93		9
11	Polycondensation of biscarbamates and diols	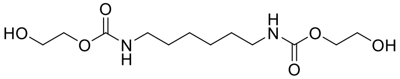	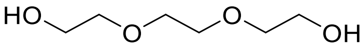	50900	1.84	~115	Requires high temperature (> 170°C) and high vacuum (< 1 mm Hg) to remove the side product (ethylene glycol)	10

12	Polycondensation of biscarbamates and diols	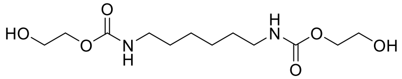	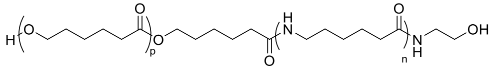	45830	1.48	35	Polyol was obtained from ROP of caprolactone using oligomeric polyamide as initiator	11
13	Polyaddition of 5-BCC/di-amines	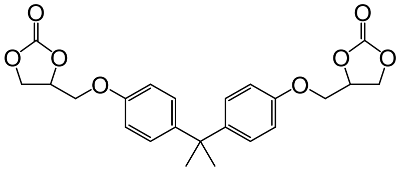	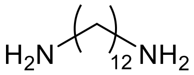	36700	1.84	59		12
14	Polyaddition of 7-BCC/di-amines	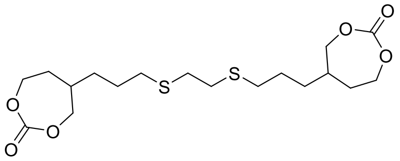	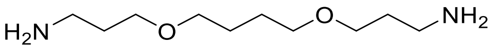	35700	2.80	58		13
15	ROP of cyclic carbamates	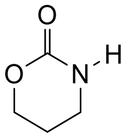	-	32600	2.46	322		14
16	Polyaddition of 5-BCC/di-amines	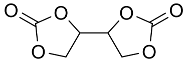	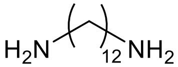	31300	3.10	84		15
17	Polycondensation of biscarbamates and diols	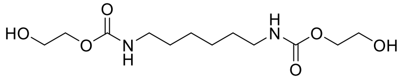	-	30900	2.03	105	Requires high temperature (> 170°C) and high vacuum (< 1 mm Hg) to remove the side product (ethylene glycol)	10
18	Polyaddition of 5-BCC/di-amines	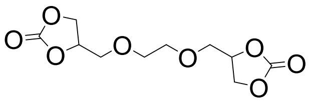	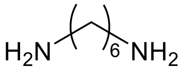	30200	1.22	80		16
19	Polyaddition of 5-BCC/di-amines		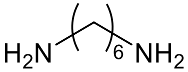	28000	2.04	52		17

		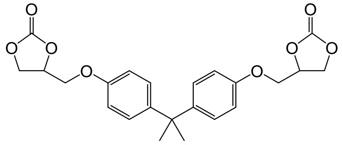						
20	Polycondensation of biscarbonates and diamines	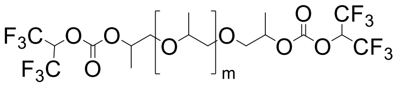	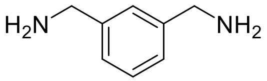	27300	1.68	46		18
21	Polyaddition of 6-BCC/di-amines	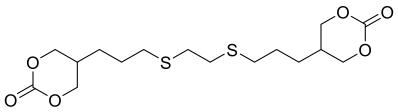	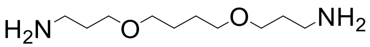	26000	n.d.	45		19
22	Polyaddition of 5-BCC/di-amines	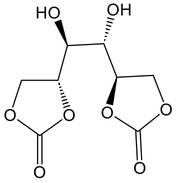	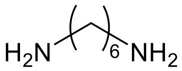	26000	1.80	74		7
23	Polyaddition of activated BCC/secondary di-amines	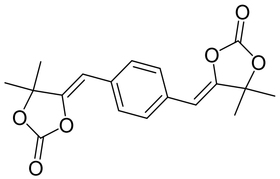	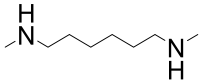	25000	2.92	53		20
24	Polycondensation of biscarbamates and diols	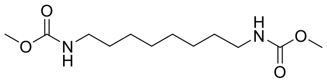	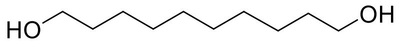	24600	1.95	66	Very complex synthesis of biscarbamates from fatty acids	21
25	Polyaddition of 5-BCC/di-amines	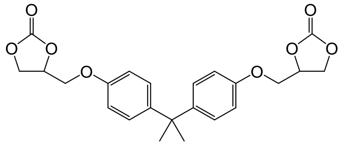	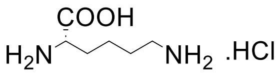	24000	1.58	42		22
26	Polyaddition of 6-BCC/amines	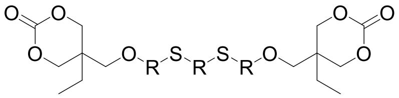	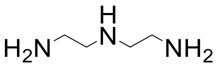	20400	3.1	34		23

27	Polycondensation of biscarbonates and diamines	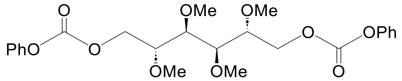	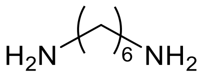	17490	1.99	43		24
28	Polycondensation of biscarbonates and diamines	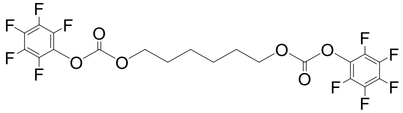	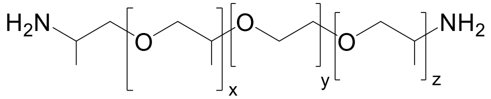 x + z = 6<br>y = 36	15500	1.96	7		25
29	Polycondensation of biscarbamates and diols	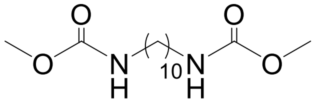	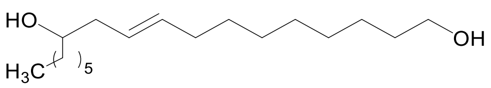	13900	1.90	27		26
30	Polyaddition of 5-BCC and Polycondensation of carbonates/di-amines	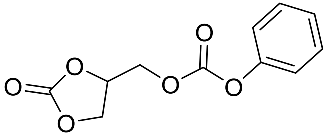	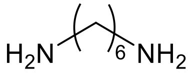	13700	1.57	53		27
31	Polyaddition of 5-BCC/di-amines	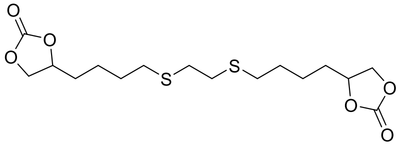	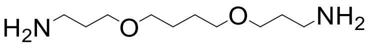	12000	n.d.	21		19
32	Self-polyaddition of 6-BCC/amines	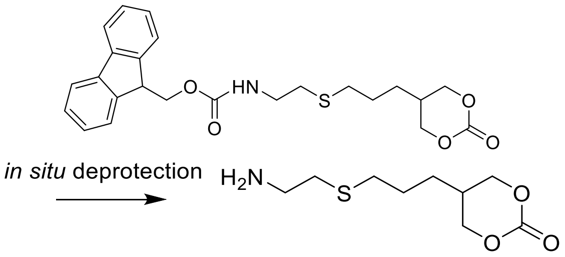		11000	1.20	51		28

33	Curtius rearrangement of acyl azides	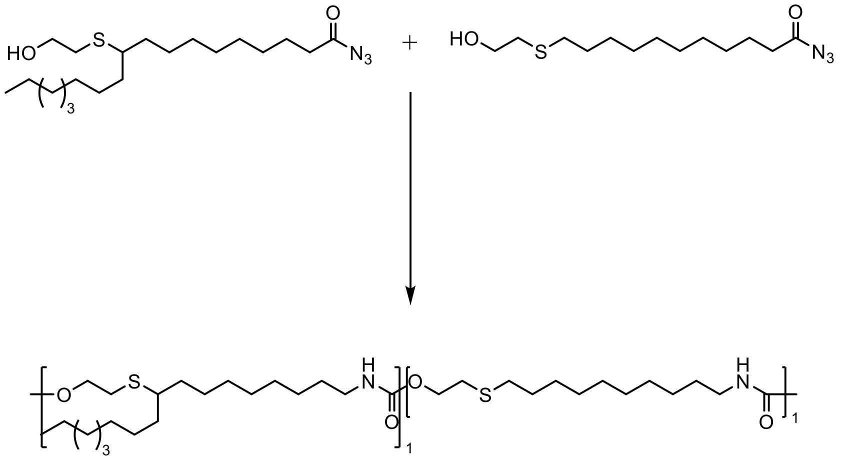		10400	1.91	31	These transformations generate isocyanates *in situ*, which subsequently react with alcohols to form PUs.	29
34	Polyaddition of 5-BCC/di-amines	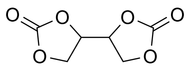	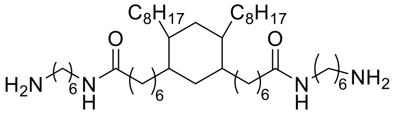	9000	2.91	10		15
35	Polycondensation of biscarbonates and diamines	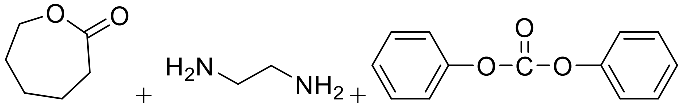		7900	1.38	48		30
36	Polycondensation of biscarbamates and diols	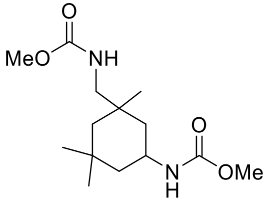	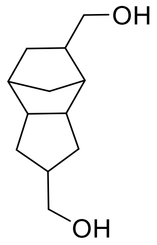	7800	2.40	19		31

37	ROP of cyclic carbamates	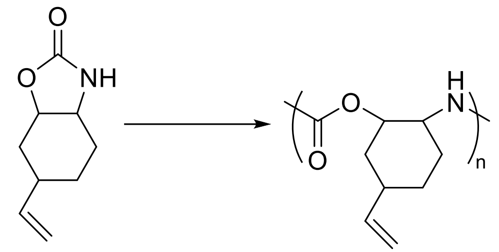		7500	1.32	46		32
38	Curtius rearrangement of acyl azides	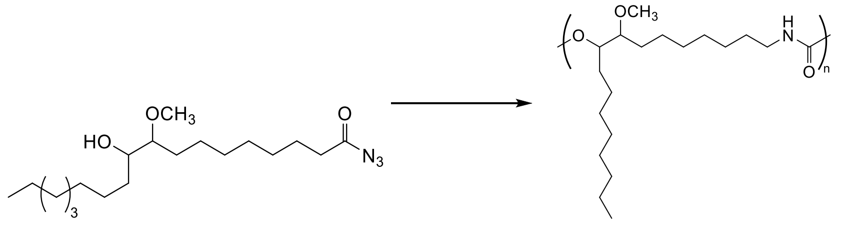		6880	1.50	20	These transformations generate isocyanates *in situ*, which subsequently react with alcohols to form PUs.	33
39	ROP of aziridine with carbon dioxide	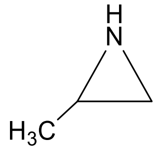	CO_2	605	n.d.	~6	Requires high pressure (60 atm); M_n by osmometry.	34

[a] Calculated as the ratio of the GPC-measured M_n to the molar mass of the repeating unit. [b] M_w.

IV. Synthesis and characterization of cyclic carbonates.

III.1. 7,7,7',7'-tetramethyl-6,6',7,7'-tetrahydro-5,5'-spirobi[indeno[5,6-d][1,3]dioxole]-2,2'-dione (**M₁**).

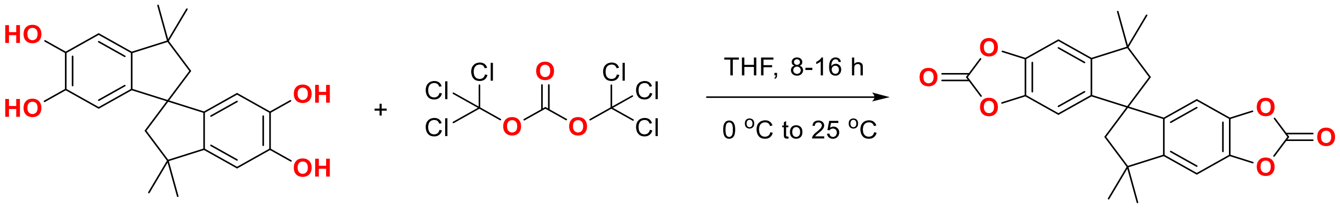

Scheme S1. Synthesis of 7,7,7',7'-tetramethyl-6,6',7,7'-tetrahydro-5,5'-spirobi[indeno[5,6-d][1,3]dioxole]-2,2'-dione (**M_1**).

5,5',6,6'-Tetrahydroxy-3,3,3',3'-tetramethyl-1,1'-spirobiindane (10 g, 29.4 mmol) was dissolved in 200 ml of anhydrous THF in a 2-neck round bottom flask flushed with argon. Triethylamine (18 mL, 13.08 g, 129.3 mmol) was added to the solution via syringe and the resulting mixture was cooled to 0°C using ice bath. The mixture was kept under inert atmosphere by slowly purging argon through the flask. Then bis(trichloromethyl) carbonate (6.39g, 21.5 mmol, 1.1 equiv. per functional group) dissolved in 50 mL of anhydrous THF was added dropwise to the stirred solution. The reaction was stirred for at least 8 hours slowly allowing it to reach room temperature (ca. 22°C). Then the yellowish precipitate of triethylammonium chloride was removed by filtration and washed with two portions of regular THF (50 mL each). The combined THF solutions were evaporated to dryness in vacuo and the crude mixture was redissolved in DCM. The final product was obtained overnight in the form of white crystalline powder by recrystallization from DCM/diethyl ether mixture in freezer (–18°C). It was isolated by filtration, washed by diethyl ether and dried in vacuum (< 0.5 mbar) at 80°C overnight. Yield: 7.8 g (67.7%). T_{onset} (TGA, 5°C/min, on air) = 230°C; T_{onset} (TGA, 5°C/min, N_2) = 275°C. Melting point (DSC, 5°C/min, sealed under N_2) T_m = 269.3°C. Crystallization (DSC, 5°C/min, sealed under N_2) T_c = 147.3°C. Anal. calcd. for $C_{23}H_{20}O_6$ (392.41): C, 70.40%; H, 5.14%; O, 24.46%. Found: C, 70.36%; H, 5.19%.

<u>^{1}H NMR (600 MHz, DMSO-d_6)</u> δ 7.46 (s, 2H), 6.79 (s, 2H), 2.38 (d, *J* = 13.1 Hz, 2H), 2.20 (d, *J* = 13.0 Hz, 2H), 1.41 (s, 6H), 1.32 (s, 6H).

<u>^{13}C NMR (151 MHz, DMSO-d_6)</u> δ 151.33, 148.32, 145.51, 142.57, 142.37, 105.80, 104.59, 58.47, 57.64, 43.47, 31.34, 29.84.

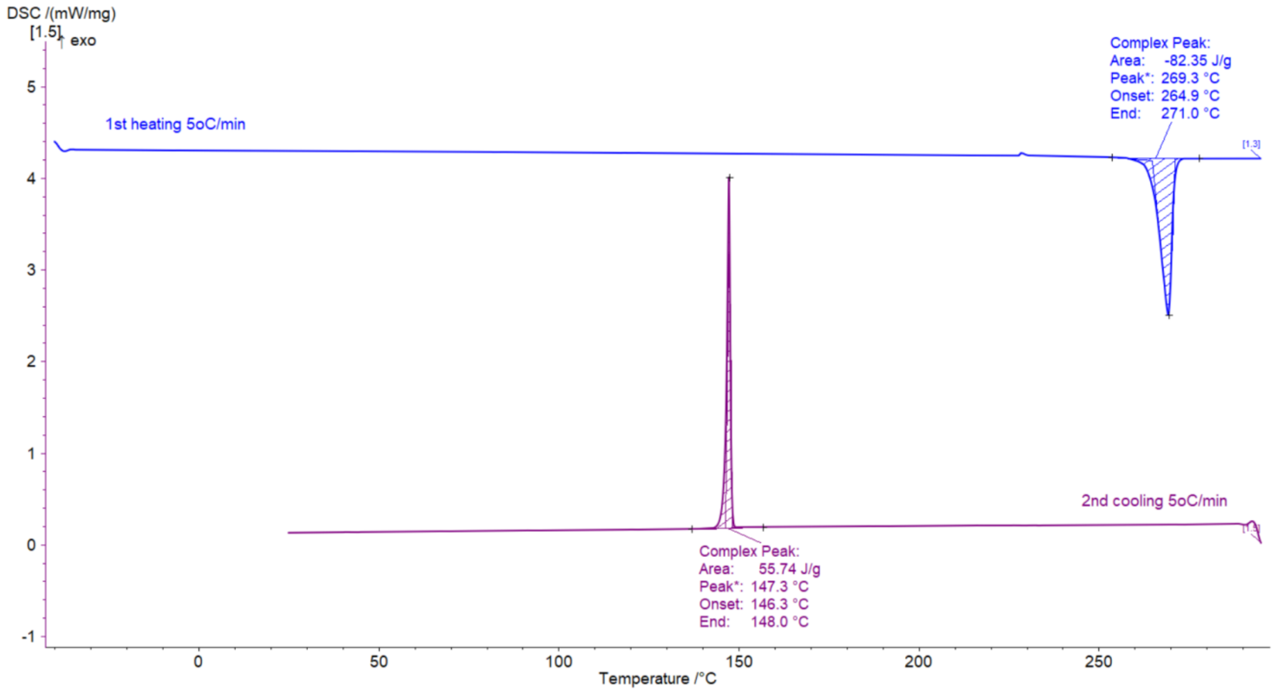

Figure S1. DSC plots of **M_1**.

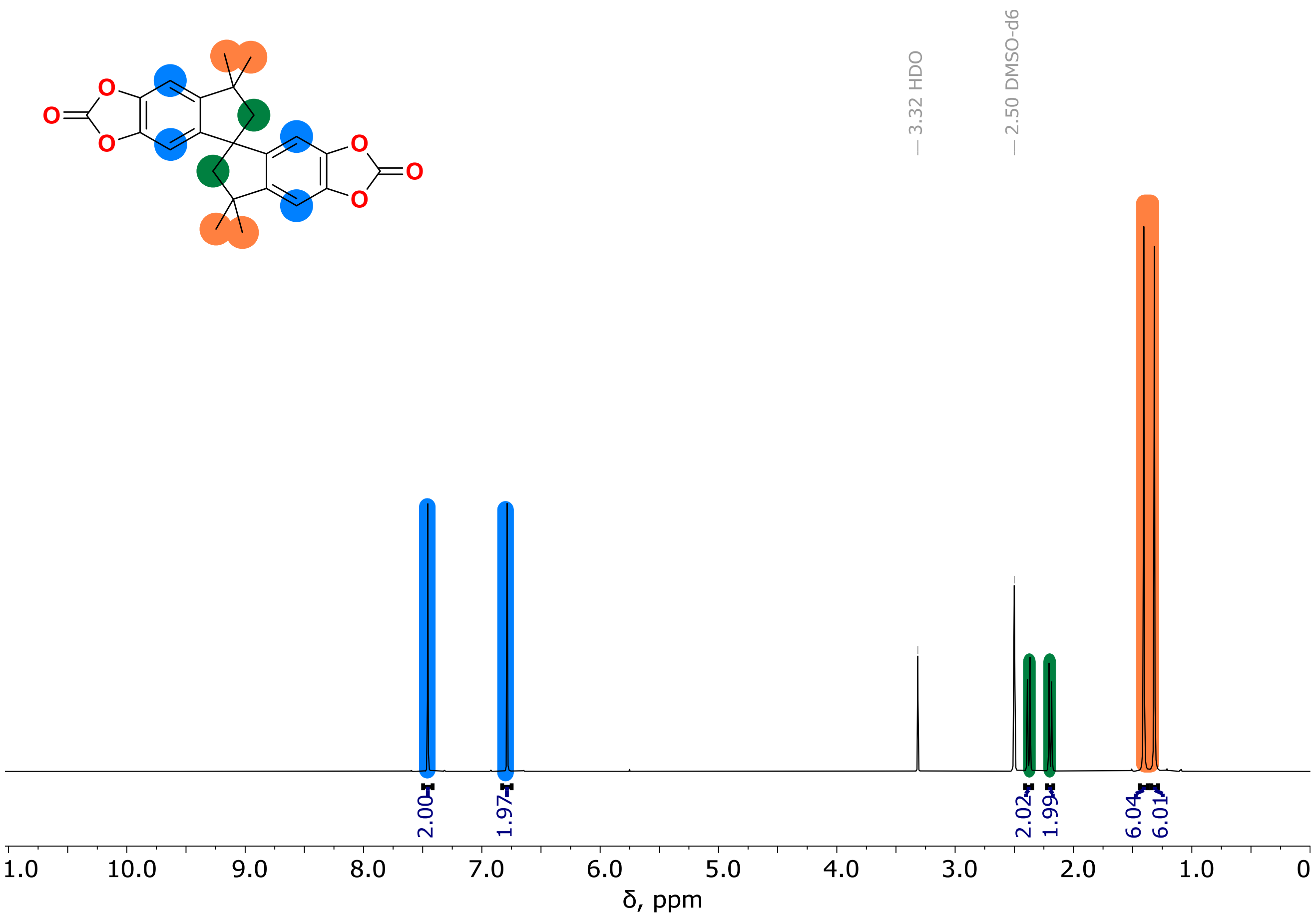

Figure S2. ^{1}H NMR spectrum of $\mathbf{M_1}$ in DMSO-d$_6$.

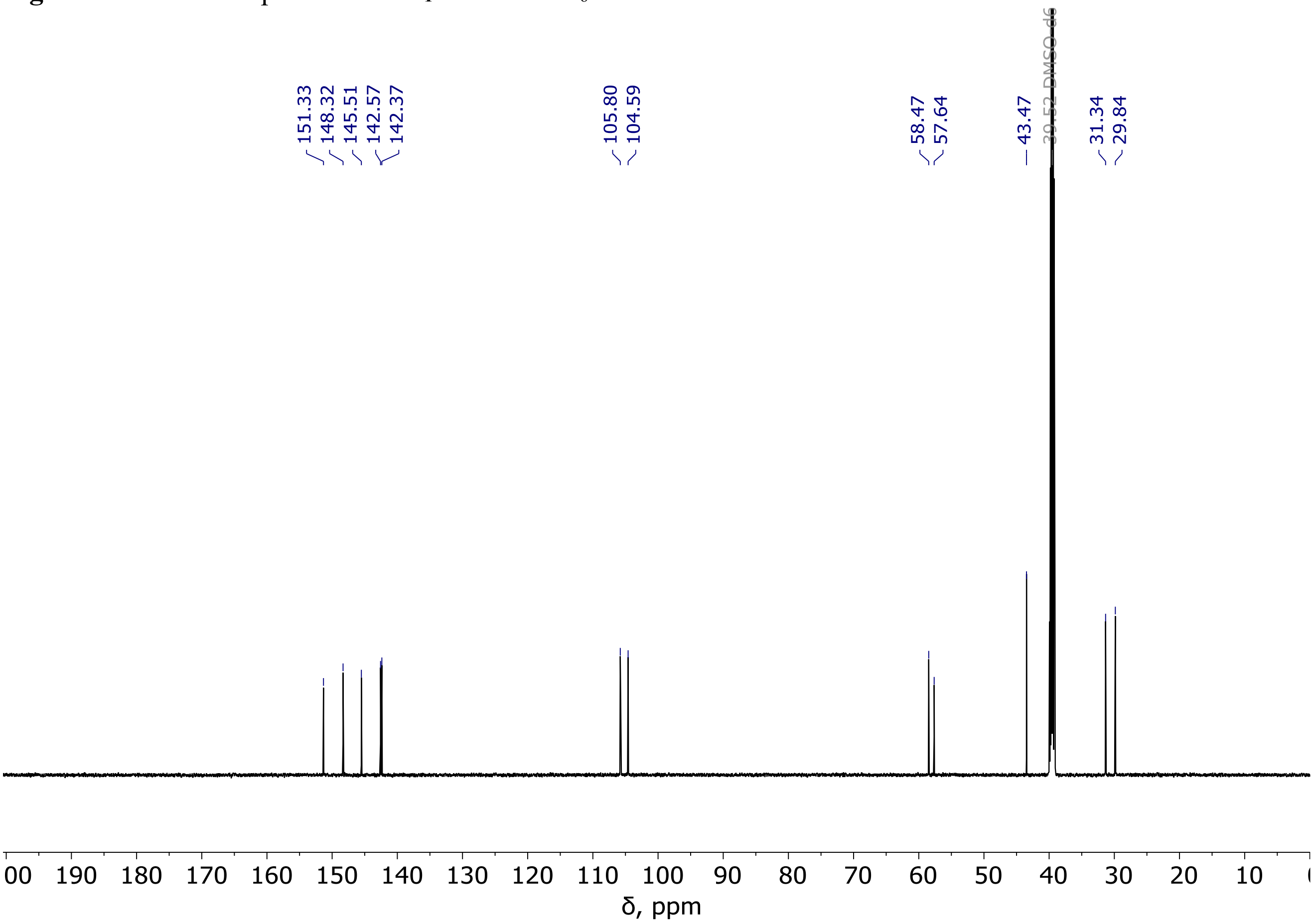

Figure S3. ^{13}C NMR spectrum of $\mathbf{M_1}$ in DMSO-d$_6$.

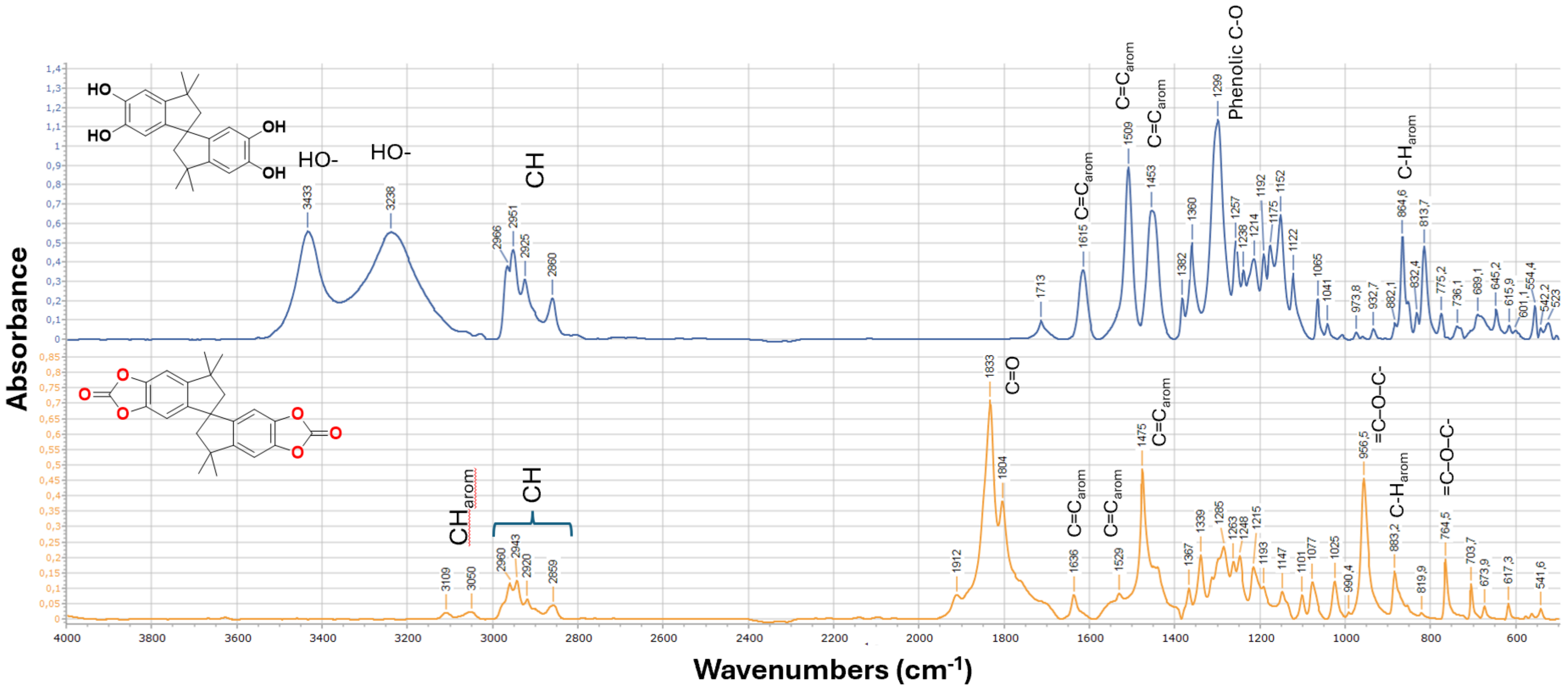

Figure S4. IR spectrum of $\mathbf{M_1}$ and its precursor.

III.2. 5,5'-(9H-fluorene-9,9-diyl)bis(benzo[d][1,3]dioxol-2-one) (**M$_2$**).

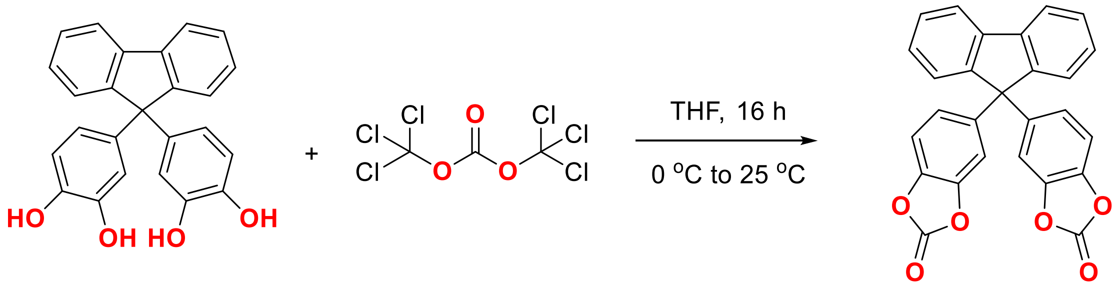

Scheme S2. Synthesis of 5,5'-(9H-fluorene-9,9-diyl)bis(benzo[d][1,3]dioxol-2-one) (**M$_2$**).

5,5'-(9H-fluorene-9,9-diyl)bis(benzo[d][1,3]dioxol-2-one) (**M$_2$**) was prepared in a similar manner to **M$_1$**. The following amounts of 4,4'-(9H-fluorene-9,9-diyl)bis(benzene-1,2-diol) (10 g, 26.1 mmol), triethylamine (16 mL, 11.64 g, 115.1 mmol) and bis(trichloromethyl) carbonate (5.69g, 1.92 mmol, 1.1 equiv. per functional group) were used. The target compound was obtained in the form of white crystals after recrystallization from DCM/diethyl ether mixture. Yield: 10.05 g (88.5%). T_{onset} (TGA, 5°C/min, on air) = 250°C. Melting point (DSC, 5°C/min, sealed under N_2) T_m = 240.0°C. Anal. calcd. for $C_{27}H_{14}O_6$ (434.40): C, 74.65%; H, 3.25%; O, 22.10%. Found: C, 74.60%; H, 3.54%.

^{1}H NMR (600 MHz, DMSO-d$_6$) δ 7.97 (d, *J* = 7.6 Hz, 2H), 7.54 (d, *J* = 7.6 Hz, 2H), 7.45 (td, *J* = 7.5, 1.0 Hz, 2H), 7.36 (dd, *J* = 8.6, 7.2 Hz, 4H), 7.22 (d, *J* = 1.9 Hz, 2H), 6.96 (dd, *J* = 8.5, 1.9 Hz, 2H).
^{13}C NMR (151 MHz, DMSO-d$_6$) δ 150.94, 150.55, 149.41, 143.12, 142.32, 142.04, 139.39, 139.35, 128.30, 128.21, 127.87, 127.74, 126.09, 125.99, 123.54, 120.88, 120.55, 110.10, 109.97, 109.94, 64.63, 64.23.

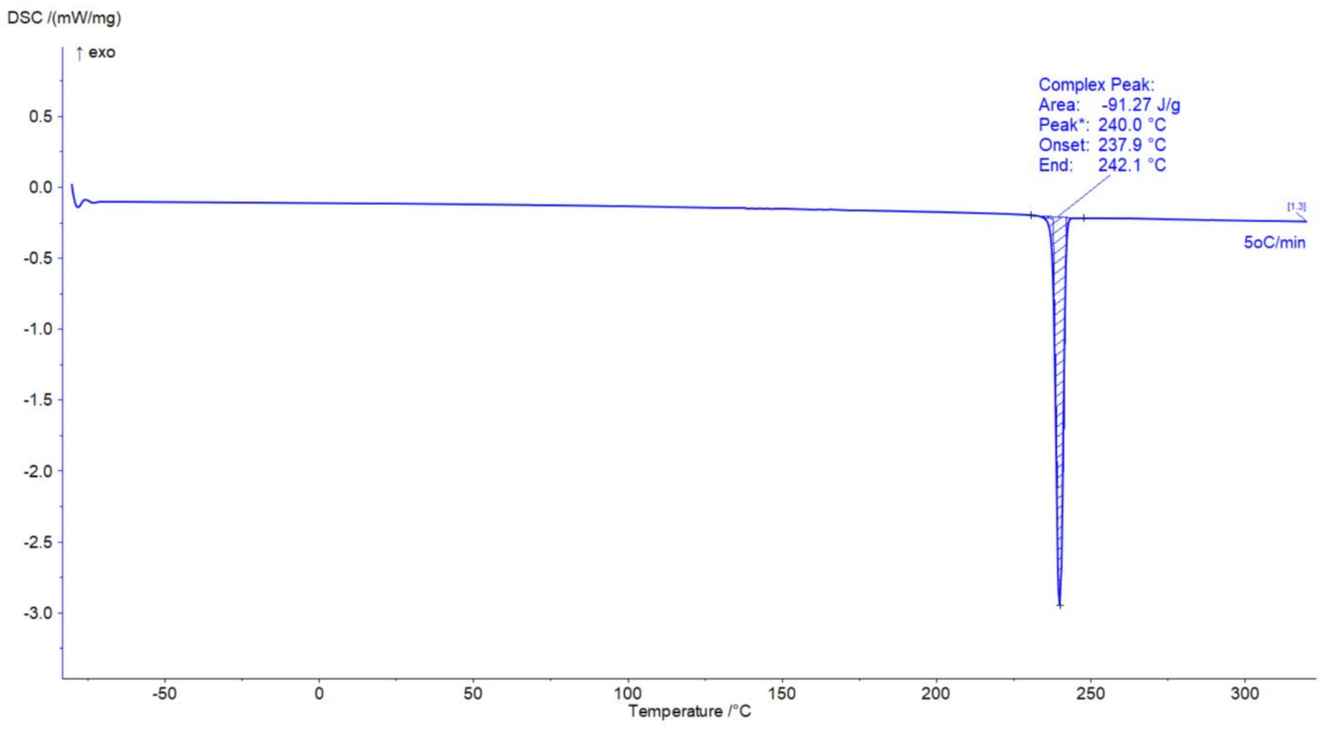

Figure S5. DSC plots of **M$_2$**.

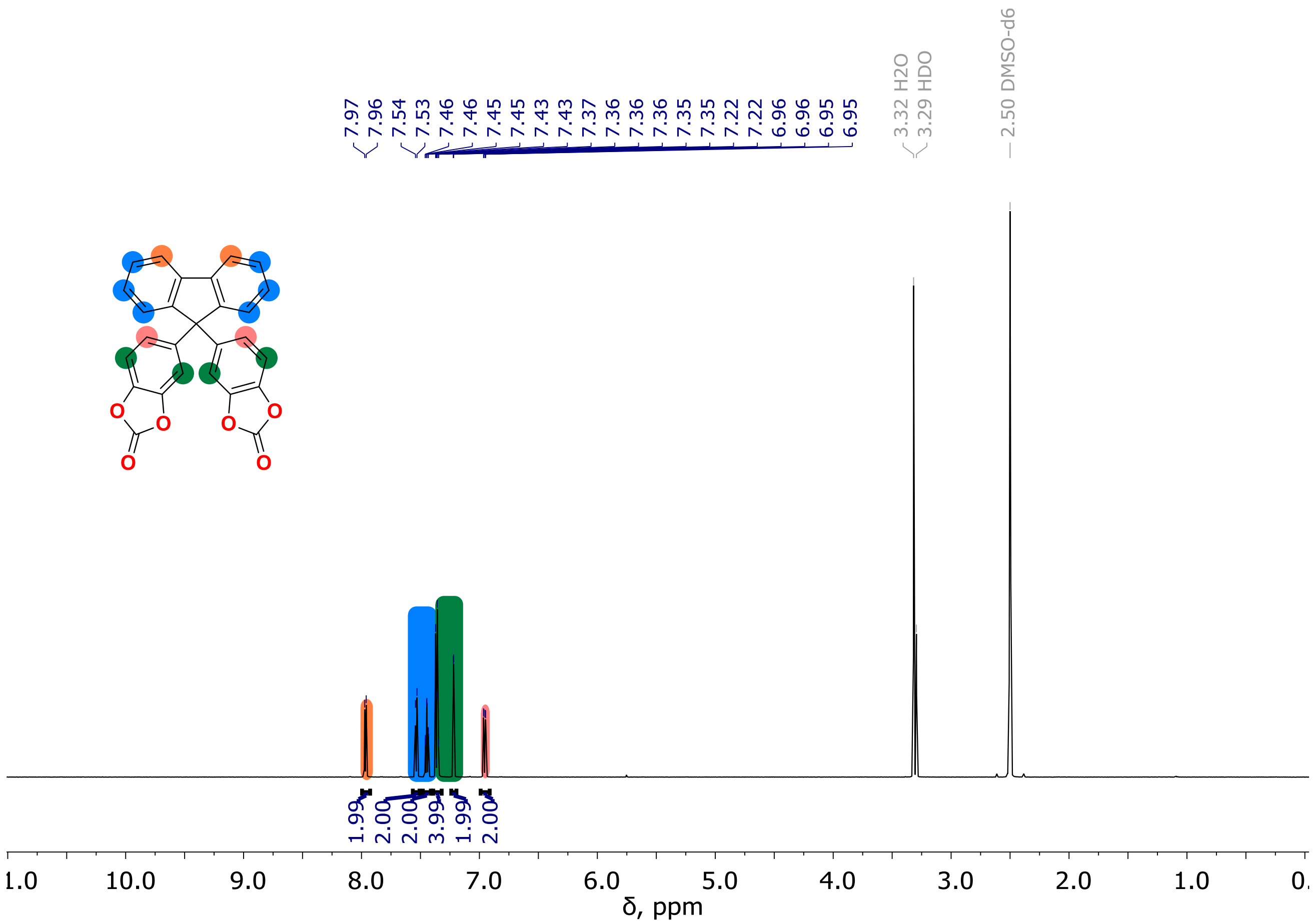

Figure S6. ^{1}H NMR spectrum of $\mathbf{M_2}$ in DMSO-d$_6$.

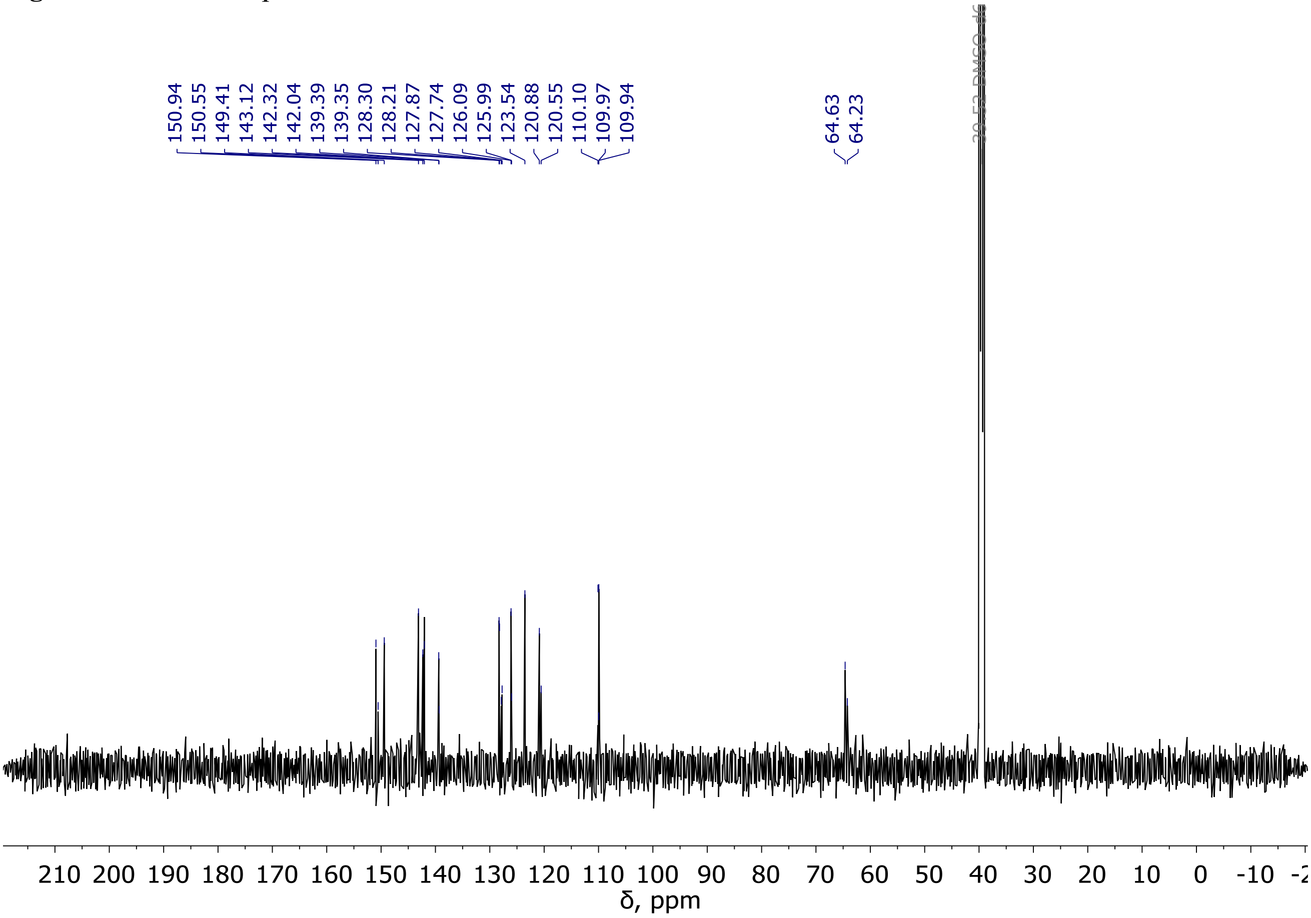

Figure S7. ^{13}C NMR spectrum of $\mathbf{M_2}$ in DMSO-d$_6$.

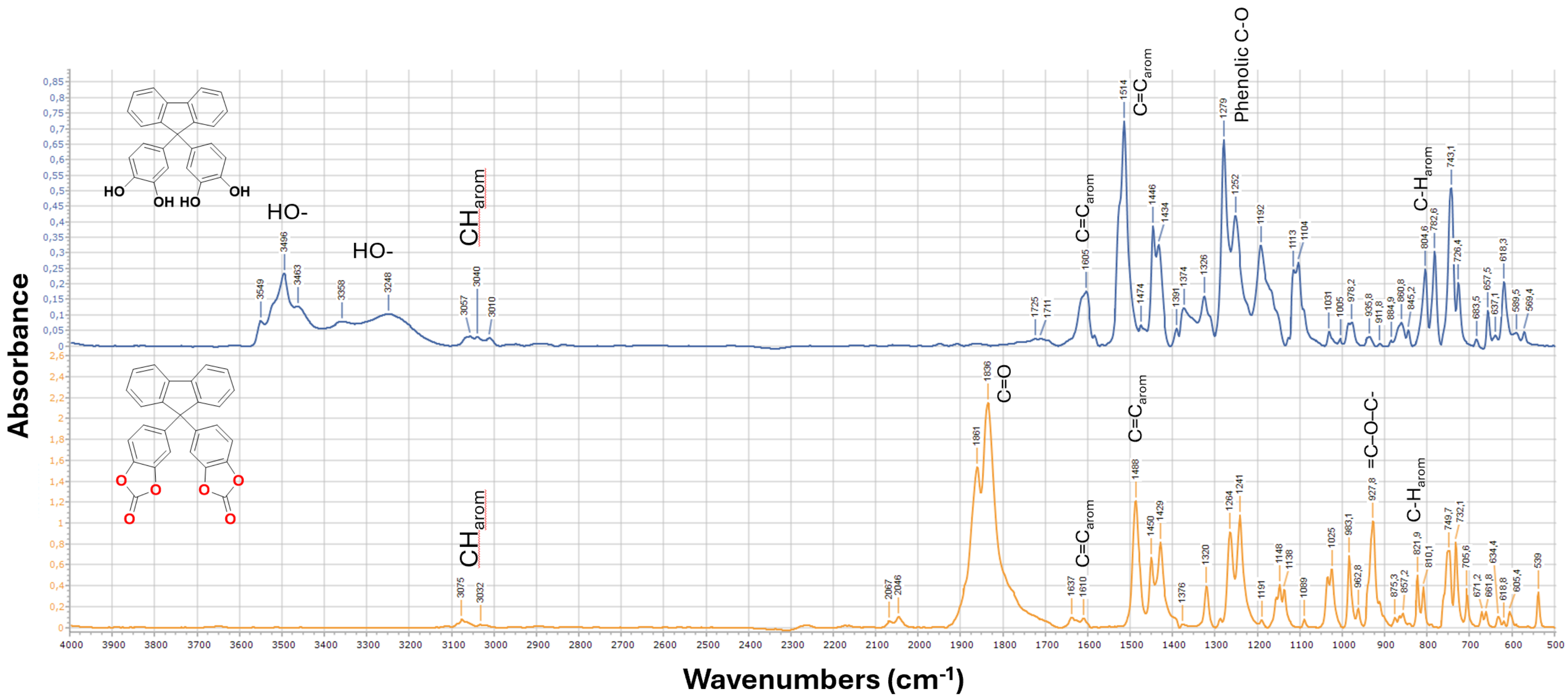

Figure S8. IR spectrum of $\mathbf{M_2}$ and its precursor.

V. Kinetic experiments with various monoamines.

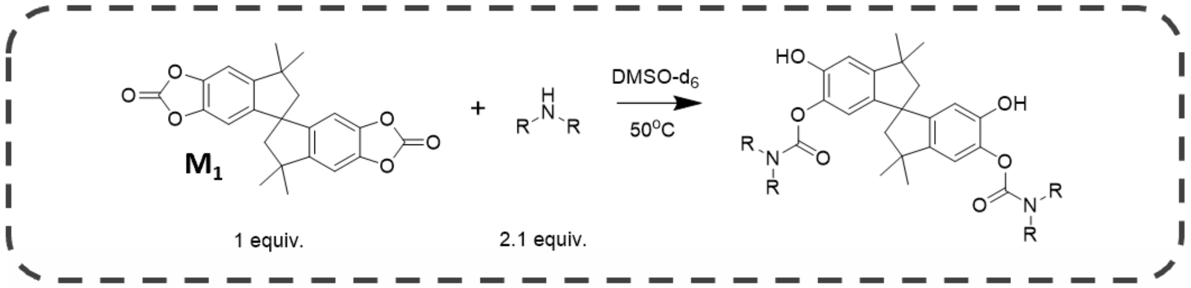

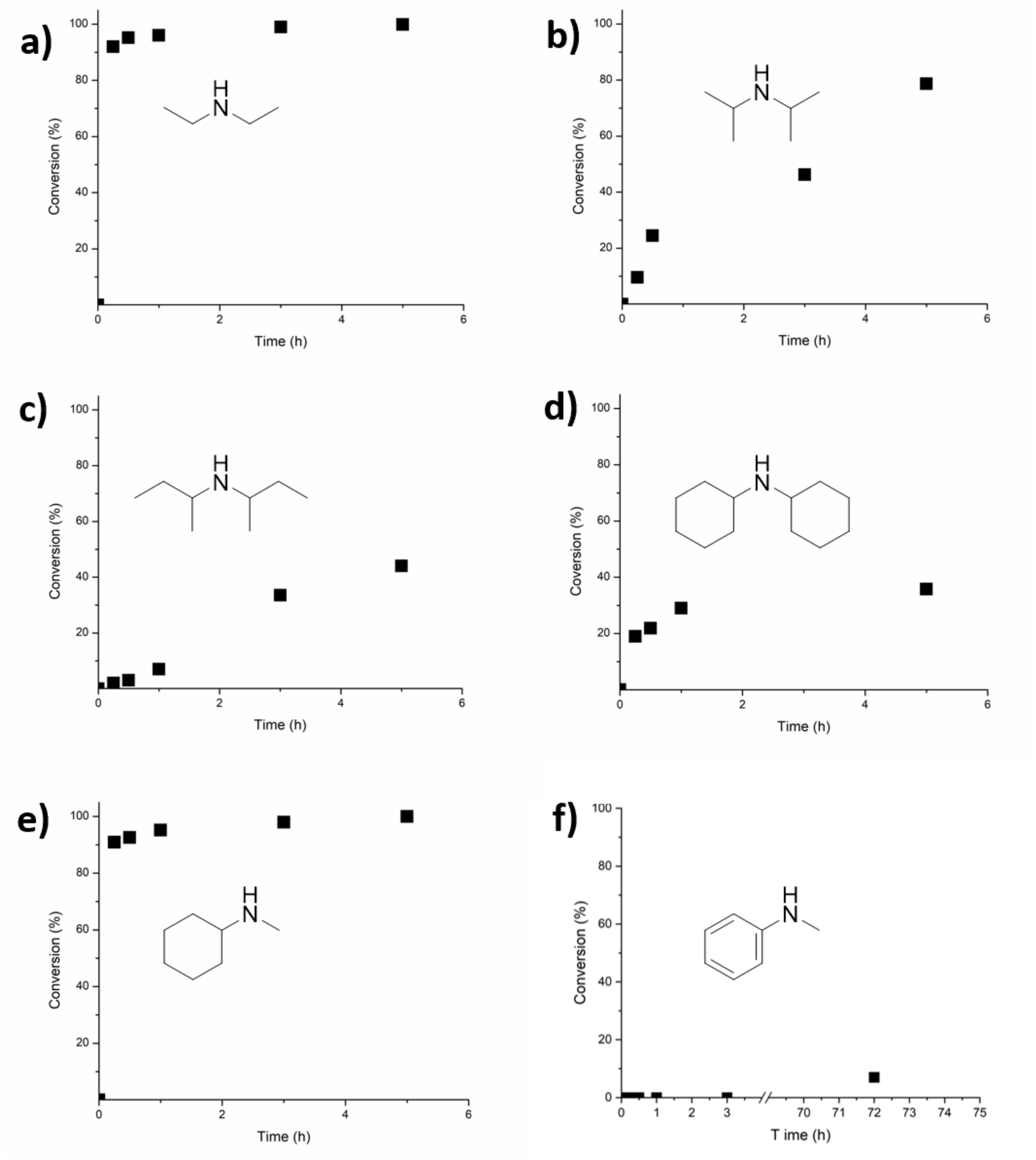

Figure S9. Model reaction of $\mathbf{M_1}$ with various secondary amines (solvent DMSO-d_6, concentration 0.2 mol/L, amine:$\mathbf{M_1}$ = 2.1:1.0, molar ratio, 50 °C). Conversion was calculated by ^{1}H NMR using the ratio of the integrations of the signals at 6.03-6.05 (two aromatic protons of reacted $\mathbf{M_1}$) to those at 7.4-7.46 (two aromatic protons of unreacted $\mathbf{M_1}$).

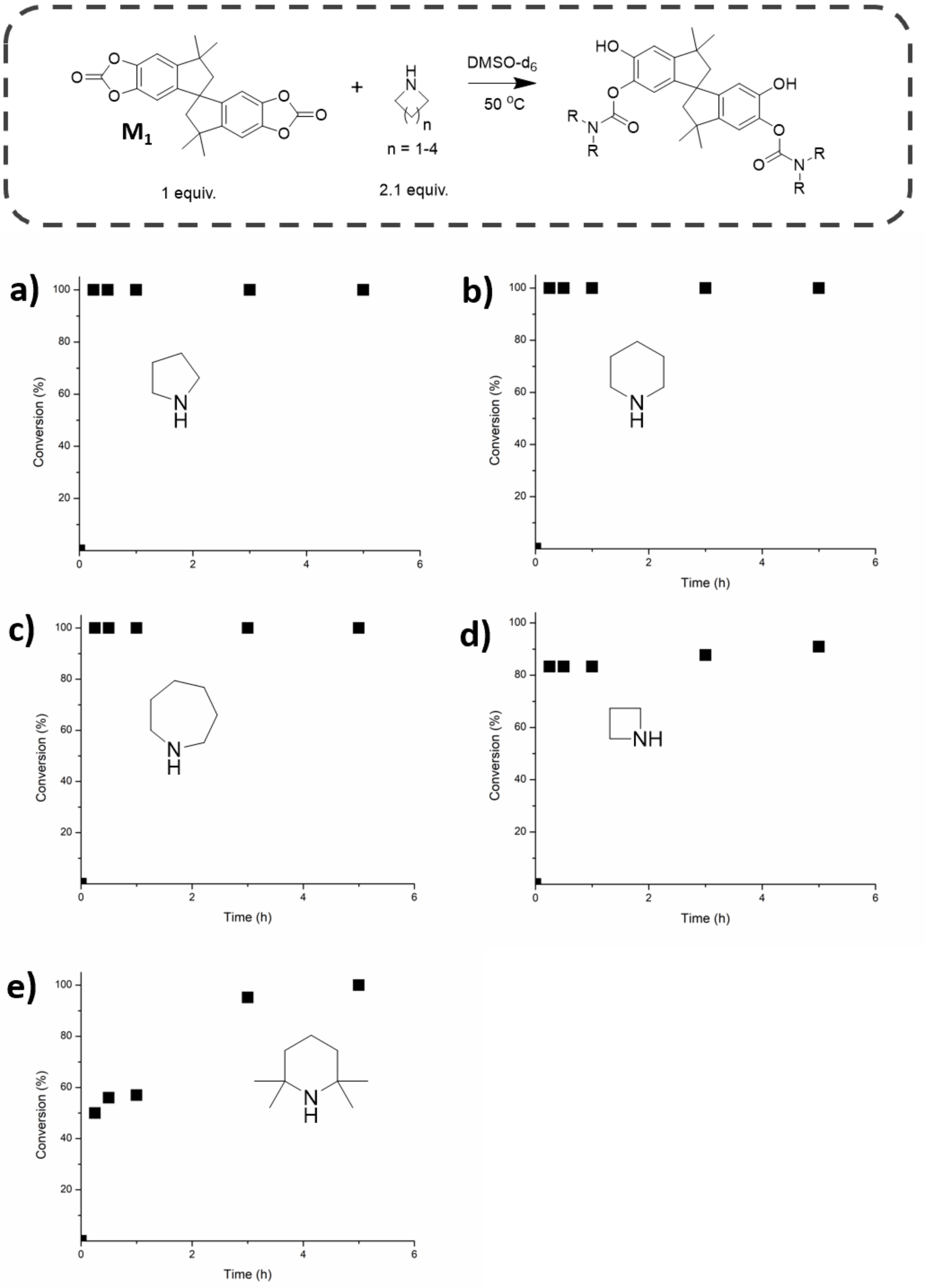

Figure S10. Model reaction of cyclic dicarbonate $\mathbf{M_1}$ with alicyclic secondary amines (solvent DMSO-d_6, concentration 0.2 mol/L, amine:$\mathbf{M_1}$ = 2.1:1.0, molar ratio, 50 °C). Conversion was calculated by ^{1}H NMR using the ratio of the integrations of signals at 6.03-6.05 (two aromatic protons of reacted $\mathbf{M_1}$) to those at 7.4-7.46 (two aromatic protons of unreacted $\mathbf{M_1}$).

V.1. Model reaction between M_1 and piperidine

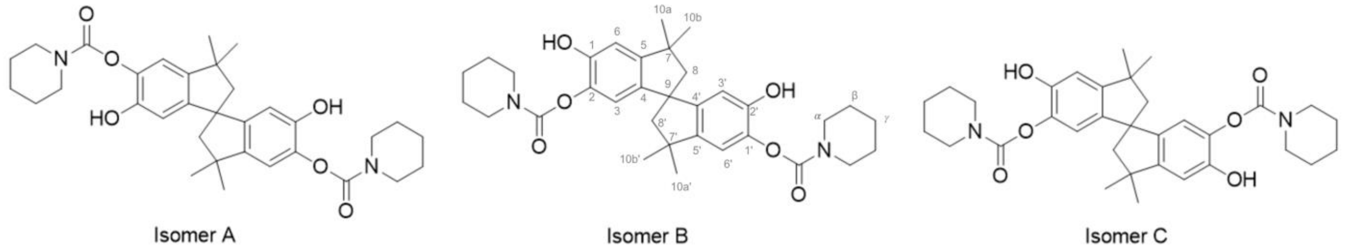

Scheme S3. Products of the model reaction between $\mathbf{M_1}$ and piperidine (Note: for isomers A and B the primed and unprimed atoms are identical).

The ^{1}H spectrum (**Figure S11**) of the isolated reaction product between $\mathbf{M_1}$ and piperidine displays two sets of aromatic proton signals: one between 6.85–6.65 ppm and another between 6.35–6.20 ppm. Each set comprises four signals, two of which exhibit identical integrals. This pattern is characteristic of isomer B, in which all four aromatic protons are magnetically non-equivalent. The signals of the low-field group show strong through-space correlations with the methyl protons H10 (**Figure S12**), and can therefore be assigned to H6 and H6′. The high-field group, which correlates with the methylene protons H8, is accordingly assigned to H3 and H3′. The NOESY spectrum reveals that, within each of the two aromatic signal groups, the two most high-field signals correlate with the resonance of the exchangeable protons (H_2O and OH, 3.3 ppm). These correlations indicate that the corresponding aromatic protons are located closest to the hydroxyl groups. This observation enables an unambiguous assignment of the aromatic signals (**Figure S11**).

The piperidyl-α protons appear as two distinct signals at 3.53 ppm and 3.47 ppm. The latter shows cross-peaks with the aromatic resonances at 6.32 ppm and 6.28 ppm (H3B and H3C) and is therefore assigned to piperidine units attached at position 2, while the former corresponds to those attached at position 1 (**Figure S12**).

Additionally, through-space correlations between methylene and methyl protons indicate spatial proximity between H7a–H10a and H7b–H10b (**Figure S13**).

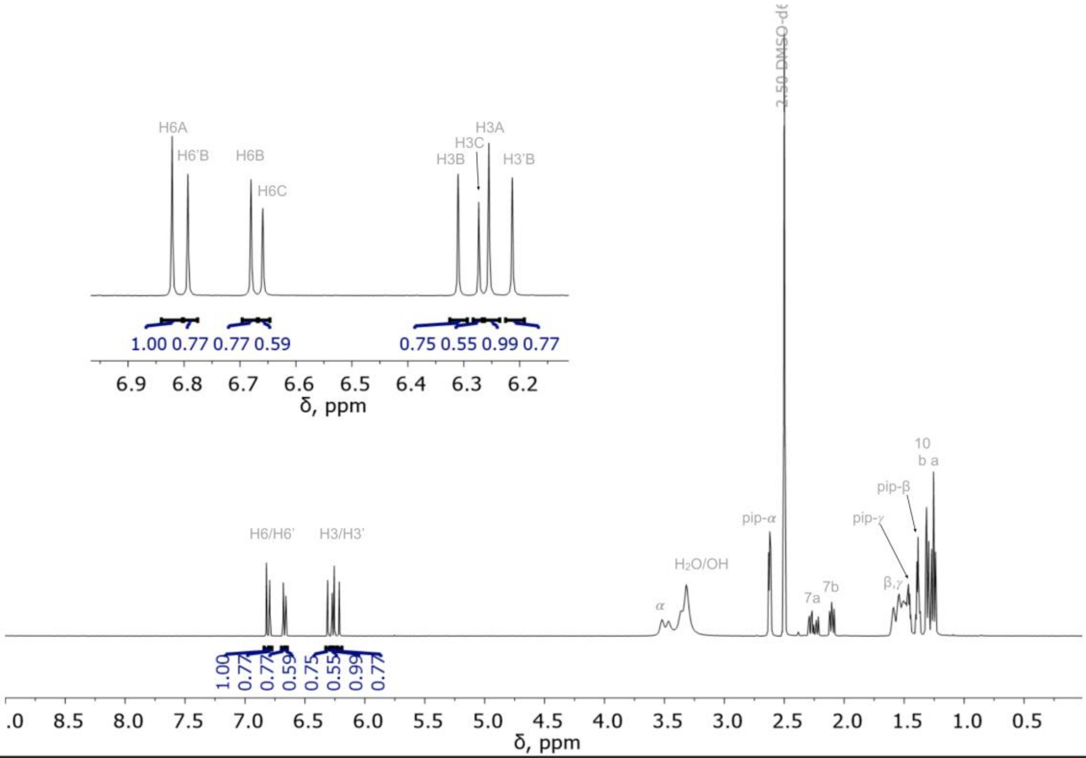

Figure S11. ^{1}H NMR (DMSO-d_6) spectrum of 3 isomers obtained by model reaction between $\mathbf{M_1}$ and piperidine.

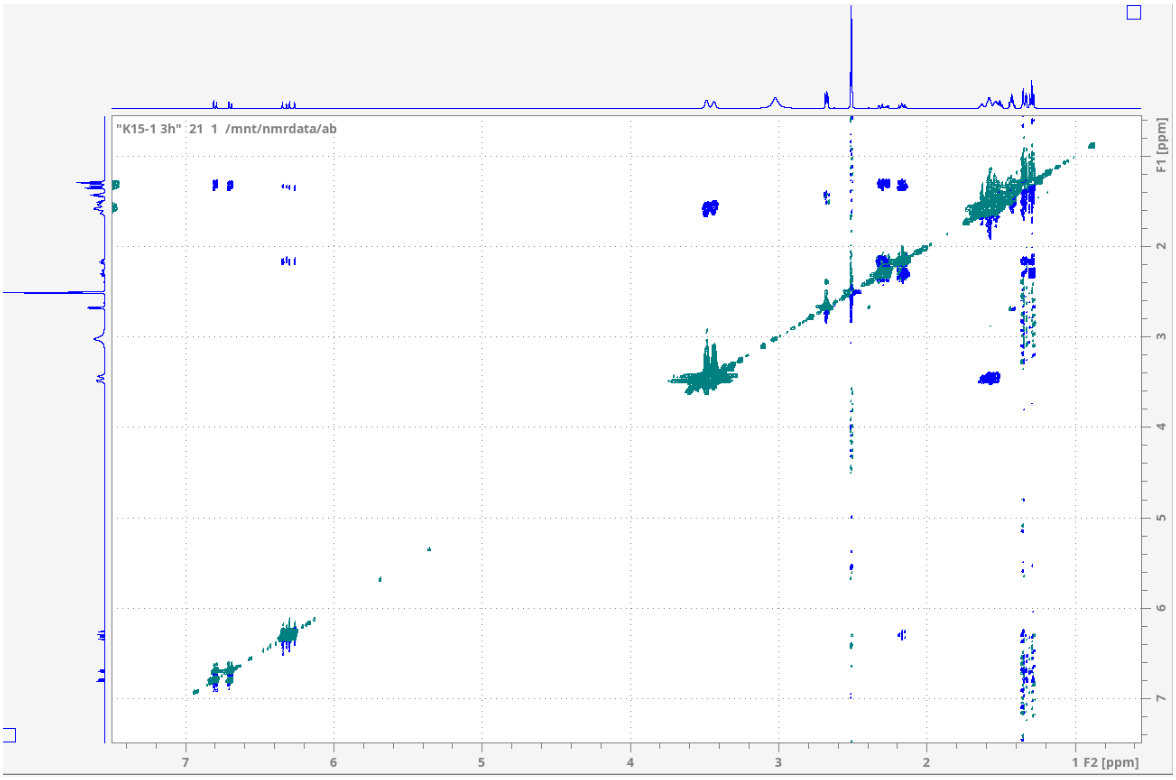

Figure S12. ROESY NMR (DMSO-d_6) spectrum at 360K (87°C) of 3 isomers obtained by model reaction between $\mathbf{M_1}$ and piperidine.

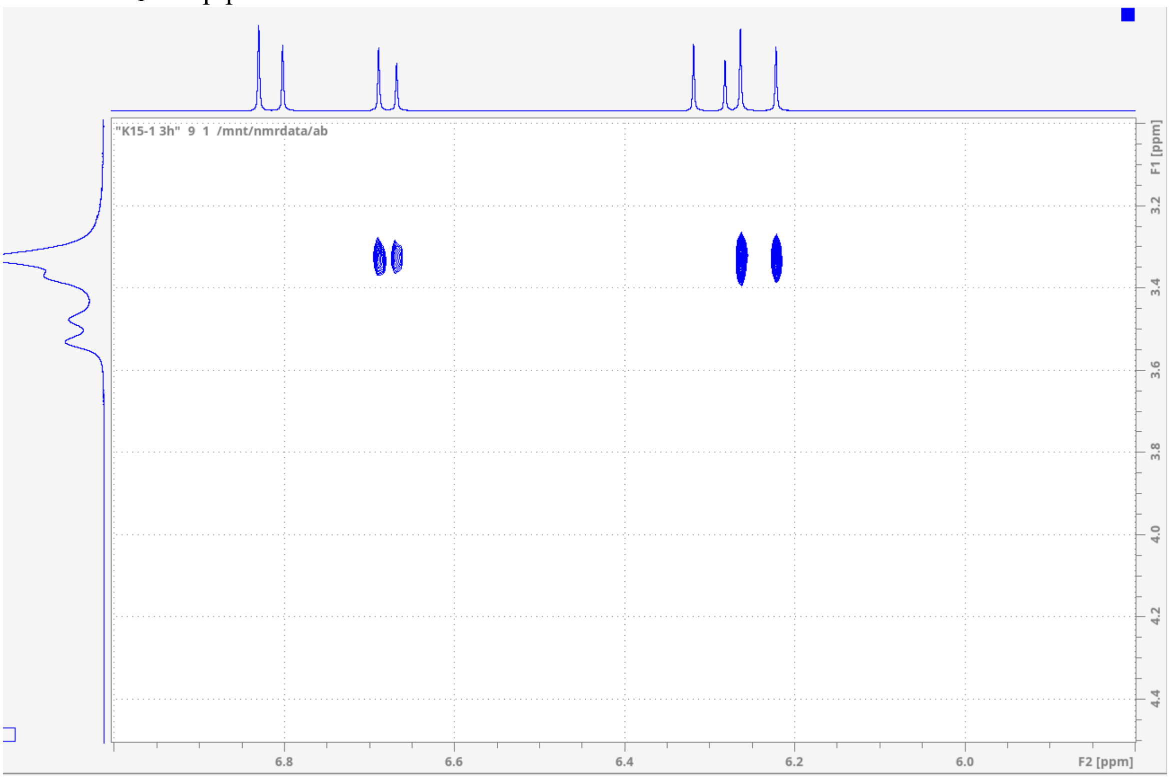

Figure S13. NOESY NMR (DMSO-d_6) spectrum at 300K (27°C) of 3 isomers obtained by model reaction between $\mathbf{M_1}$ and piperidine.

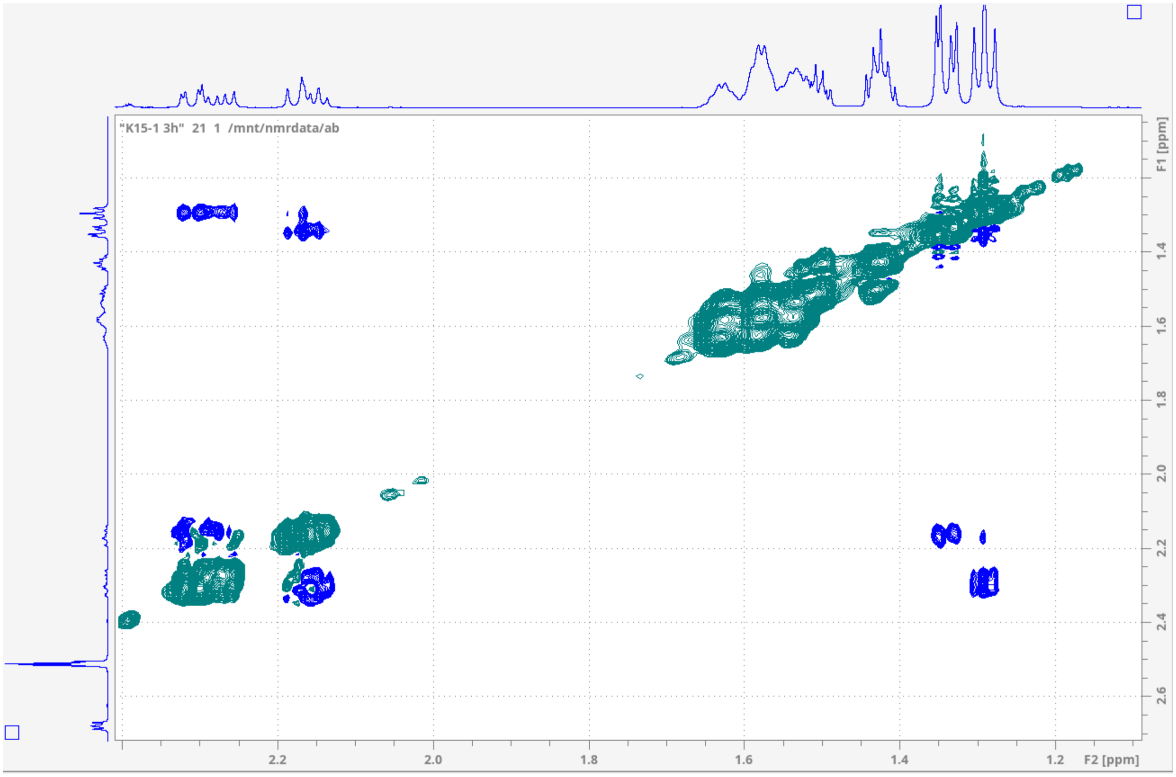

Figure S14. High-field region of the ROESY NMR (DMSO-d_6) spectrum at 360K (87°C) of 3 isomers obtained by model reaction between $\mathbf{M_1}$ and piperidine.

VI. Synthesis of PHUs.

VI.1. General method.

All PHUs were synthesized via polyaddition of bis(cyclic carbonate)s (BCCs) and diamines following general procedure described below:

Polyaddition was carried out in a conical three-neck flask equipped with a mechanical stirrer. BCC (3 mmol) and diamine (3 mmol) were introduced into the flask under an argon atmosphere, followed by the addition of 2 mL of anhydrous DMSO. The reaction mixture was heated to 50 °C in an oil bath and stirred at this temperature for 3 h. Afterwards, the heating was stopped, and the resulting highly viscous solution was diluted with 40 mL of THF. The polymer was then isolated by precipitation into excess of isopropanol, collected by filtration, thoroughly washed with 10-15 mL of methanol, and dried under vacuum (<0.5 mbar) at 100 °C overnight.

VI.2. Synthesis of PHU based on $\mathbf{M_1}$ and piperazine ($\mathbf{PHU_1}$).

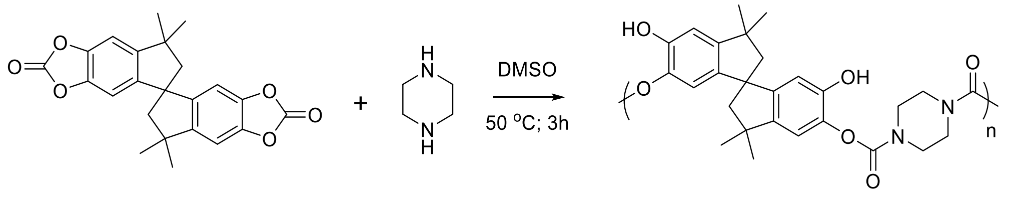

Scheme S4. Synthesis of $\mathbf{PHU_1}$ polymer.

The following loading was applied: $\mathbf{M_1}$ (1.175 g, 3 mmol) and piperazine (0.258 g, 3 mmol). Yield: 1.38 g (95 %). M_n (GPC) = 105400 g/mol, M_w/M_n = 3.6. DP_n = 220. M_{sD} (ultracentrifugation) = 71200 g/mol. T_{onset} (TGA, 5°C/min, on air) = 220°C; T_{onset} (TGA, 5°C/min, N_2) = 225°C. T_g (TMA in He, 5°C/min) = 253°C.

^{1}H NMR (600 MHz, DMSO-d_6) δ 9.44 – 9.11 (m, 2H), 6.93 – 6.61 (m, 2H), 6.42 – 6.17 (m, 2H), 3.53 (d, J = 85.5 Hz, 8H), 2.20 (d, J = 102.0 Hz, 4H), 1.43 – 1.11 (m, 12H).

^{13}C NMR (151 MHz, DMSO-d_6) δ 153.04, 152.92, 149.41, 149.25, 148.69, 148.56, 148.45, 147.87, 147.57, 142.22, 142.08, 140.40, 140.03, 138.61, 138.52, 118.10, 116.32, 111.11, 109.47, 59.26, 56.85, 56.47, 56.06, 43.99, 43.27, 42.89, 42.68, 42.44, 31.44, 31.32, 31.28, 30.40, 30.36, 30.15, 30.12.

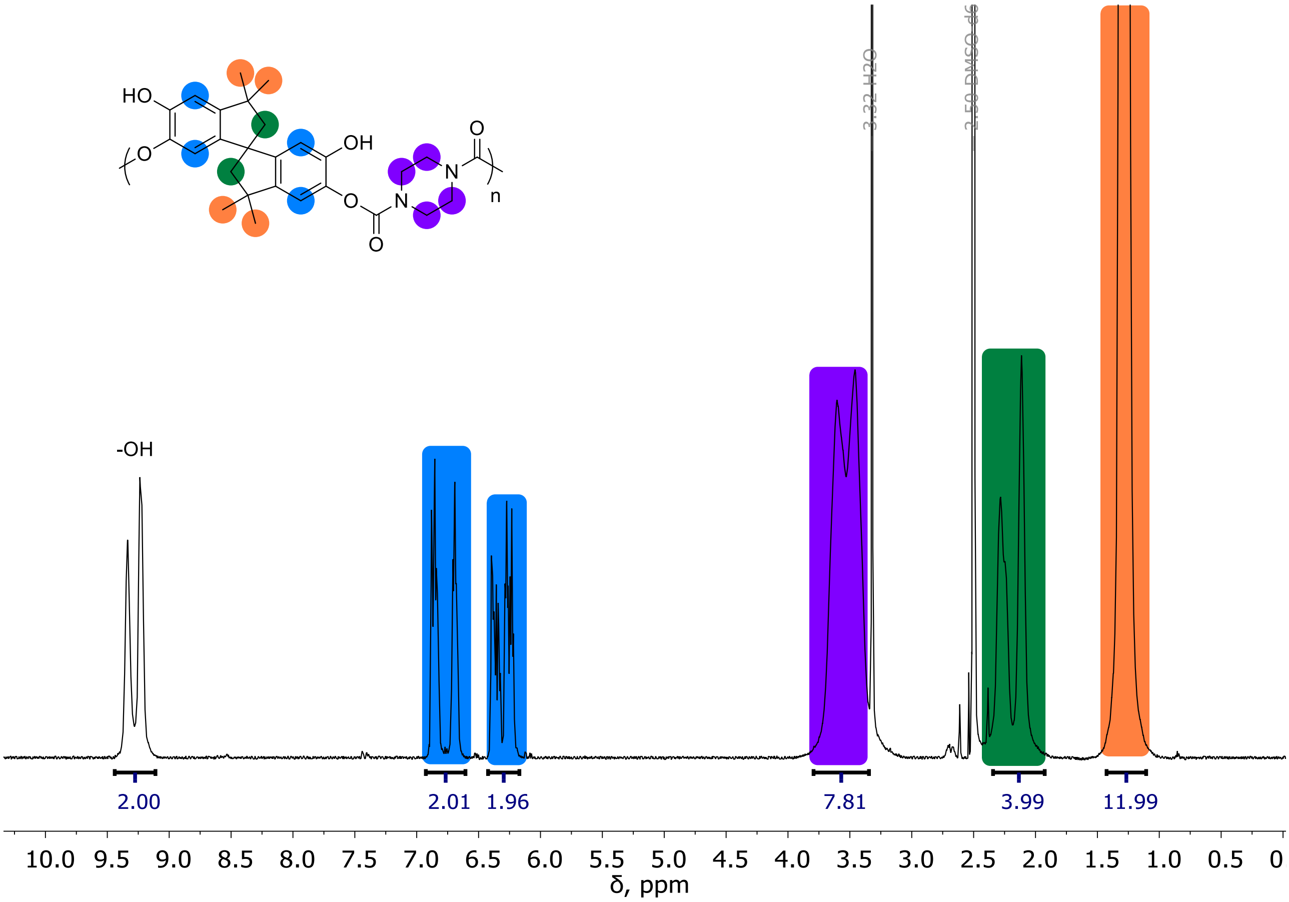

Figure S15. ^{1}H NMR spectrum of **PHU$_1$** in DMSO-d$_6$.

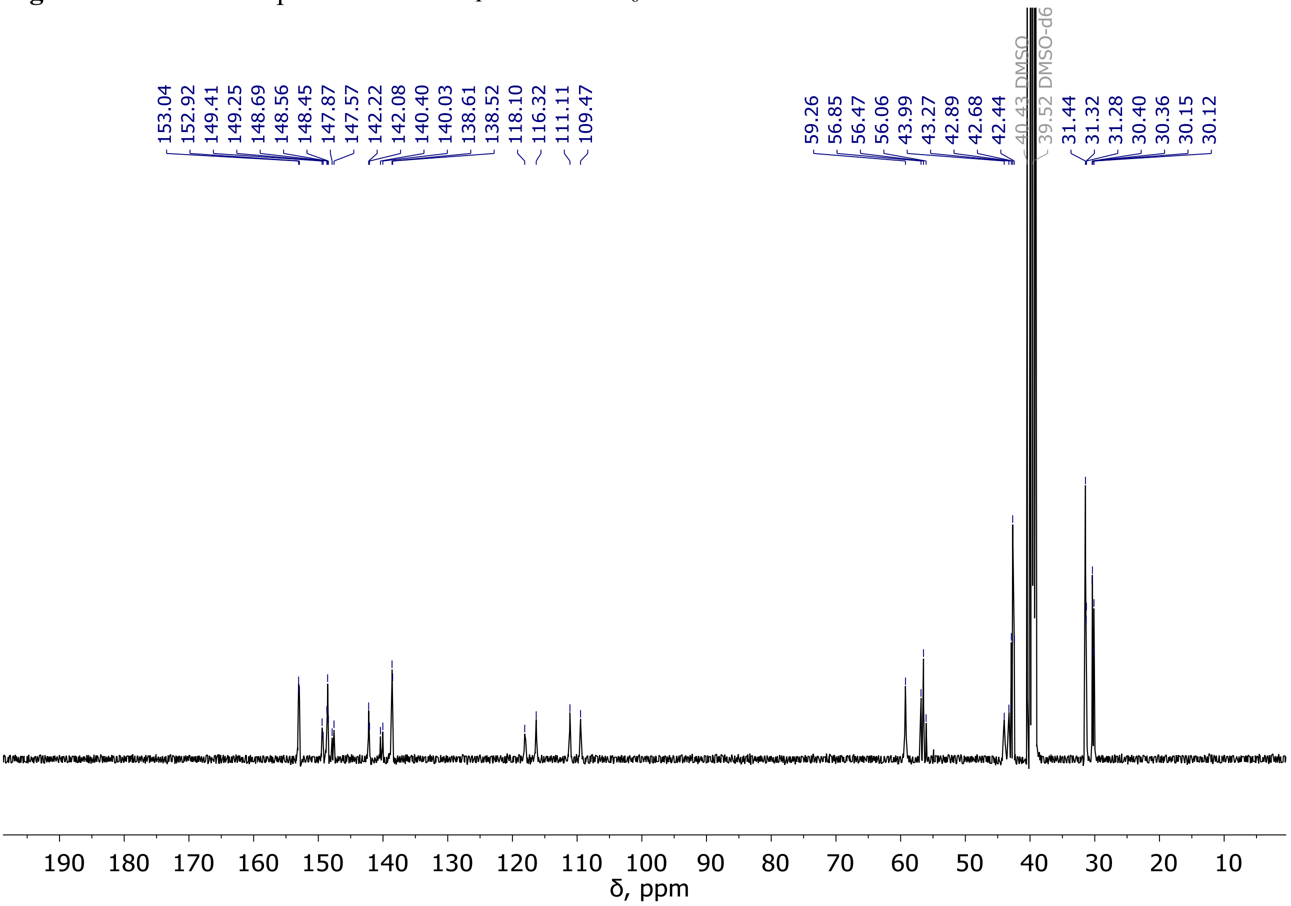

Figure S16. ^{13}C NMR spectrum of **PHU$_1$** in DMSO-d$_6$.

VI.3. Synthesis of PHU based on $\mathbf{M_1}$ and homopiperazine ($\mathbf{PHU_2}$).

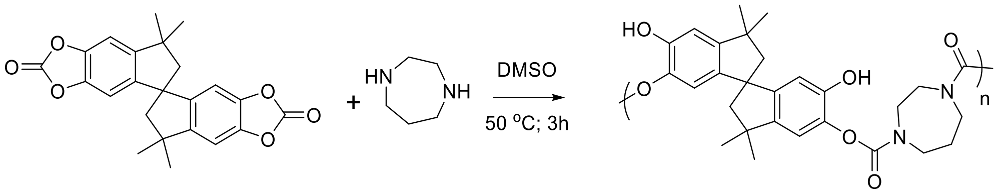

Scheme S5. Synthesis of $\mathbf{PHU_2}$ polymer.

The following loading was applied: $\mathbf{M_1}$ (1.175 g, 3 mmol) and homopiperazine (0.3 g, 3 mmol). Yield: 1.29 g (87 %). M_n (GPC) = 95300 g/mol, M_w/M_n = 2.8, DP_n = 194.
T_{onset} (TGA, 5°C/min, on air) = 200°C; T_{onset} (TGA, 5°C/min, N_2) = 200°C. T_g (DSC, 5°C/min, sealed under N_2) = 225°C. T_g (TMA in He, 5°C/min) = 263°C.
^{1}H NMR (600 MHz, DMSO-d_6) δ 9.60 – 9.10 (m, 2H), 6.97 – 6.56 (m, 2H), 6.49 – 6.14 (m, 2H), 3.75 – 3.38 (m, 8H), 2.40 – 1.75 (m, 6H), 1.40 – 1.19 (m, 12H).
^{13}C NMR (151 MHz, DMSO-d_6) δ 153.61, 153.37, 149.26, 149.07, 148.60, 148.43, 147.75, 147.43, 142.18, 142.01, 140.31, 139.92, 138.94, 138.78, 118.03, 116.35, 111.05, 109.34, 59.26, 56.84, 56.49, 42.85, 42.66, 42.41, 31.47, 31.44, 31.33, 31.28, 30.43, 30.36, 30.08.

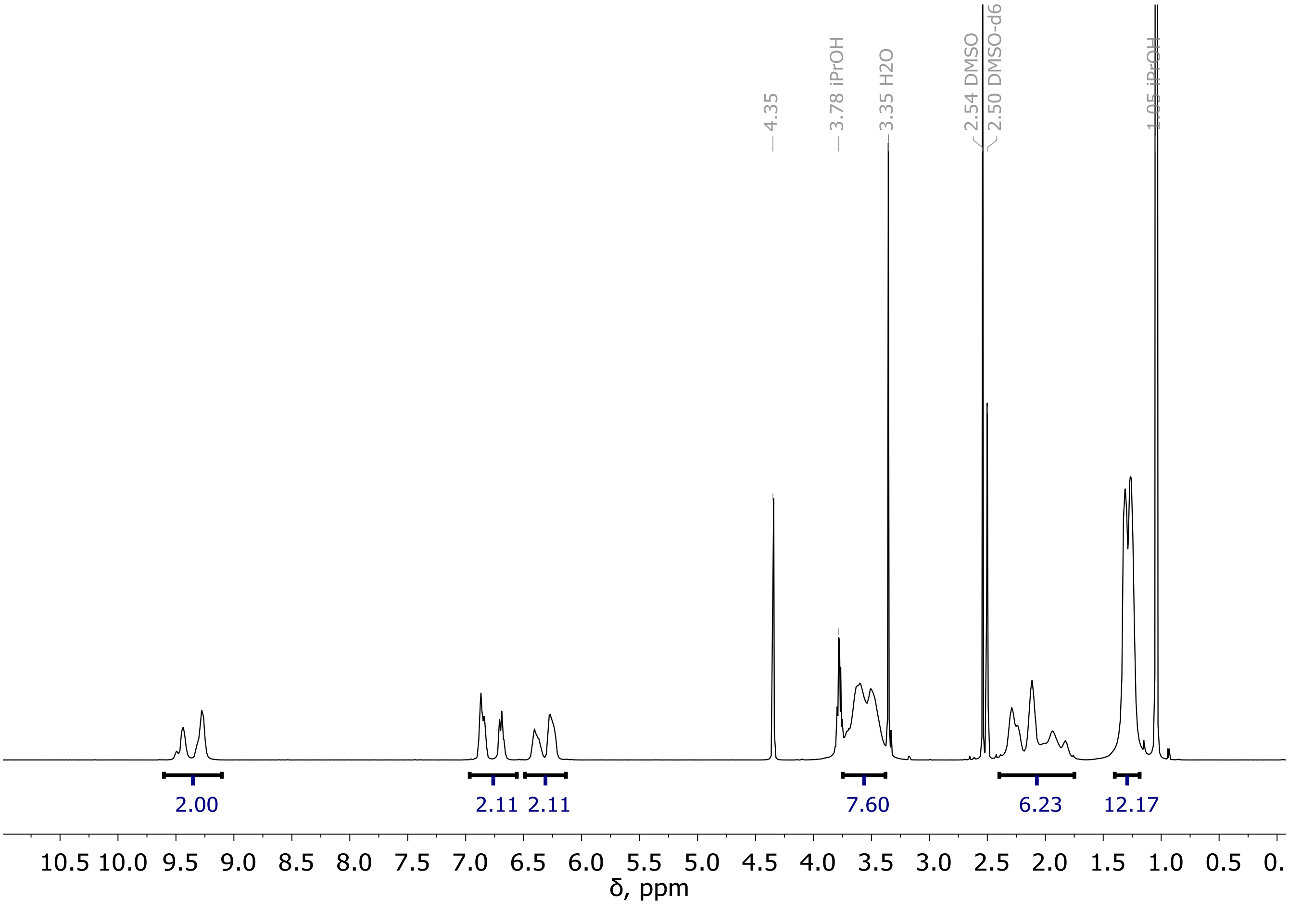

Figure S17. ^{1}H NMR spectrum of $\mathbf{PHU_2}$ in DMSO-d_6.

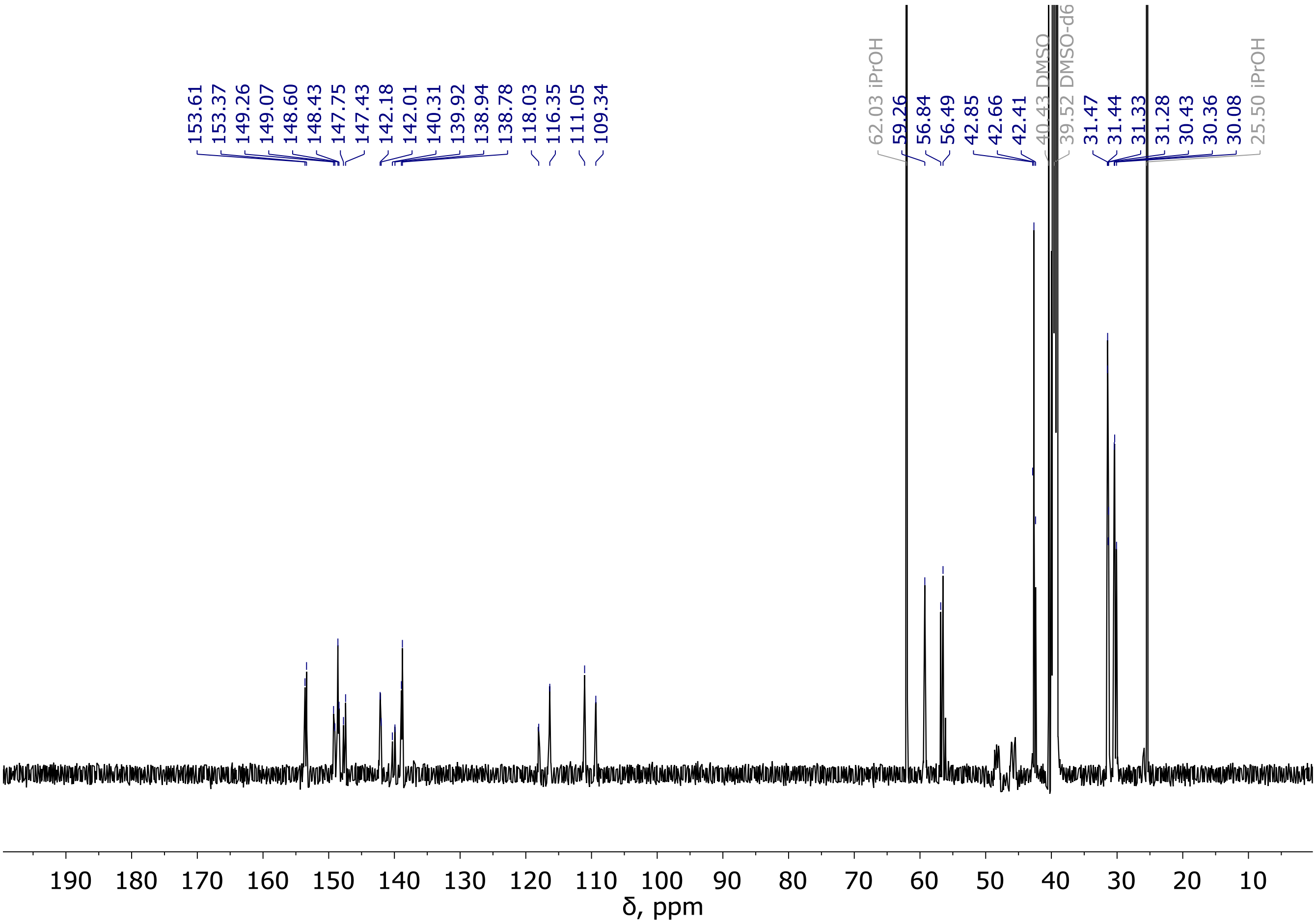

Figure S18. ^{13}C NMR spectrum of **PHU₂** in DMSO-d₆.

VI.4. Synthesis of PHU based on $\mathbf{M_1}$ and 1,3-di(piperidin-4-yl)propane ($\mathbf{PHU_3}$).

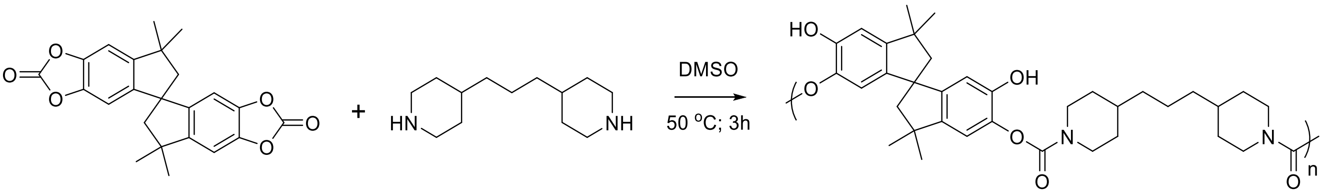

Scheme S6. Synthesis of $\mathbf{PHU_3}$ polymer.

The following loading was applied: $\mathbf{M_1}$ (1.175 g, 3 mmol) and 1,3-di(piperidin-4-yl)propane (0.630 g, 3 mmol). Yield: 1.44 g (80 %). M_n (GPC) = 94400 g/mol, M_w/M_n = 3.8, DP_n = 157. M_{sD} (ultracentrifugation) = 82500 g/mol. T_{onset} (TGA, 5°C/min, on air) = 265°C; T_{onset} (TGA, 5°C/min, N_2) = 270°C. T_g (DSC, 5°C/min, sealed under N_2) = 196°C. T_g (TMA in He, 5°C/min) = 226°C.

^{1}H NMR (600 MHz, DMSO-d$_6$) δ 9.39 – 9.05 (m, 2H), 6.72 (dd, J = 75.1, 15.2 Hz, 2H), 6.25 (dd, J = 30.8, 21.1 Hz, 2H), 4.24 – 3.83 (m, 4H), 2.82 (d, J = 87.9 Hz, 4H), 2.35 – 1.96 (m, 4H), 1.75 – 1.52 (m, 4H), 1.35 – 1.16 (m, 16H).

^{13}C NMR (151 MHz, DMSO-d$_6$) δ 152.90, 152.79, 149.06, 148.91, 148.72, 148.63, 148.50, 147.50, 147.29, 142.06, 141.92, 139.97, 138.85, 138.74, 116.29, 110.98, 109.30, 59.26, 56.78, 56.42, 56.02, 44.46, 43.99, 42.82, 42.63, 42.38, 36.08, 35.02, 31.41, 31.29, 30.38, 30.34, 30.10, 22.99.

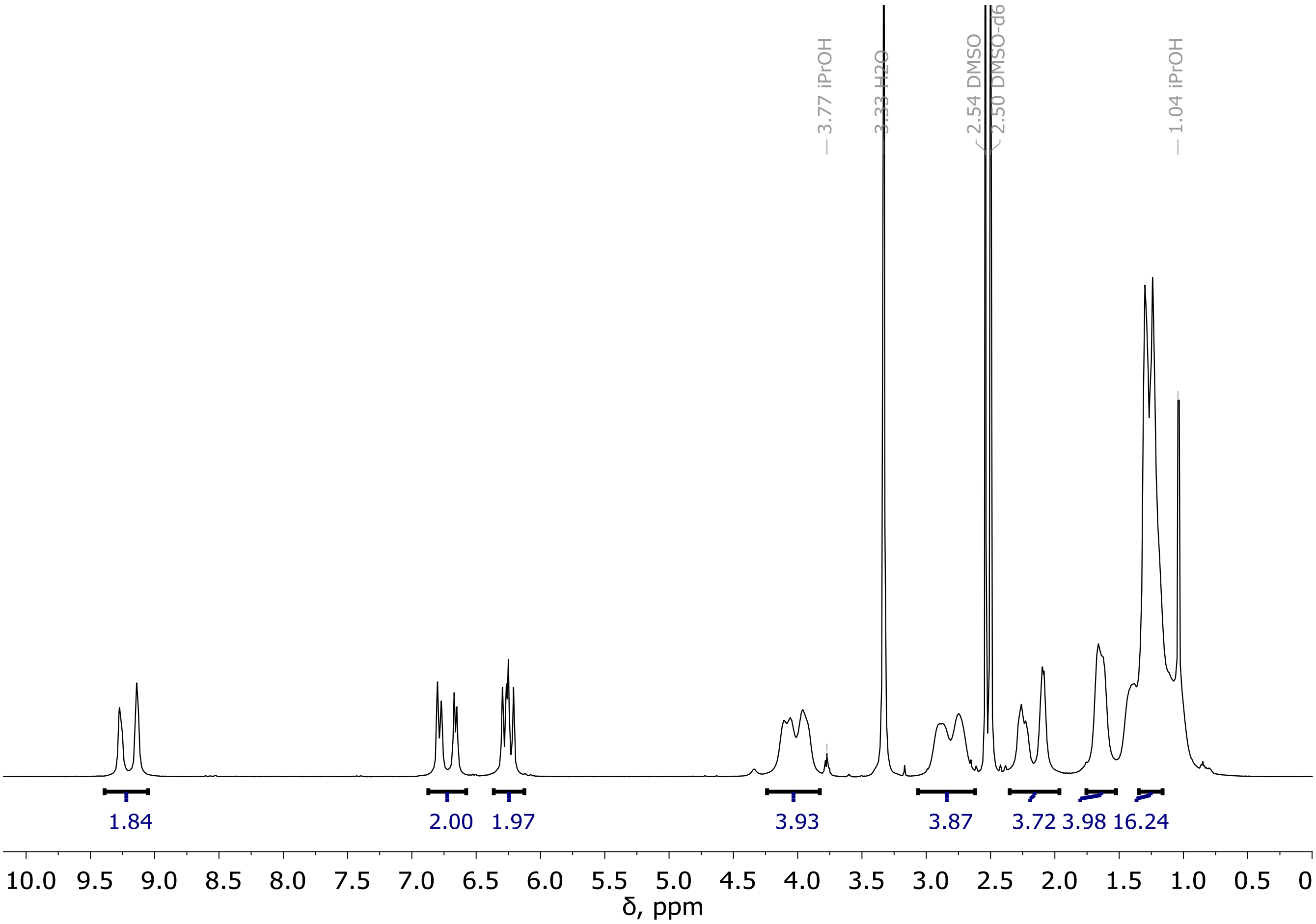

Figure S19. ^1H NMR spectrum of **PHU$_3$** in DMSO-d_6.

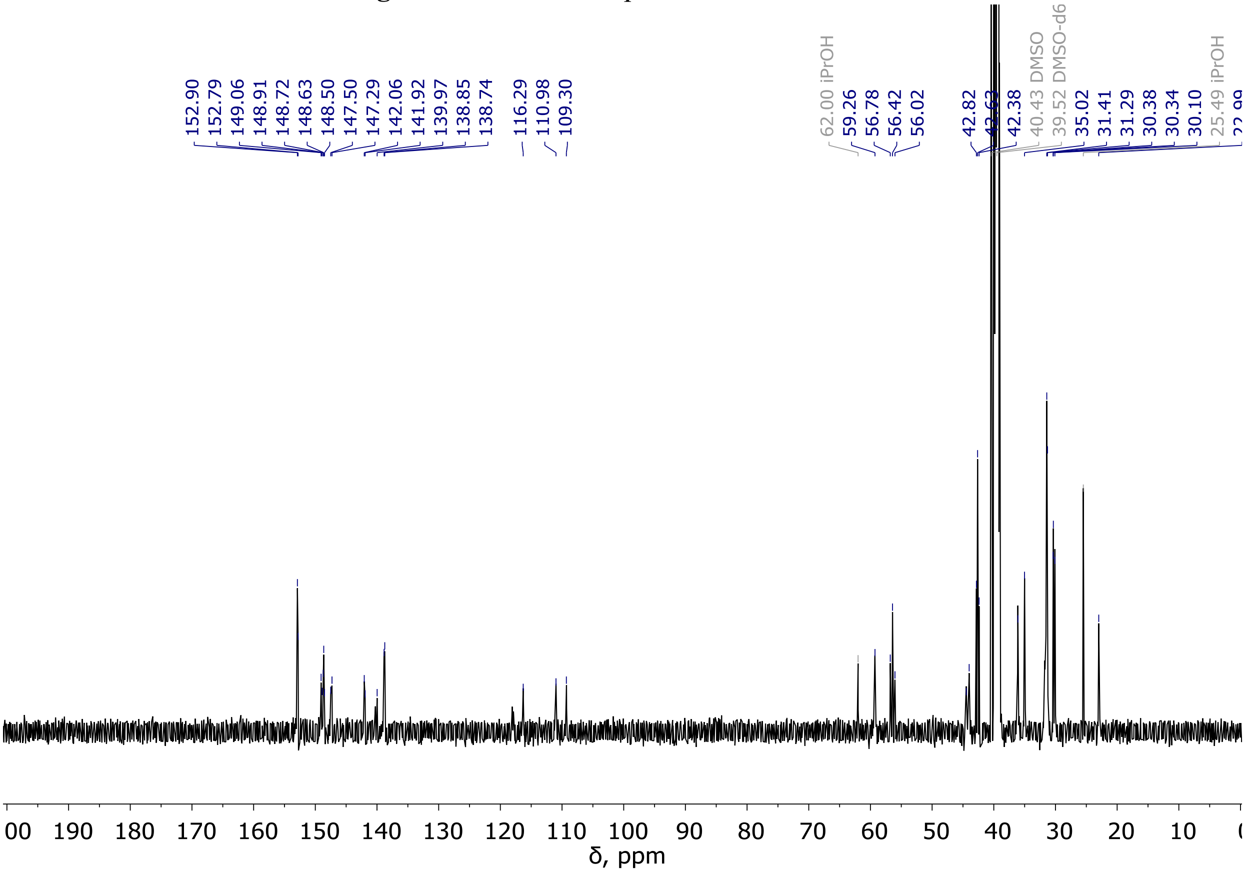

Figure S20. ^{13}C NMR spectrum of **PHU$_3$** in DMSO-d_6.

VI.5. Synthesis of PHU based on **M1** and octahydro-1H-pyrrolo[3,4-b]pyridine (**PHU4**).

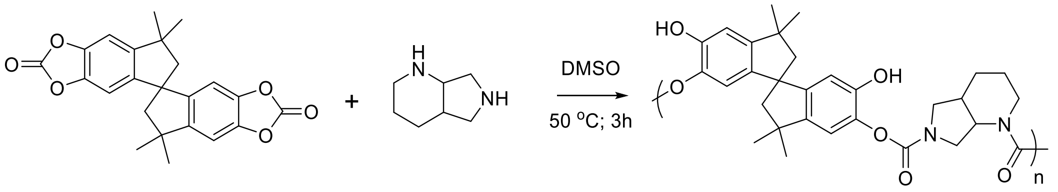

Scheme S7. Synthesis of **PHU4** polymer.

The following loading was applied: **M1** (1.175 g, 3 mmol) and octahydro-1H-pyrrolo[3,4-b]pyridine (0.378 g, 3 mmol). Yield: 1.28 g (82 %). M_n (GPC) = 29100 g/mol, M_w/M_n = 1.93, DP_n = 56.

T_{onset} (TGA, 5°C/min, on air) = 235°C; T_{onset} (TGA, 5°C/min, N_2) = 295°C. T_g (DSC, 5°C/min, sealed under N_2) = 257°C, T_g (TMA in He, 5°C/min) = 262°C.

^{1}H NMR (600 MHz, DMSO-d$_6$) δ 9.47 – 9.03 (m, 2H), 6.76 (dd, *J* = 98.5, 14.9 Hz, 2H), 6.43 – 6.14 (m, 2H), 5.01 – 4.50 (m, 1H), 4.21 – 3.39 (m, 3H), 3.33 – 2.79 (m, 2H), 2.40 – 1.96 (m, 5H), 1.86 – 1.42 (m, 3H), 1.42 – 1.15 (m, 12H).

^{13}C NMR (151 MHz, DMSO-d$_6$) δ 153.46, 152.87, 152.75, 149.28, 148.66, 148.55, 147.76, 147.49, 142.19, 142.05, 140.38, 140.06, 138.57, 118.04, 116.35, 111.06, 109.42, 59.26, 56.83, 56.44, 56.03, 50.91, 48.49, 42.87, 42.67, 42.64, 42.41, 35.12, 34.34, 31.43, 31.30, 30.36, 30.11, 29.01, 24.62, 23.59, 17.24.

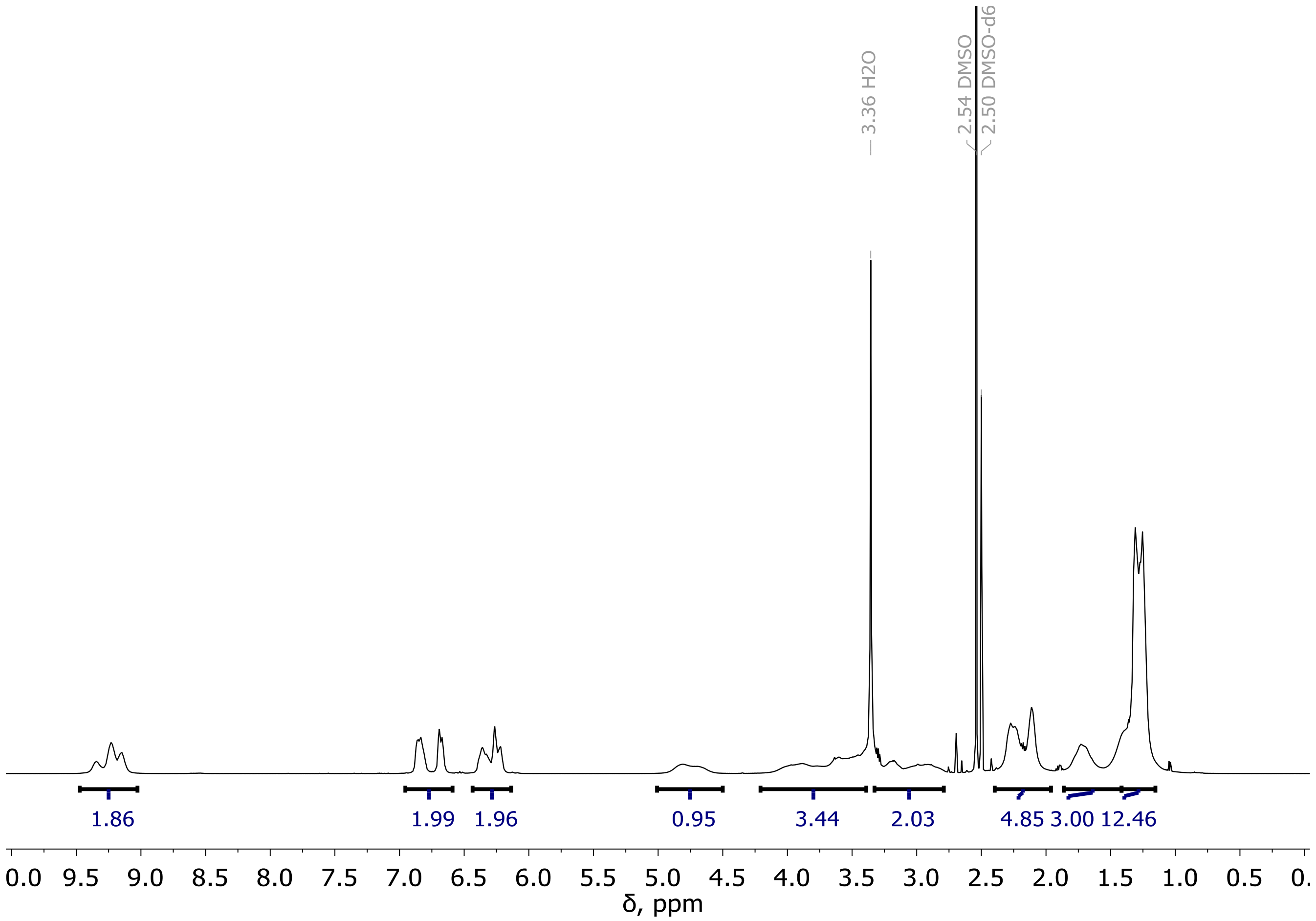

Figure S21. ^{1}H NMR spectrum of **PHU4** in DMSO-d$_6$.

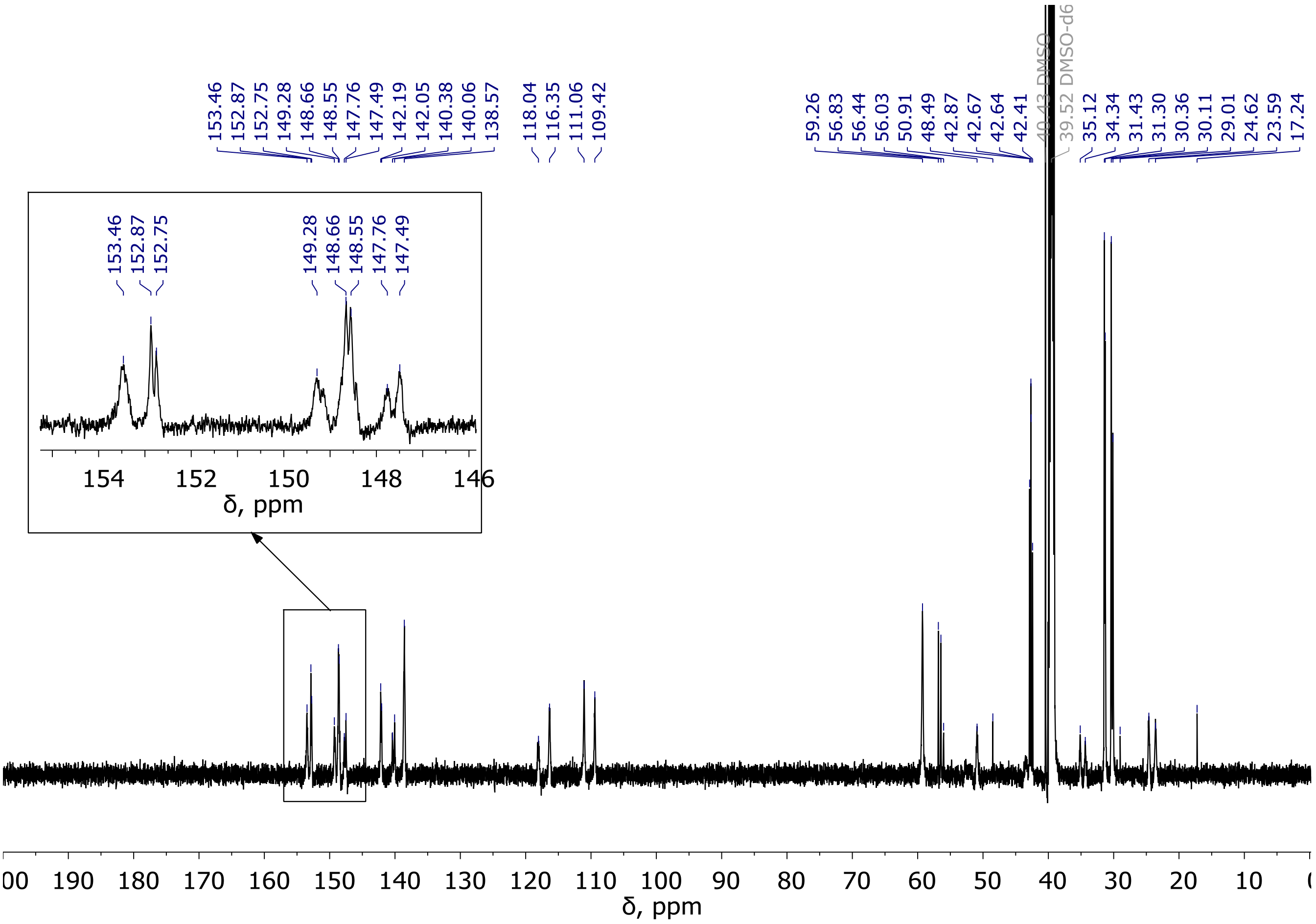

Figure S22. ^{13}C NMR spectrum of **PHU$_4$** in DMSO-d$_6$.

VI.6. Synthesis of PHU based on **M$_1$** and 3,3'-bipiperidine (**PHU$_5$**).

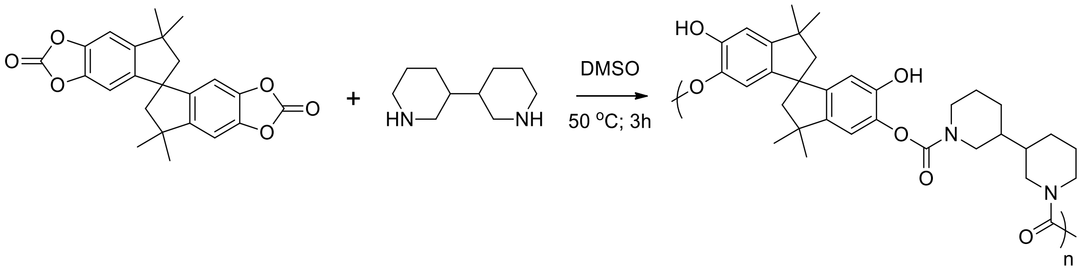

Scheme S8. Synthesis of **PHU$_5$** polymer.

The following loading was applied: **M$_1$** (1.175 g, 3 mmol) and 3,3'-bipiperidine (0.504 g, 3 mmol). Yield: 1.09 g (65 %). Yield: 1.09 g (65 %). M_n (GPC) = 41500 g/mol, M_w/M_n = 3.1, DP_n = 74. T_{onset} (TGA, 5°C/min, on air) = 230°C. T_g (DSC, 5°C/min, sealed under N_2) = 216°C, T_g (TMA in He, 5°C/min) = 242°C.

^{1}H NMR (600 MHz, DMSO-d$_6$) δ 9.21 (d, J = 83.4 Hz, 2H), 6.72 (dd, J = 79.6, 16.4 Hz, 2H), 6.26 (dd, J = 42.4, 20.1 Hz, 2H), 4.18 – 3.65 (m, 4H), 3.07 – 2.57 (m, 4H), 2.34 – 1.98 (m, 4H), 1.96 – 1.52 (m, 4H), 1.37 – 1.10 (m, 16H).

^{13}C NMR (151 MHz, DMSO-d$_6$) δ 152.92, 148.64, 147.54, 142.04, 139.93, 138.81, 116.25, 111.00, 109.30, 59.26, 56.77, 56.41, 42.79, 42.60, 42.35, 31.39, 31.26, 31.23, 30.39, 30.33, 30.07.

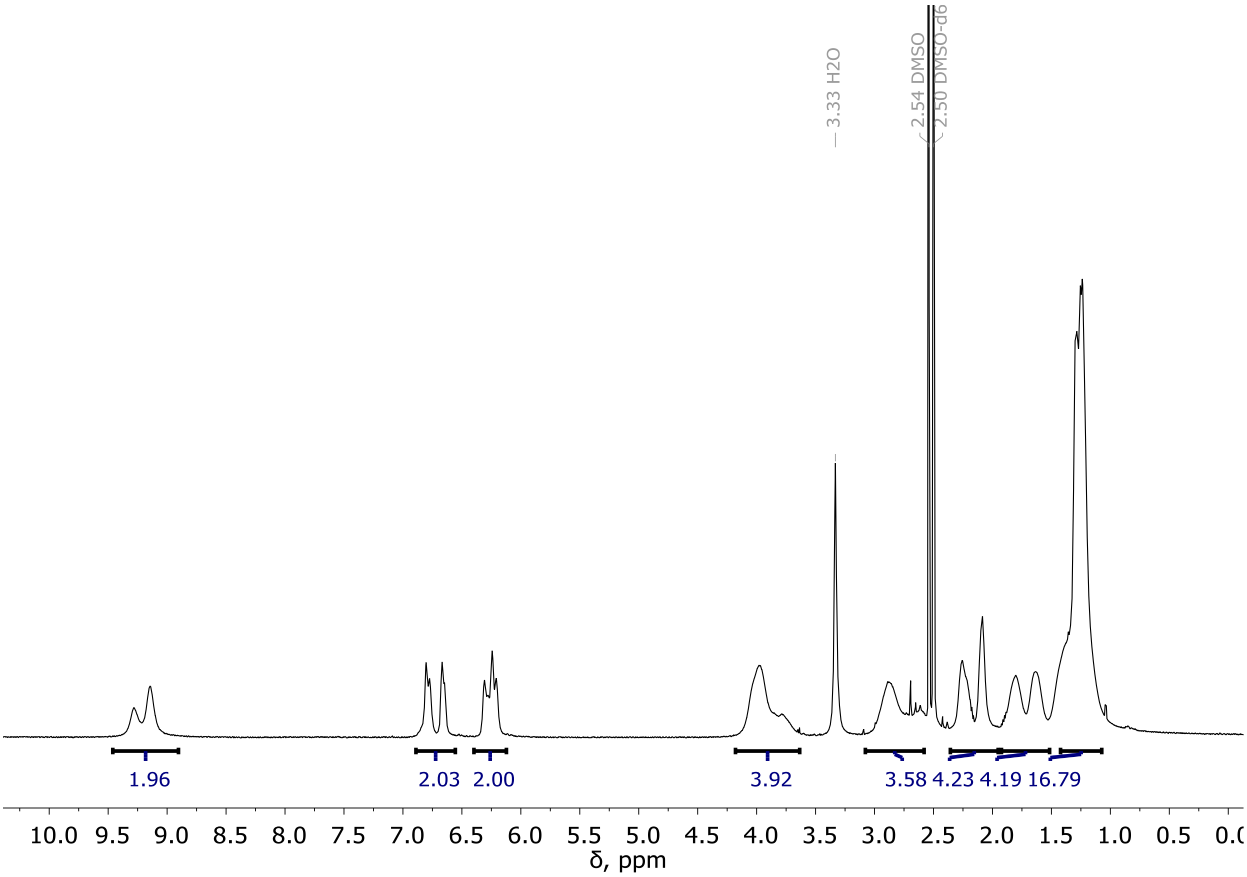

Figure S23. ^{1}H NMR spectrum of **PHU$_5$** in DMSO-d$_6$.

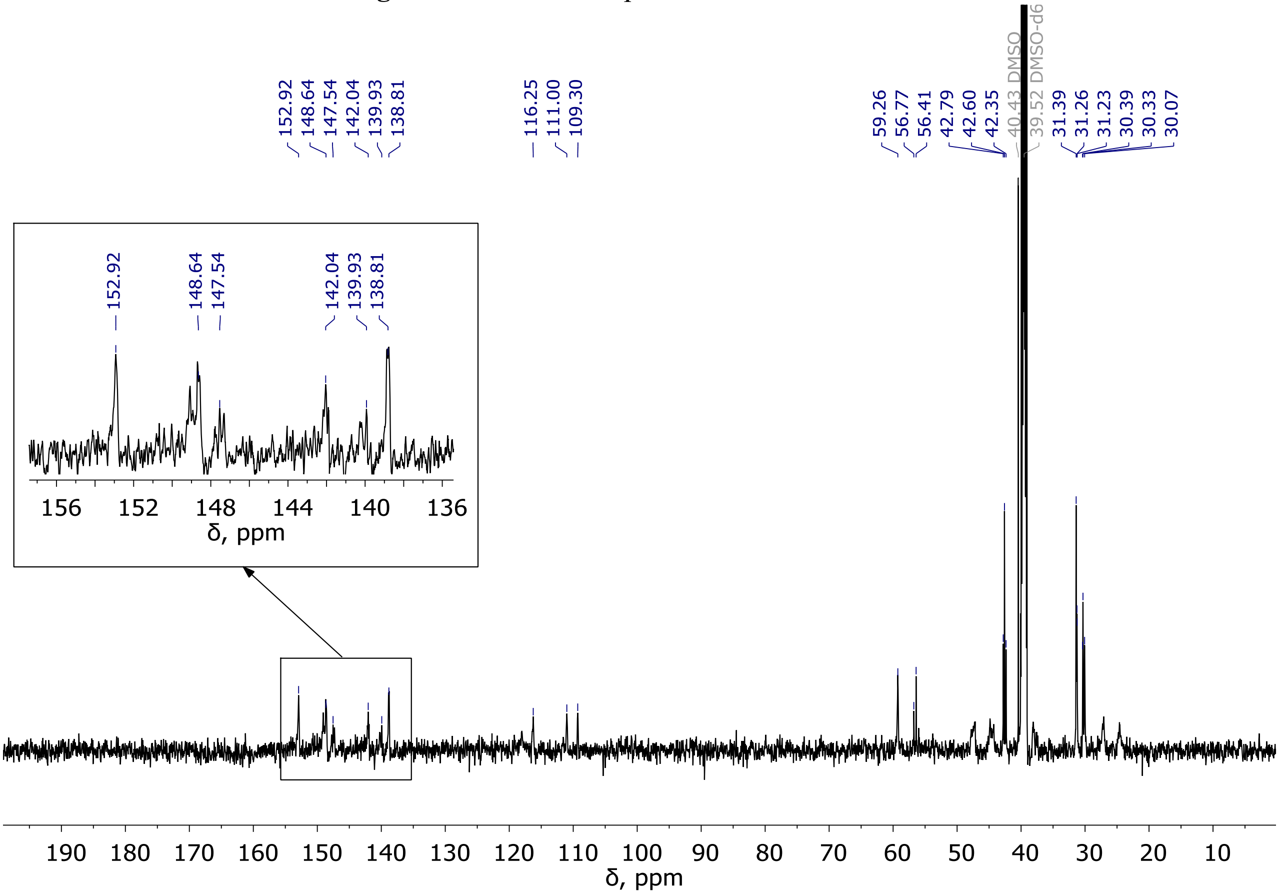

Figure S24. ^{13}C NMR spectrum of **PHU$_5$** in DMSO-d$_6$.

VI.7. Synthesis of PHU based on $\mathbf{M_1}$ and N^1,N^3-dimethylpropane-1,3-diamine ($\mathbf{PHU_6}$).

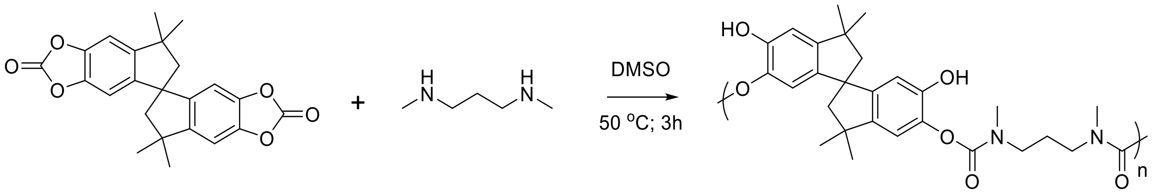

Scheme S9. Synthesis of $\mathbf{PHU_6}$ polymer.

The following loading was applied: $\mathbf{M_1}$ (1.175 g, 3 mmol) and N^1,N^3-dimethylpropane-1,3-diamine (0.306 g, 3 mmol). Yield: 1.19 g (80 %). M_n (GPC) = 5300 g/mol, M_w/M_n = 2.6, DP_n = 11.

T_{onset} (TGA, 5°C/min, on air) = 175°C. T_g (DSC, 5°C/min, sealed under N_2) = 103°C. T_g (TMA in He, 5°C/min) = 125°C.

^{1}H NMR (600 MHz, DMSO-d_6) δ 9.57 – 8.93 (m, 2H), 6.93 – 6.56 (m, 2H), 6.40 – 6.12 (m, 2H), 3.52 – 3.34 (m, 2H), 3.30 – 3.15 (m, 2H), 3.08 – 2.77 (m, 6H), 2.32 – 2.01 (m, 4H), 2.02 – 1.66 (m, 2H), 1.40 – 1.15 (m, 12H).

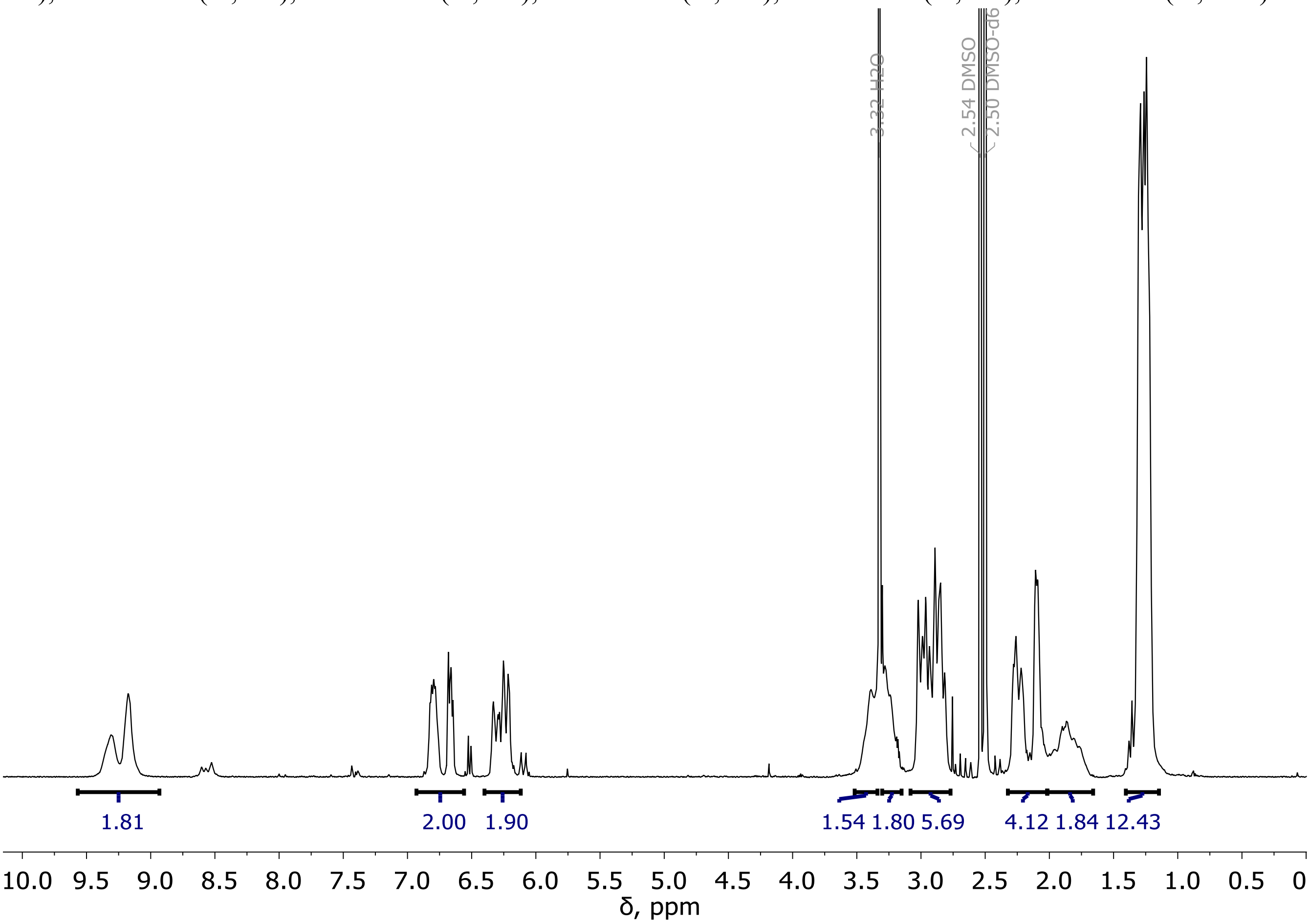

Figure S25. ^{1}H NMR spectrum of $\mathbf{PHU_6}$ in DMSO-d_6.

VI.8. Attempted synthesis of PHU based on **M1** and N[1],N[2]-dicyclohexylethane-1,2-diamine (**PHU7**).

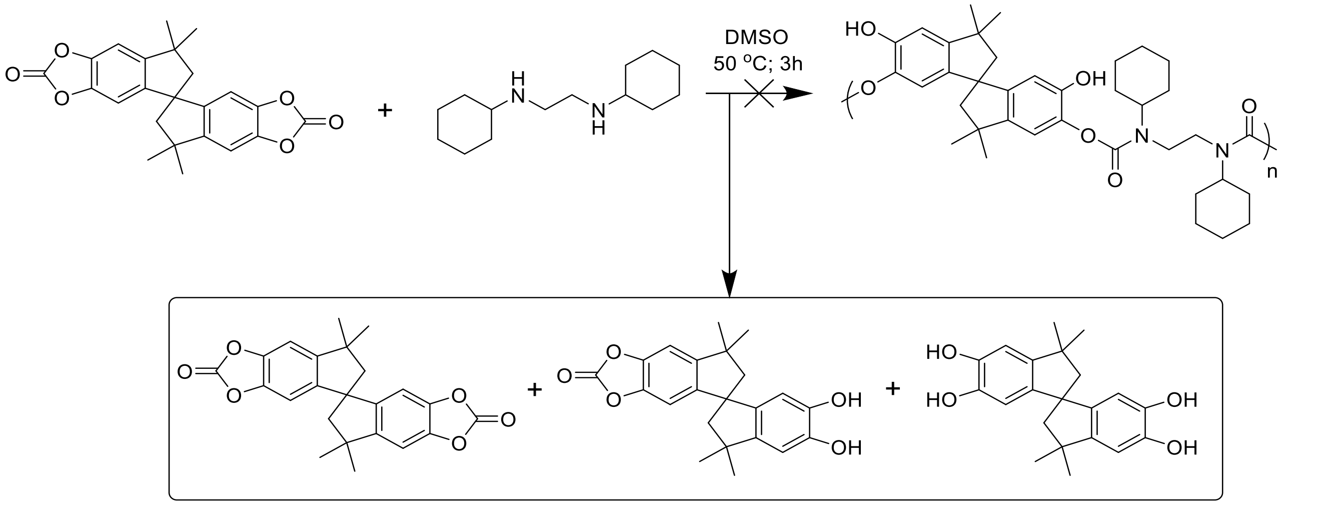

Scheme S10. Attempted synthesis of **PHU7** polymer.

The following loading was applied: **M1** (1.175 g, 3 mmol) and N[1],N[2]-dicyclohexylethane-1,2-diamine (0.672 g, 3 mmol). Yield: 0.90 g (49 %). M_n (GPC) < 1000 g/mol.

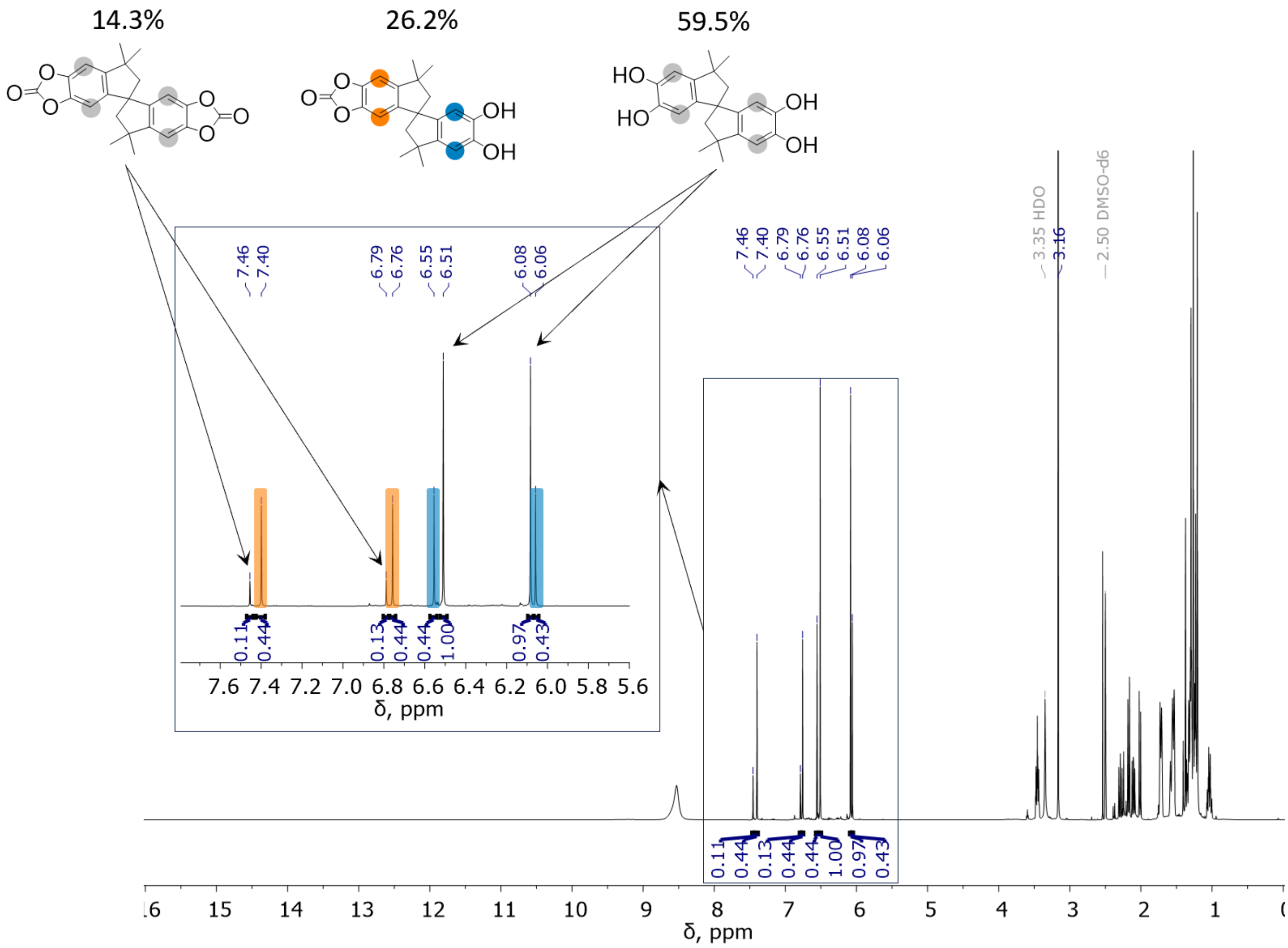

Figure S26. ^{1}H NMR spectrum of attempted synthesis of **PHU7** in DMSO-d6.

VI.9. Synthesis of PHU based on **M_2** and piperazine (**PHU_8**).

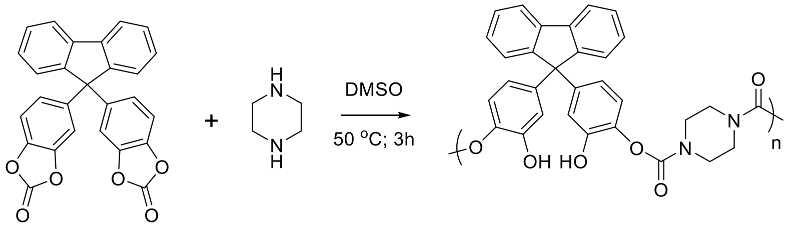

Scheme S11. Synthesis of **PHU_8** polymer.

The following loading was applied: **M_2** (1.96 g, 4.51 mmol), piperazine (0.389 g, 4.51 mmol), DMSO (3 mL). Yield: 2.55 g (96 %). M_n (GPC) = 100300 g/mol, M_w/M_n = 4.3, DP_n = 193.

T_{onset} (TGA, 5°C/min, on air) = 200°C; T_{onset} (TGA, 5°C/min, N_2) = 280°C. T_g (DSC, 5°C/min, sealed under N_2) = 211°C, T_g (TMA in He, 5°C/min) = 259°C.

^{1}H NMR (600 MHz, DMSO-d_6) δ 9.70 – 9.37 (m, 2H), 8.04 – 7.83 (m, 2H), 7.56 – 7.27 (m, 6H), 6.99 – 6.43 (m, 6H), 3.44 (d, J = 88.8 Hz, 8H).

^{13}C NMR (151 MHz, DMSO-d_6) δ 152.78, 152.59, 150.63, 148.78, 148.72, 148.07, 147.98, 143.77, 143.47, 139.40, 138.42, 137.76, 137.69, 135.86, 127.89, 125.93, 122.97, 122.42, 120.51, 118.27, 116.48, 116.20, 64.23, 63.78, 63.32.

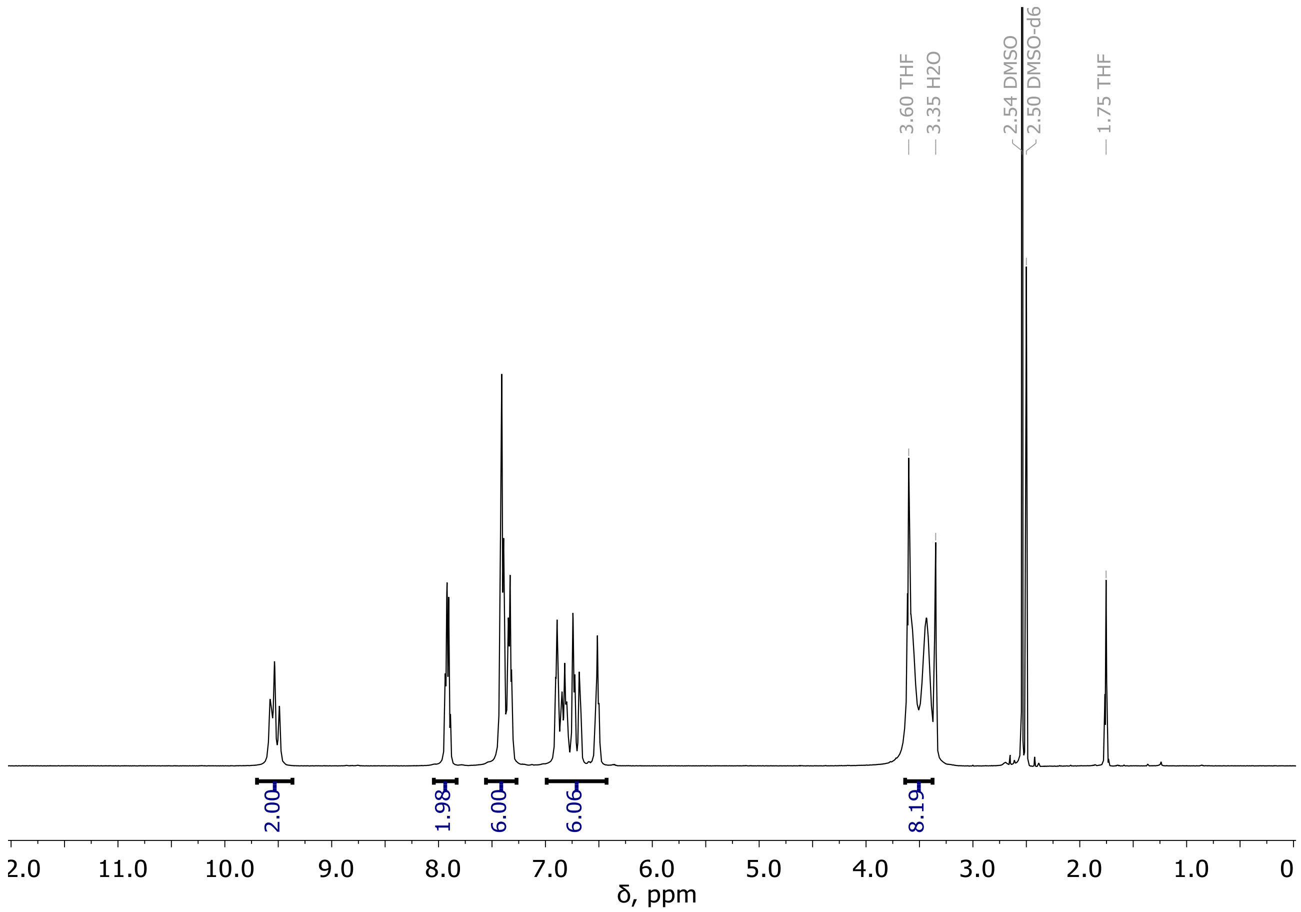

Figure S27. ^{1}H NMR spectrum of **PHU_8** in DMSO-d_6.

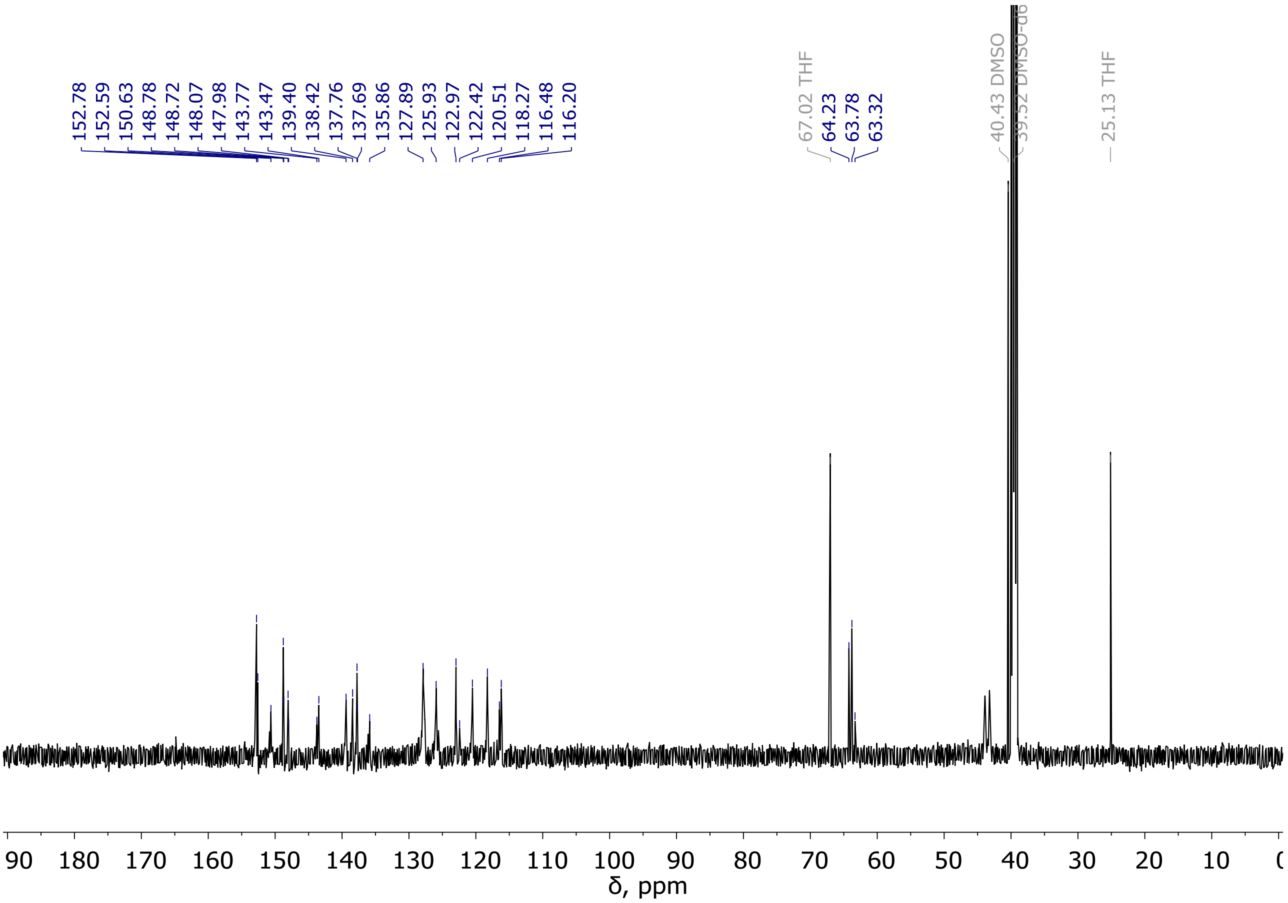

Figure S28. ^{13}C NMR spectrum of **PHU$_8$** in DMSO-d$_6$.

VI.10. Synthesis of PHU based on **M$_2$** and 1,3-di(piperidin-4-yl)propane (**PHU$_9$**).

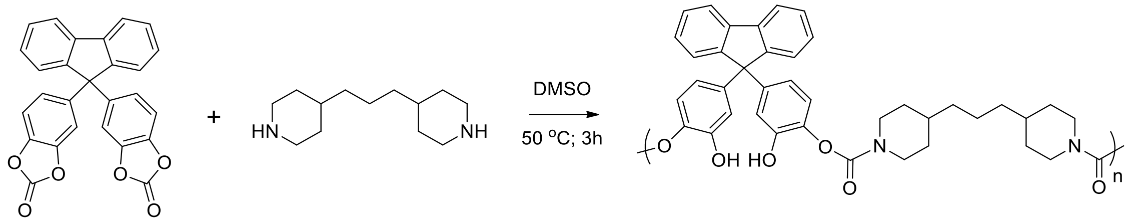

Scheme S12. Synthesis of **PHU$_9$** polymer.

The following loading was applied: **M$_2$** (1.96 g, 4.51 mmol), 1,3-di(piperidin-4-yl)propane (0.949 g, 4.51 mmol), DMSO (3 mL). Yield: 2.70 g (93 %). M_n (GPC) = 28900 g/mol, M_w/M_n = 3.8, DP_n = 45.

T_{onset} (TGA, 5°C/min, on air) = 265°C; T_{onset} (TGA, 5°C/min, N_2) = 265°C. T_g (DSC, 5°C/min, sealed under N_2) = 194°C. T_g (TMA in He, 5°C/min) = 207°C.

^{1}H NMR (600 MHz, DMSO-d$_6$) δ 9.31 (s, 1H), 7.90 (t, J = 9.3 Hz, 2H), 7.51 – 7.17 (m, 6H), 6.89 – 6.29 (m, 6H), 3.98 (d, J = 81.2 Hz, 4H), 3.33 (s, 2H), 2.67 (d, J = 92.9 Hz, 4H), 1.63 (s, 4H), 1.47 – 1.06 (m, 12H).

^{13}C NMR (151 MHz, DMSO-d$_6$) δ 152.67, 152.52, 150.69, 148.89, 143.18, 139.39, 139.28, 138.66, 138.01, 127.81, 125.90, 122.95, 120.48, 118.11, 116.34, 116.14, 64.21, 63.76, 44.46, 44.00, 36.01, 34.93, 31.66, 31.44, 22.94.

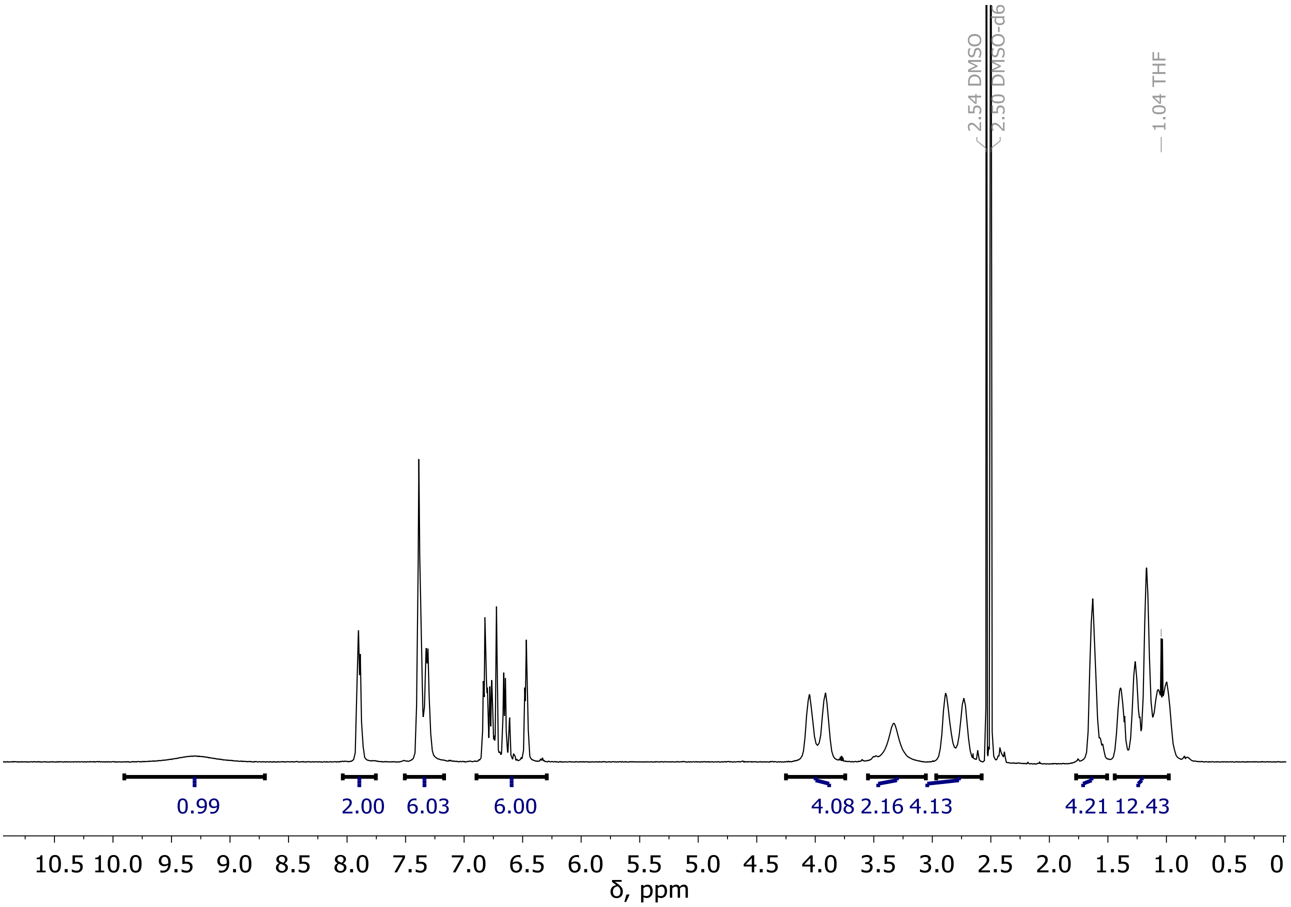

Figure S29. ^{1}H NMR spectrum of **PHU₉** in DMSO-d₆.

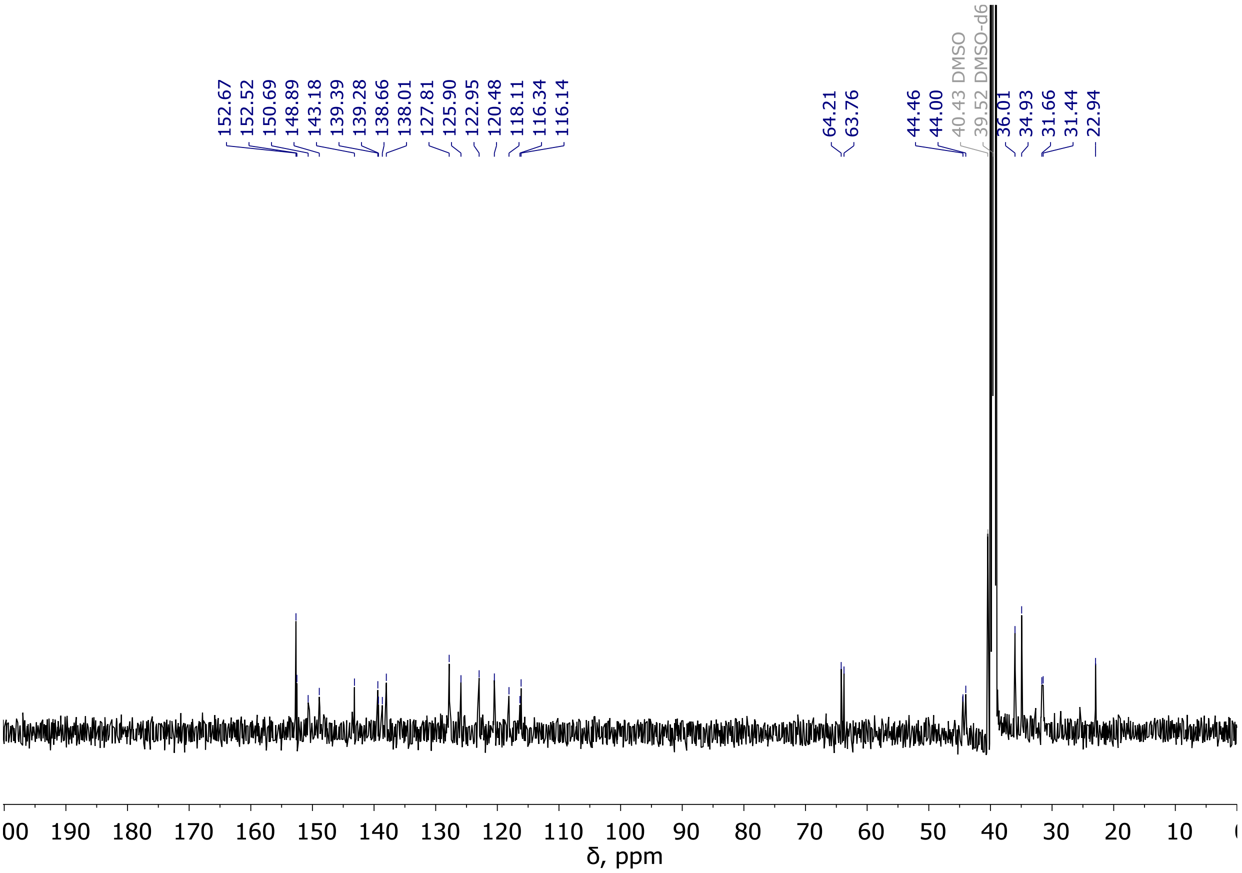

Figure S30. ^{13}C NMR spectrum of **PHU₉** in DMSO-d₆.

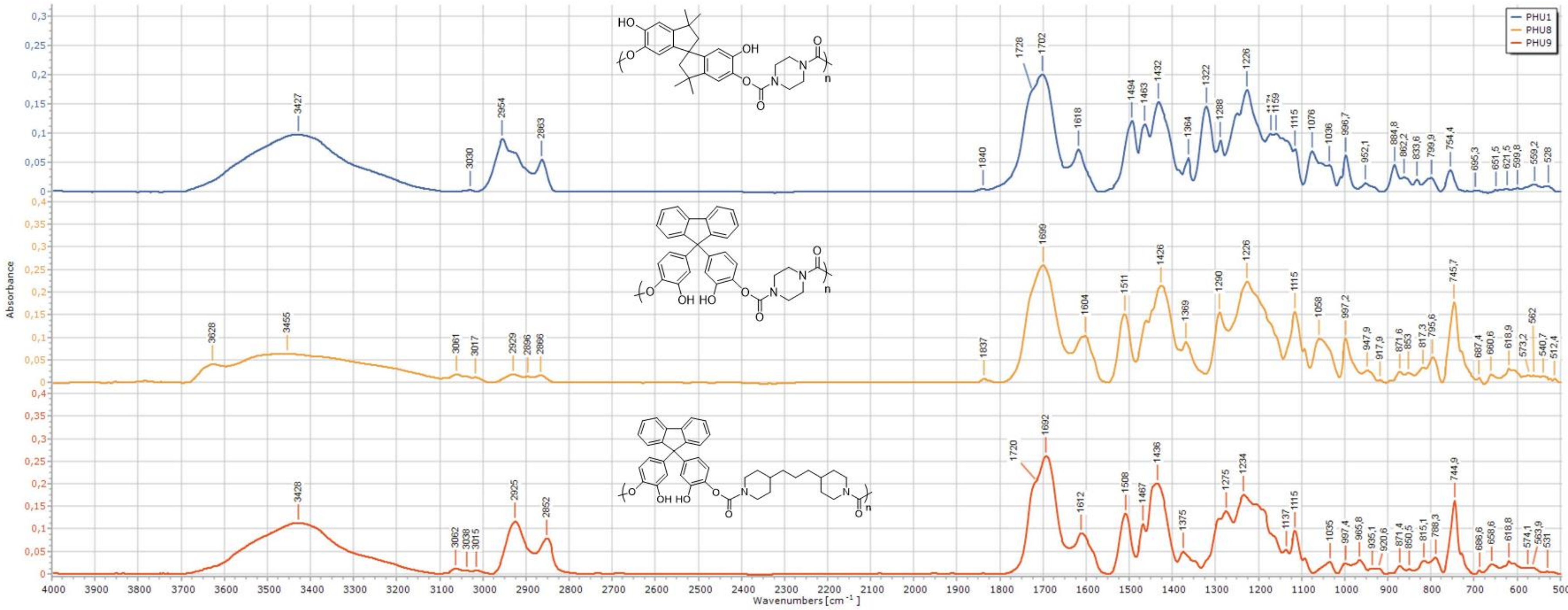

Figure S31. IR spectra of **PHU₁**, **PHU₈**, and **PHU₉** (recorded as KBr pellets; the broad absorption band at 3420–3455 cm⁻¹ arises in part from moisture in KBr).

HIT-NO=1299	SCORE= ( )	SDBS-NO=1108	IR-NIDA-61043 : KBR DISC

1.3-DIETHYLUREA

$C_5H_{12}N_2O$

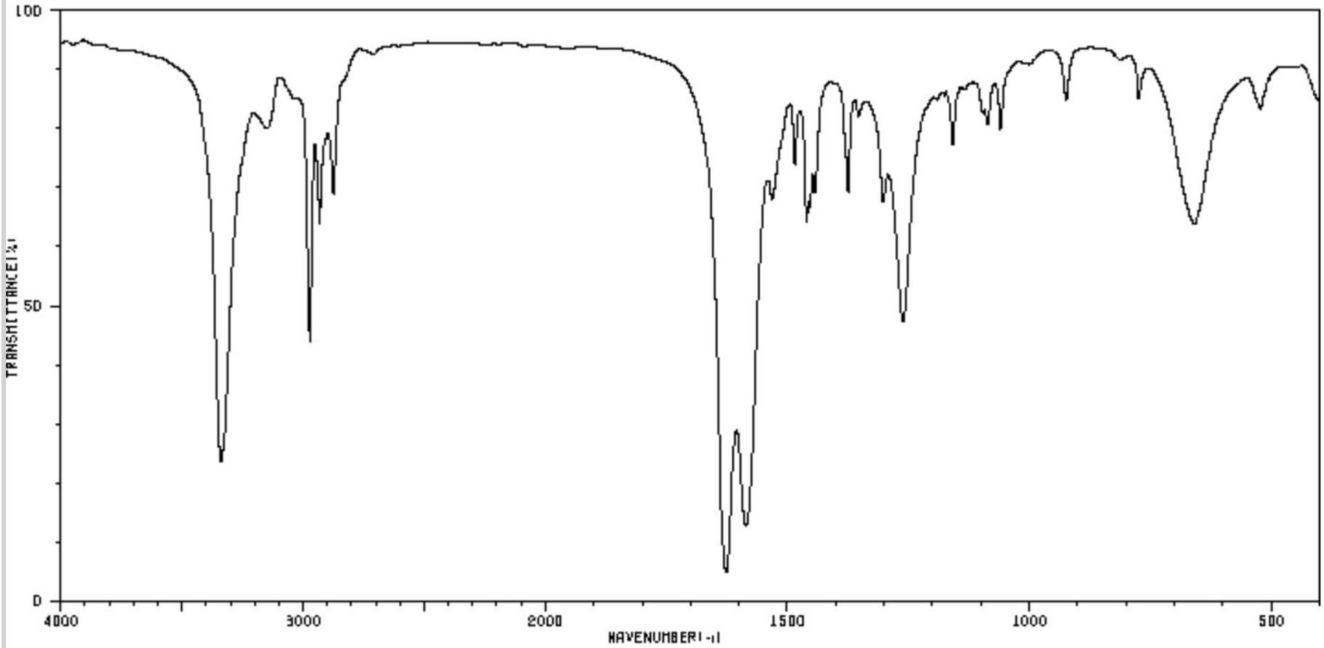

3339	23	1626	4	1376	66	1060	77
3157	77	1586	12	1354	79	924	81
3147	77	1531	66	1303	86	774	81
3136	77	1486	70	1261	46	669	62
2972	42	1461	62	1159	74	529	81
2932	82	1454	64	1096	79	523	79
2875	66	1443	66	1086	77		

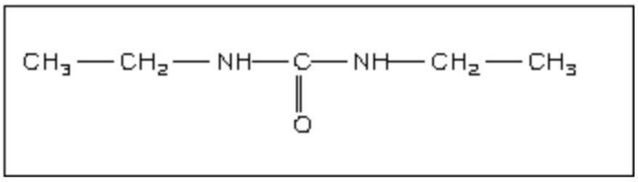

Figure S32. IR spectra of 1.3-diethylurea reproduced from ref. [35]

VII. Solubility of the obtained PHUs.

Table S2. Solubility table for $\mathbf{M_1}$ and $\mathbf{PHU_1}$.[a]

**Solvent[b]**	**M_1**		**PHU_1**	
	25°C	60°C	25°C	60°C
H_2O	n.d.	n.d.	–	–
Acetone	n.d.	n.d.	–	n.d.
DCM	+	n.d.	–	n.d.
Chloroform	+	n.d.	–	n.d.
Trichloroethane	n.d.	n.d.	–	–
THF	+	+	+	+
DMF	+	n.d.	+	+
DMSO	+	n.d.	+	+
DMAc	+	n.d.	+	+
NMP	+	n.d.	+	+
Methanol	n.d.	n.d.	–	–
Acetonitrile	n.d.	n.d.	–	–
Diethyl Ether	n.d.	n.d.	–	–
Cyclohexane	n.d.	n.d.	–	–
Cyclohexanone	n.d.	n.d.	–	+
Ethyl Acetate	n.d.	n.d.	–	–
Toluene	–	+	–	–
Dioxane	n.d.	n.d.	–	+

[a] Solubility tests were conducted with approximately 0.05 g of polymer immersed in 1 ml of selected solvent in a transparent vial equipped with a magnetic stirrer. The resulting mixture was stirred for 15 mins at the selected temperature, and the solubility or non-solubility of the sample was determined visually; [b] DCM – dichloromethane, THF – tetrahydrofuran, DMF – dimethylformamide, NMP – N-methyl pyrrolidone, DMSO – dimethyl sulfoxide, DMAc – dimethylacetamide.

VIII. Possible side reactions during polyaddition of linear secondary diamines.

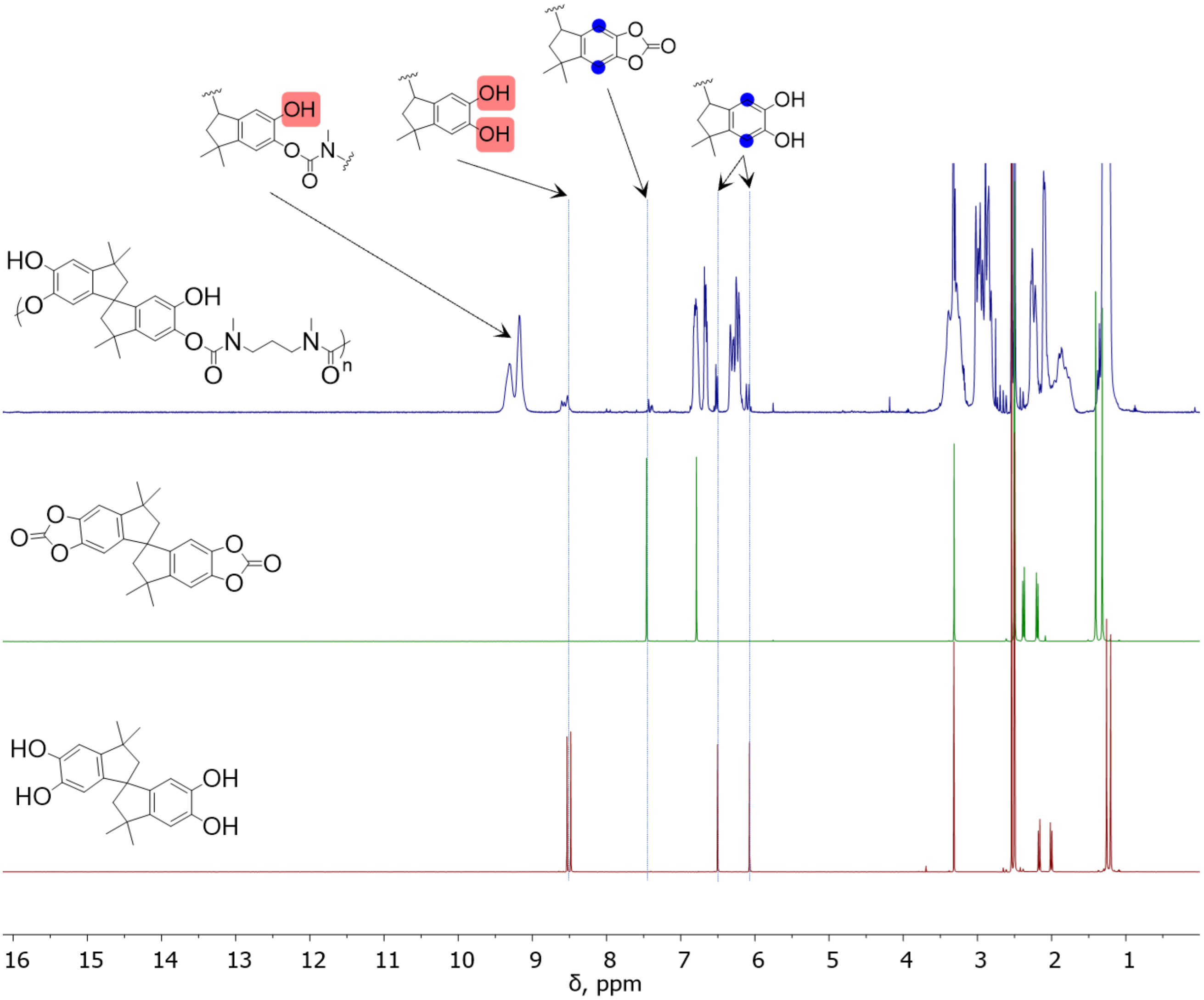

Figure S33. Overlay of ^{1}H NMR traces for reaction products of synthesis of **PHU$_6$**, **M$_1$** dicarbonate and its precursor 5,5',6,6'-Tetrahydroxy-3,3,3',3'-tetramethyl-1,1'-spirobiindane.

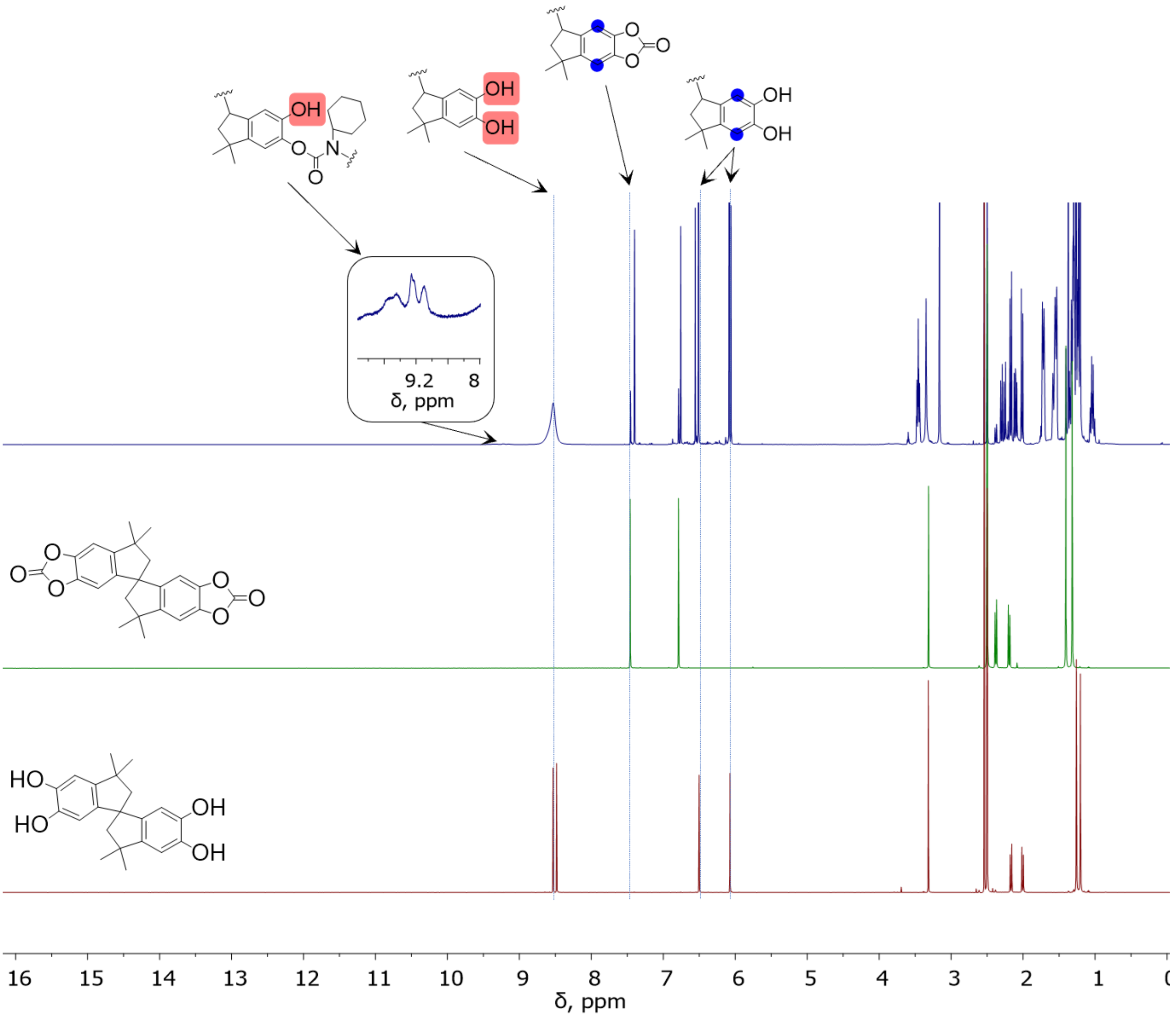

Figure S34. Overlay of ^{1}H NMR traces for reaction products of attempted synthesis of **PHU_7**, **M_1** dicarbonate and its precursor 5,5',6,6'-Tetrahydroxy-3,3,3',3'-tetramethyl-1,1'-spirobiindane.

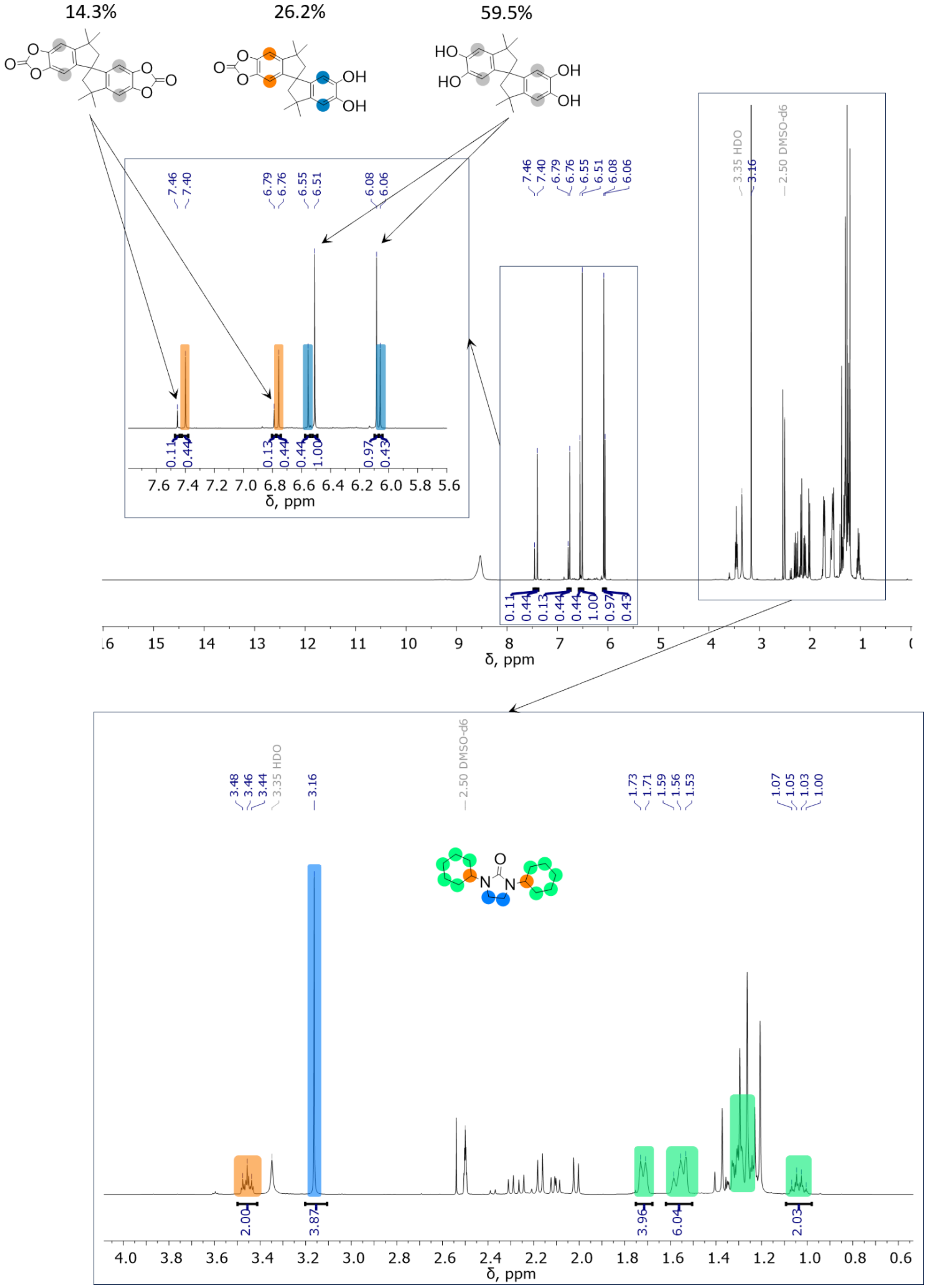

Figure S35. Detailed analysis of ^{1}H NMR spectrum of precipitate obtained during the attempted synthesis of **PHU**$_7$.

IX. Molecular weight determination via GPC.

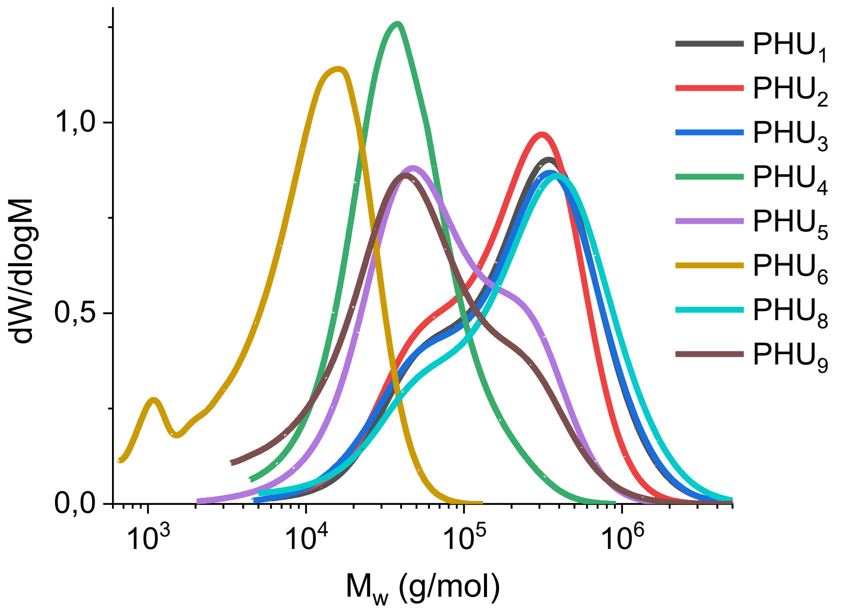

Figure S36. GPC traces of **PHU_1 - PHU_9** in 0.1 M $Li(CF_3SO_2)_2N$ (LiTFSI) solution in DMF at 50°C.

X. Molecular weight determination via sedimentation-diffusion analysis.

Table S3. Molecular weight determination using sedimentation-diffusion analysis [a].

Sample	[η], dl/g	$s_0 \times 10^{13}$ [b]	$D_0 \times 10^7$ [c]	dn/dc	$M_{sD} \times 10^{-3}$ [d]
**PHU_1**	0.68	2.7	3.4	0.138	71.2
**PHU_3**	0.90	2.3	2.5	0.127	82.5

[a] Conditions: DMF, 25°C, $\rho_0 = 0.9446$, $\eta_0 = 0.787$;
[b] s_0 is the sedimentation coefficient at infinite dilution;
[c] D_0 is the translational diffusion coefficient;
[d] M_{sD} is absolute molecular weight calculated using the Svedberg equation (**eq.S3**).

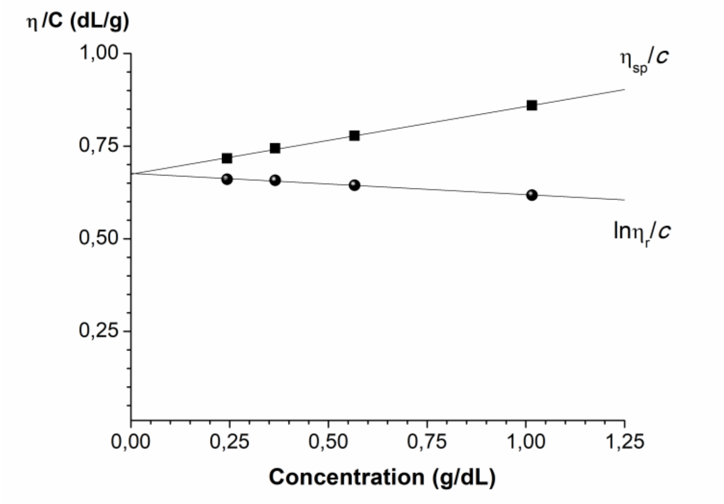

Figure S37. Intrinsic viscosity of **PHU_1** measured in DMF at 25.0°C.

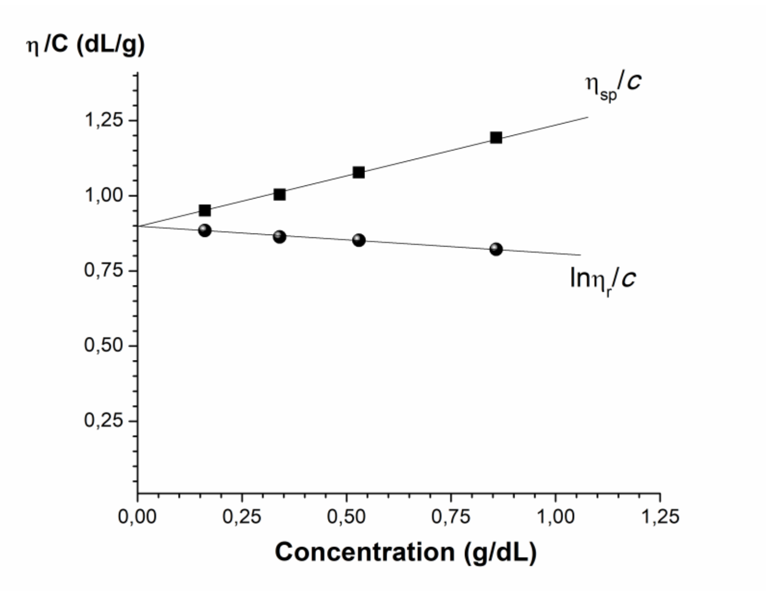

Figure S38. Intrinsic viscosity of **PHU$_3$** measured in DMF at 25.0°C.

XI. TMA plots of PHUs.

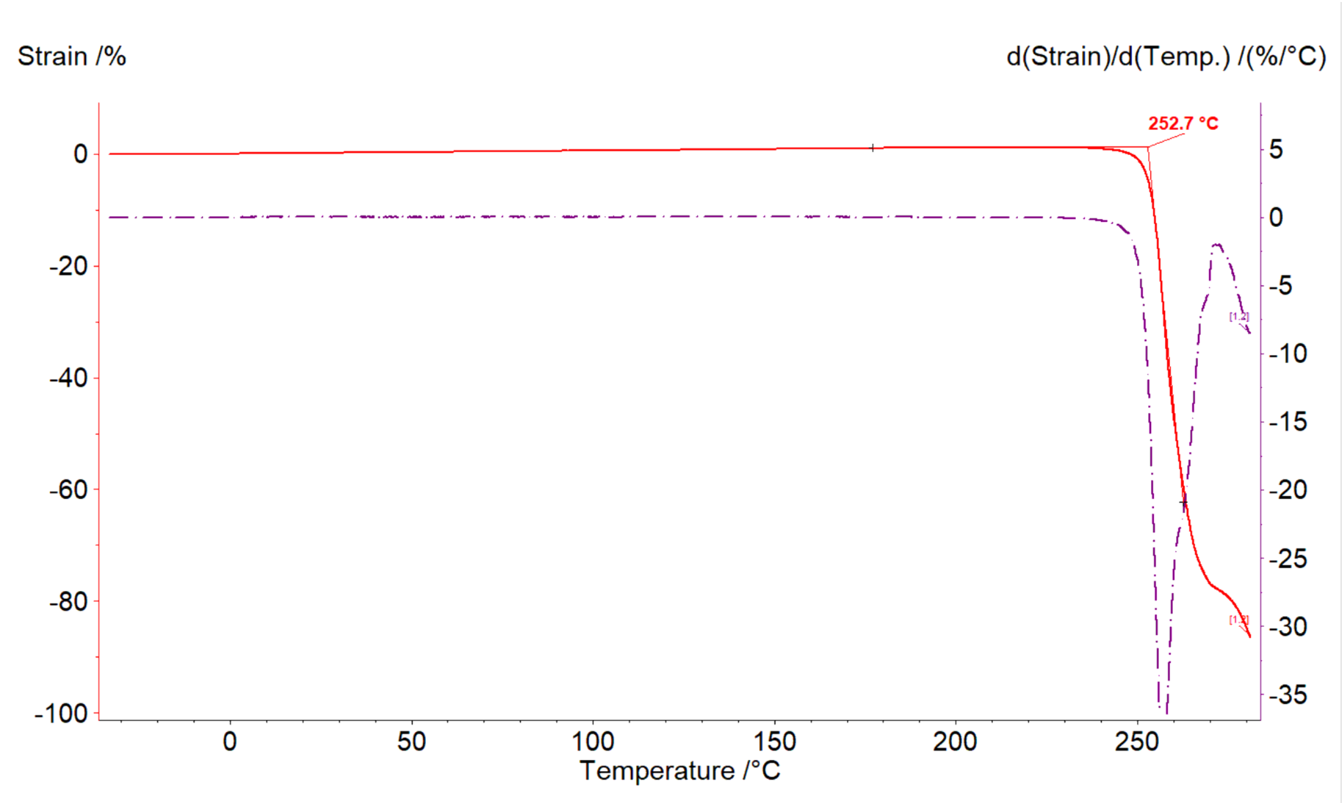

Figure S39. TMA plot of **PHU$_1$** at a heating rate of 5°C/min under inert atmosphere (He).

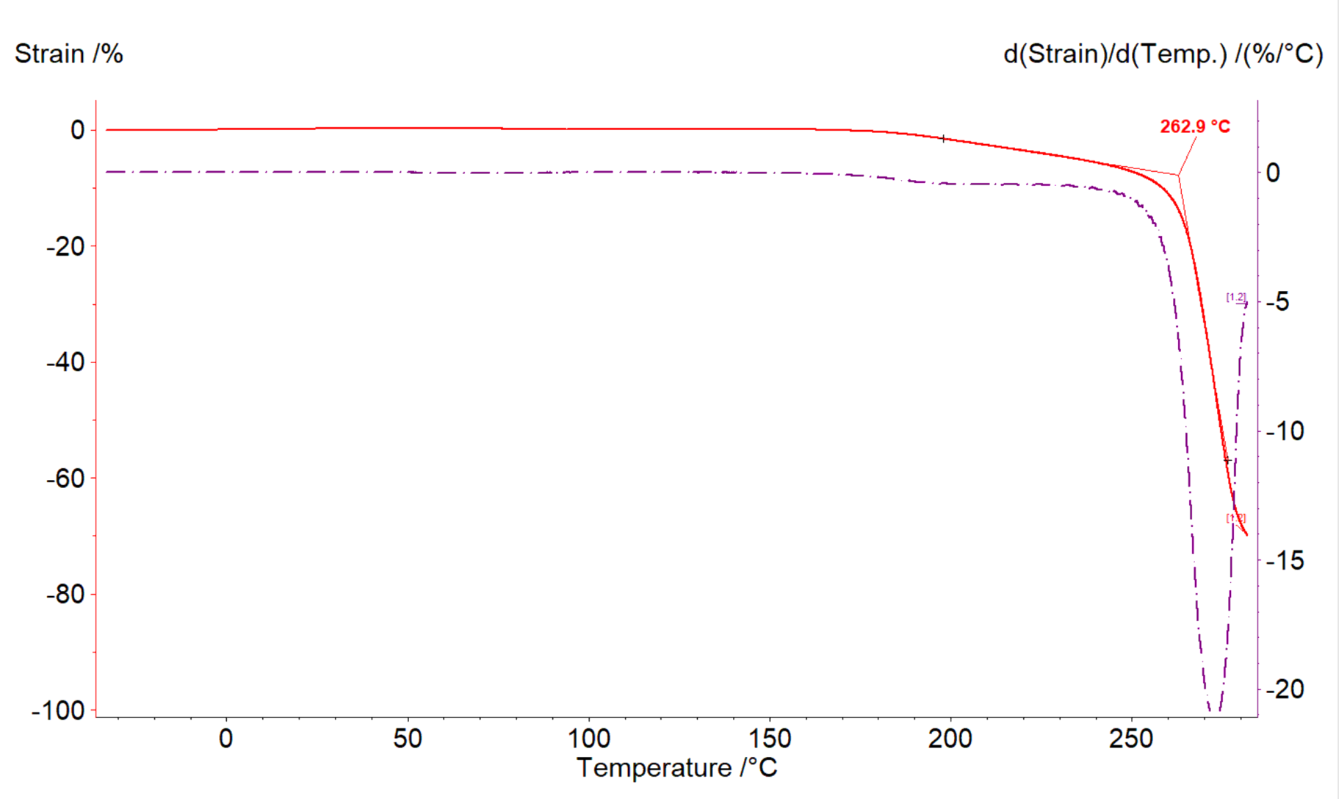

Figure S40. TMA plot of **PHU$_2$** at a heating rate of 5°C/min under inert atmosphere (He).

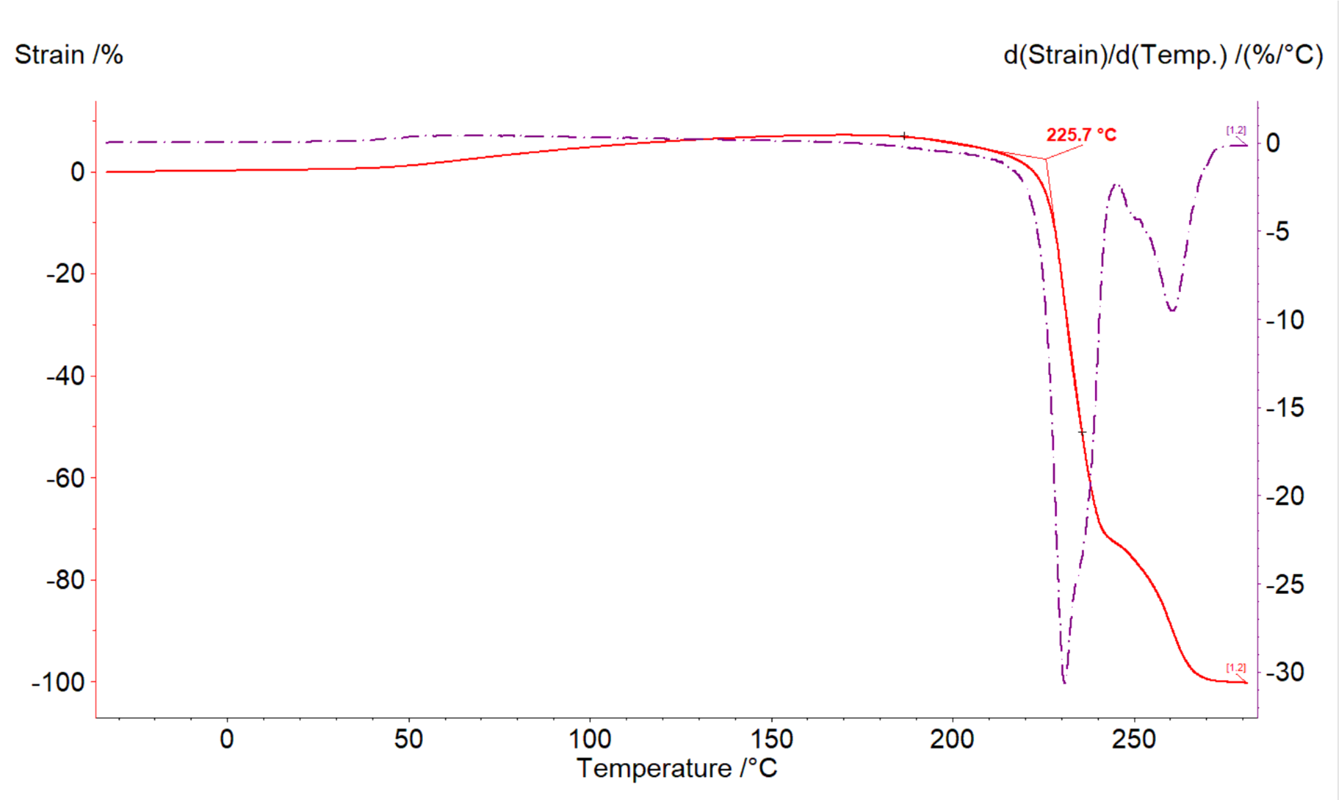

Figure S41. TMA plot of **PHU$_3$** at a heating rate of 5°C/min under inert atmosphere (He).

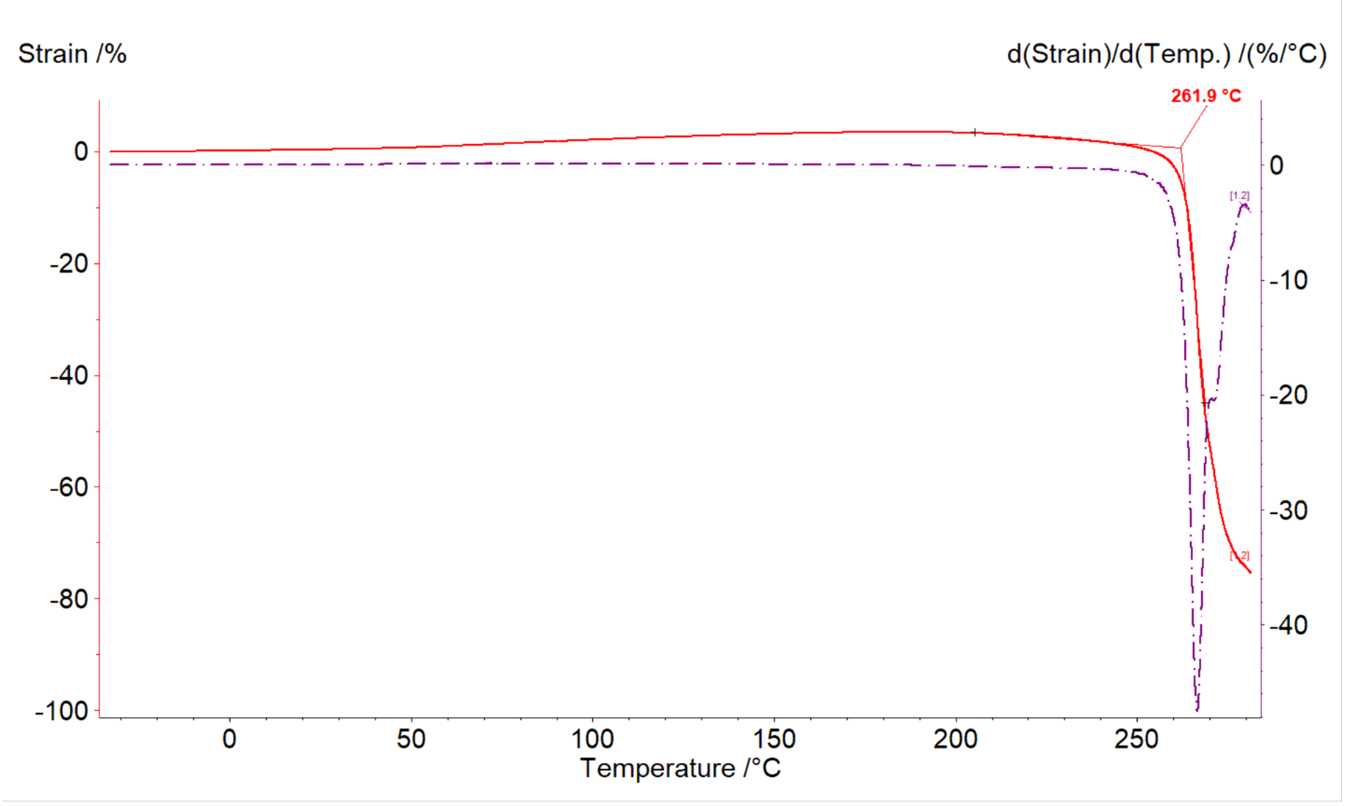

Figure S42. TMA plot of **PHU**$_4$ at a heating rate of 5°C/min under inert atmosphere (He).

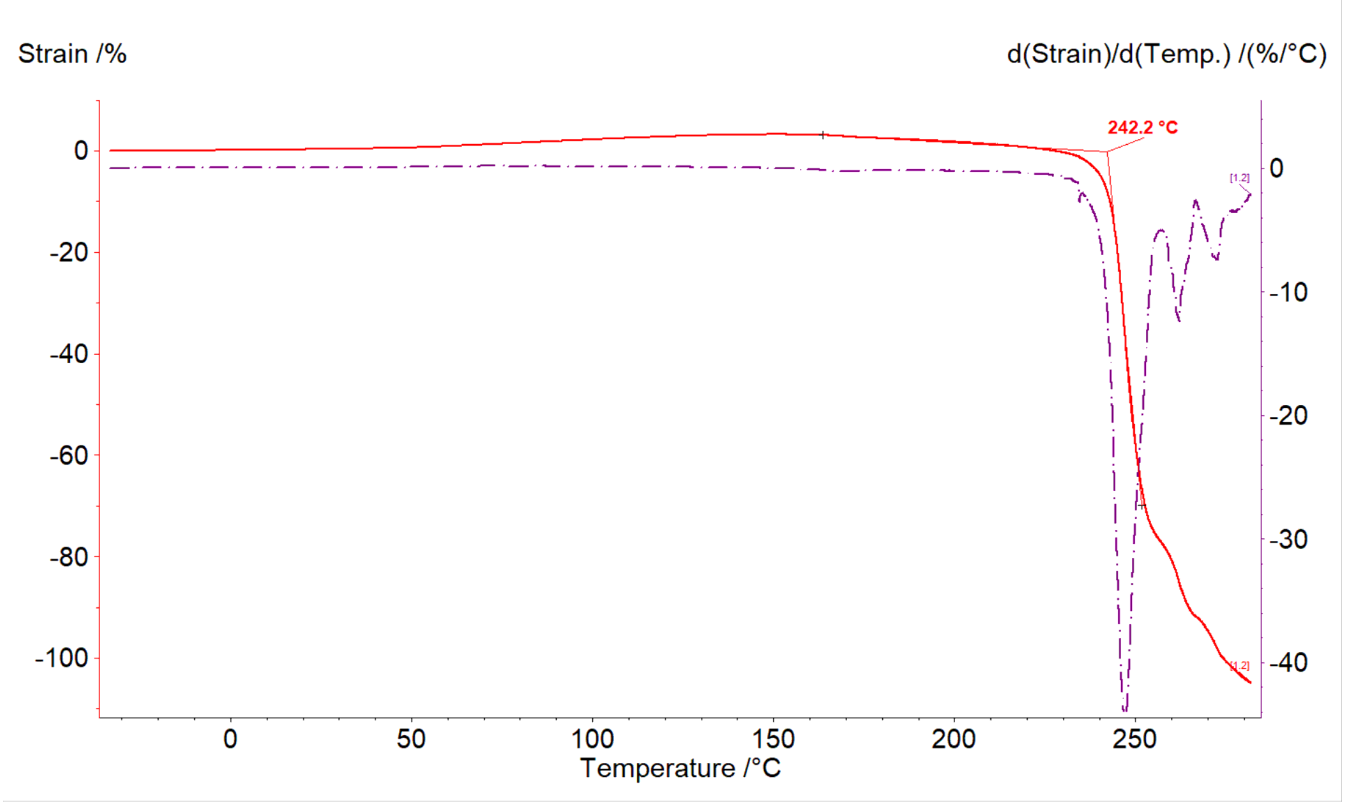

Figure S43. TMA plot of **PHU**$_5$ at a heating rate of 5°C/min under inert atmosphere (He).

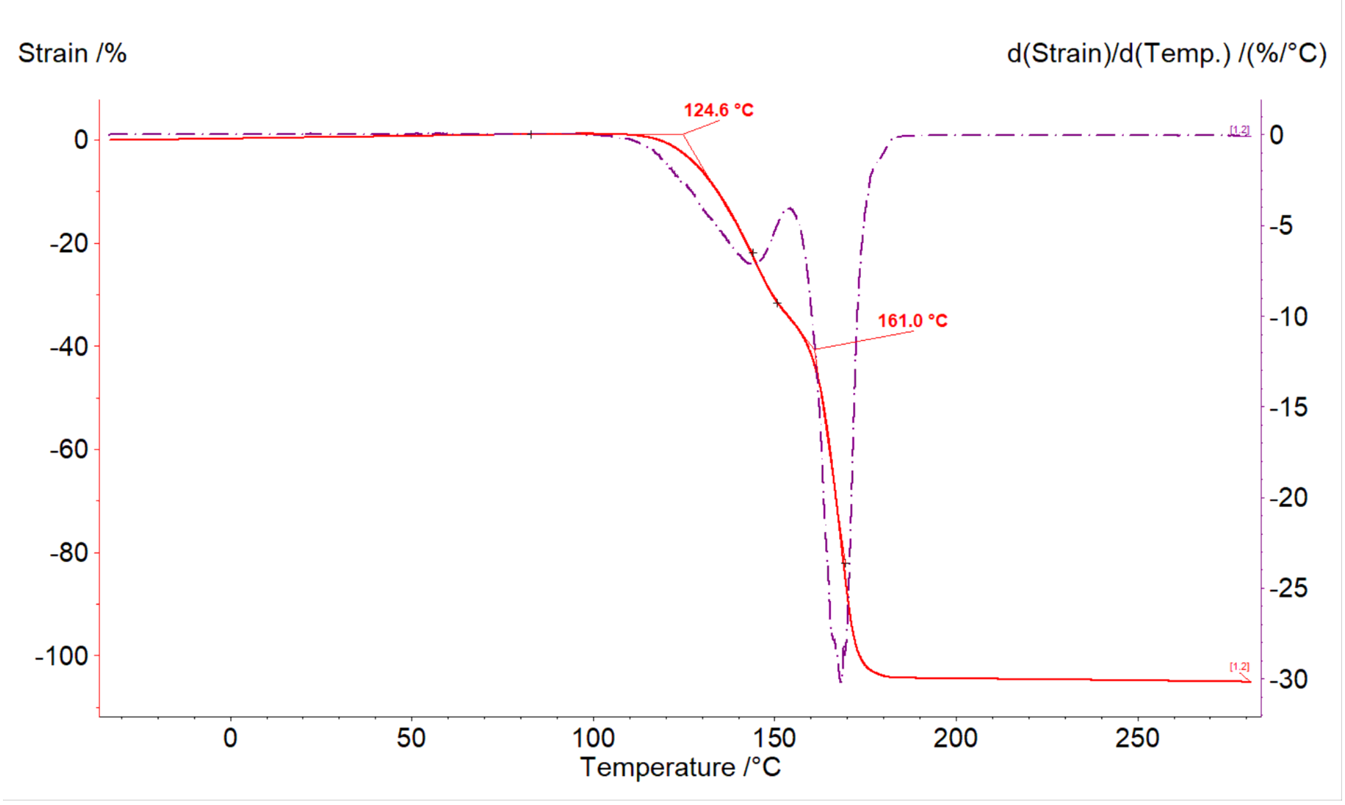

Figure S44. TMA plot of **PHU$_6$** at a heating rate of 5°C/min under inert atmosphere (He).

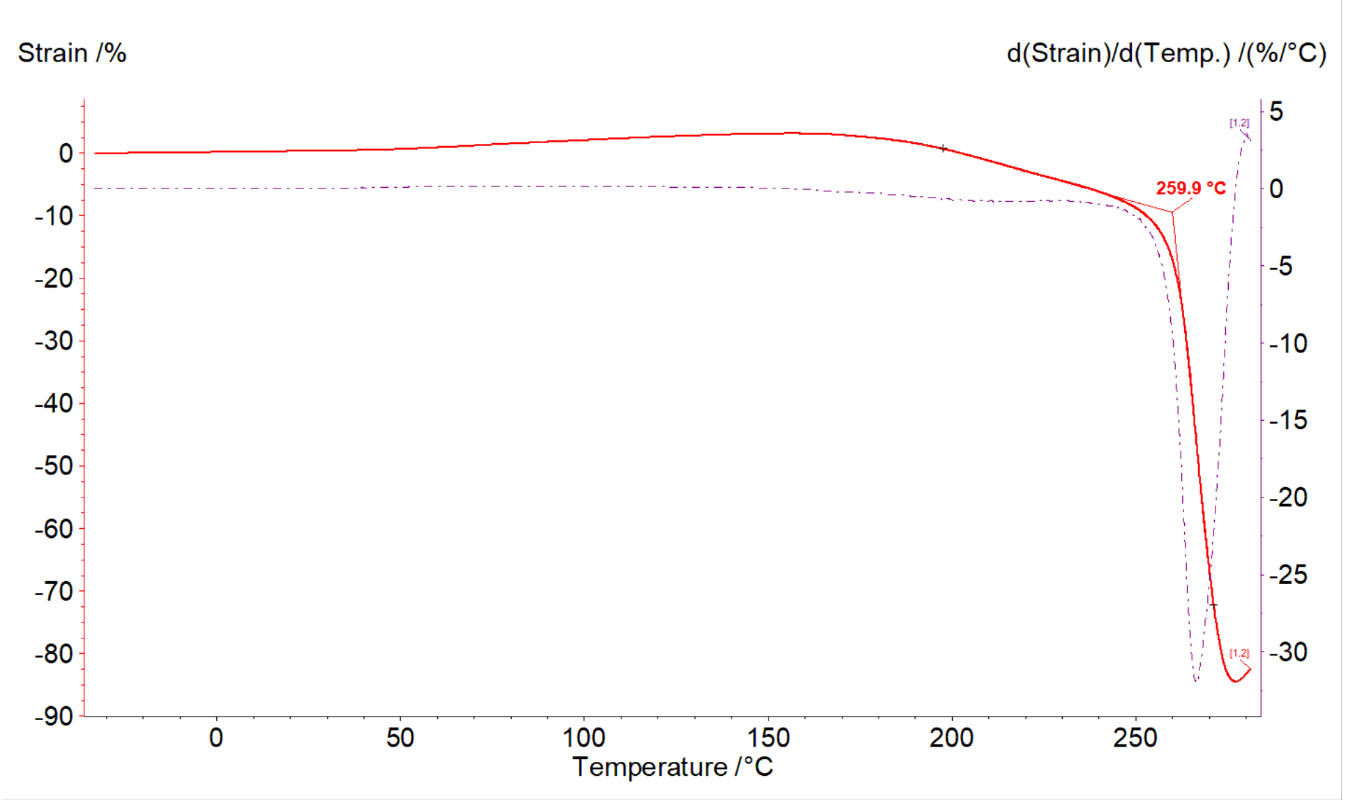

Figure S45. TMA plot of **PHU$_8$** at a heating rate of 5°C/min under inert atmosphere (He).

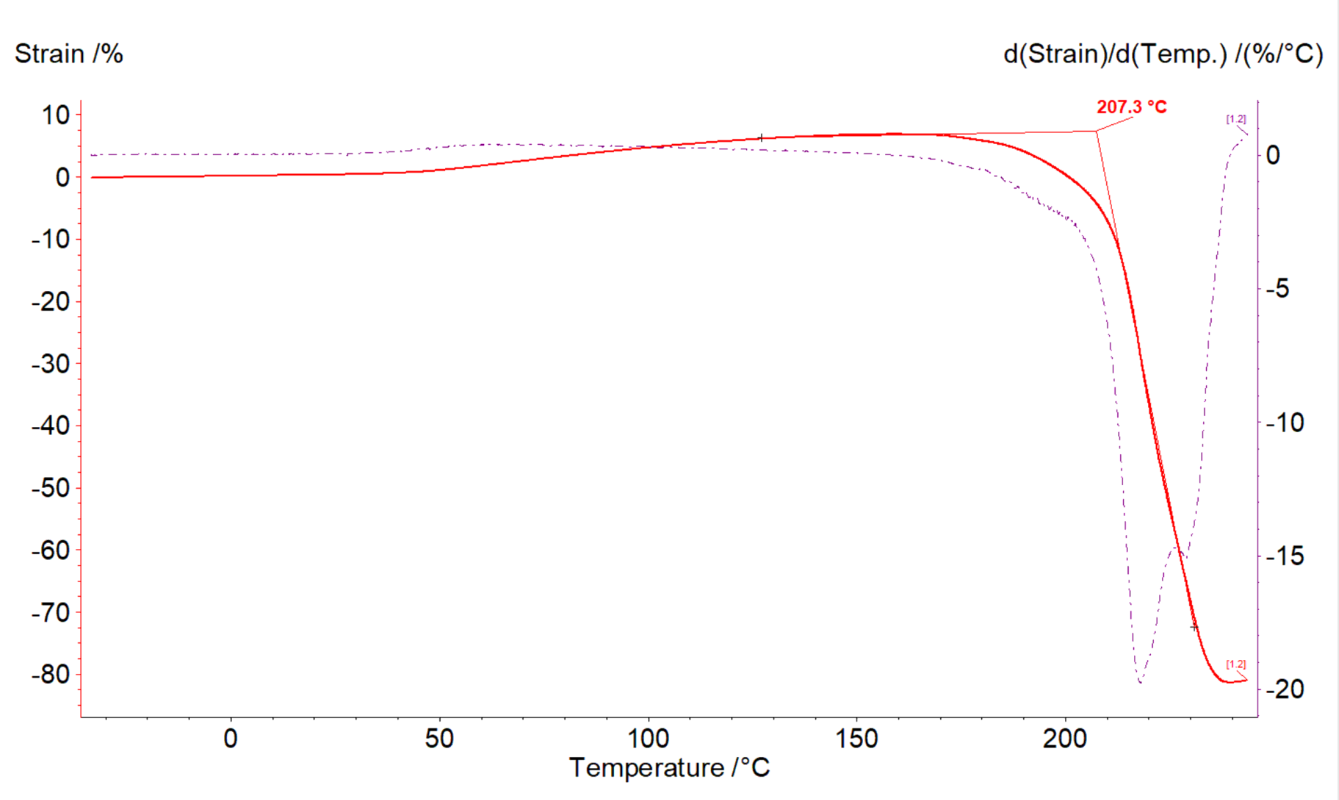

Figure S46. TMA plot of **PHU$_9$** at a heating rate of 5°C/min under inert atmosphere (He).

XII. DSC plots of PHUs.

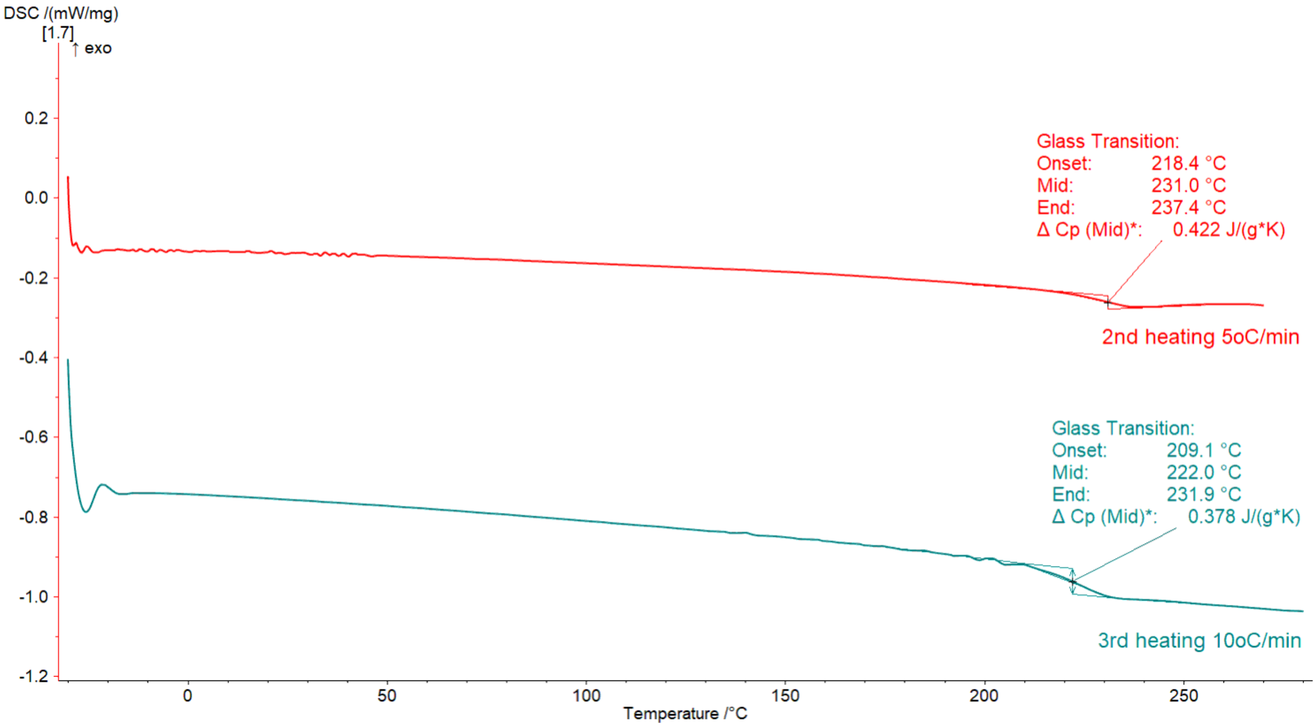

Figure S47. DSC plot of **PHU$_2$**.

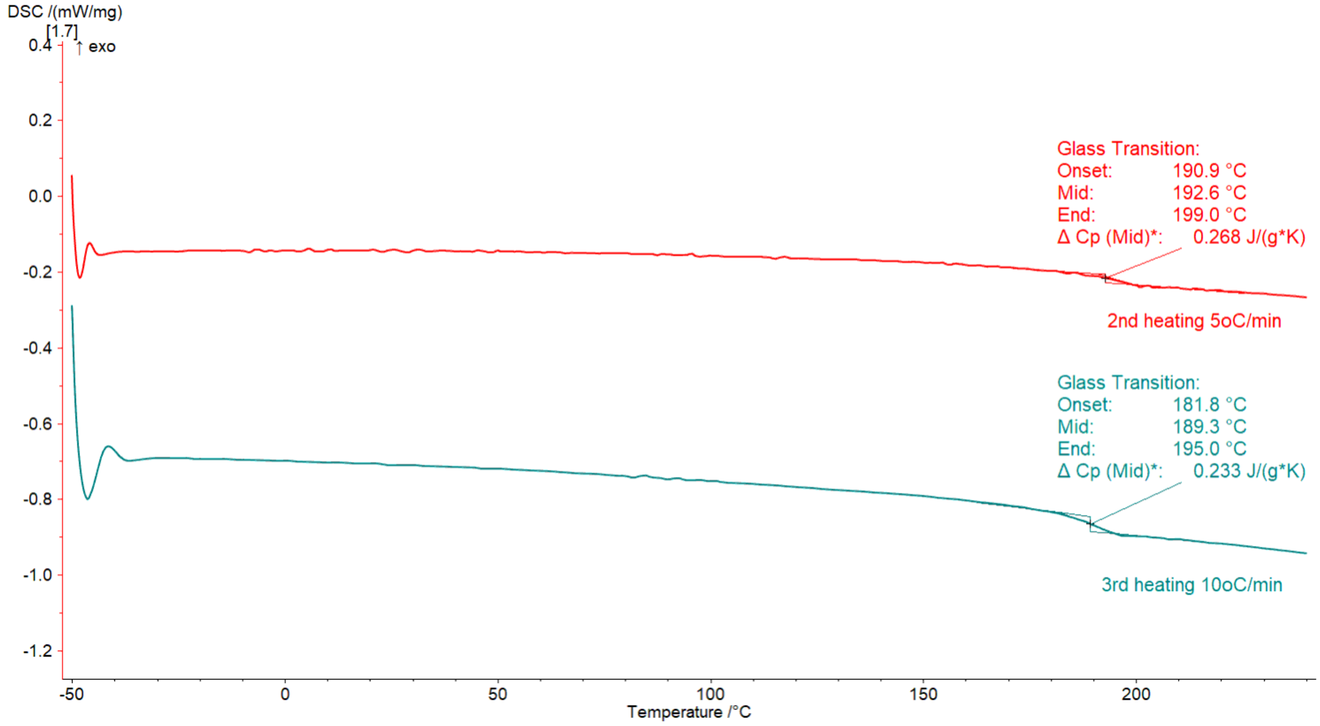

Figure S48. DSC plot of **PHU**$_3$.

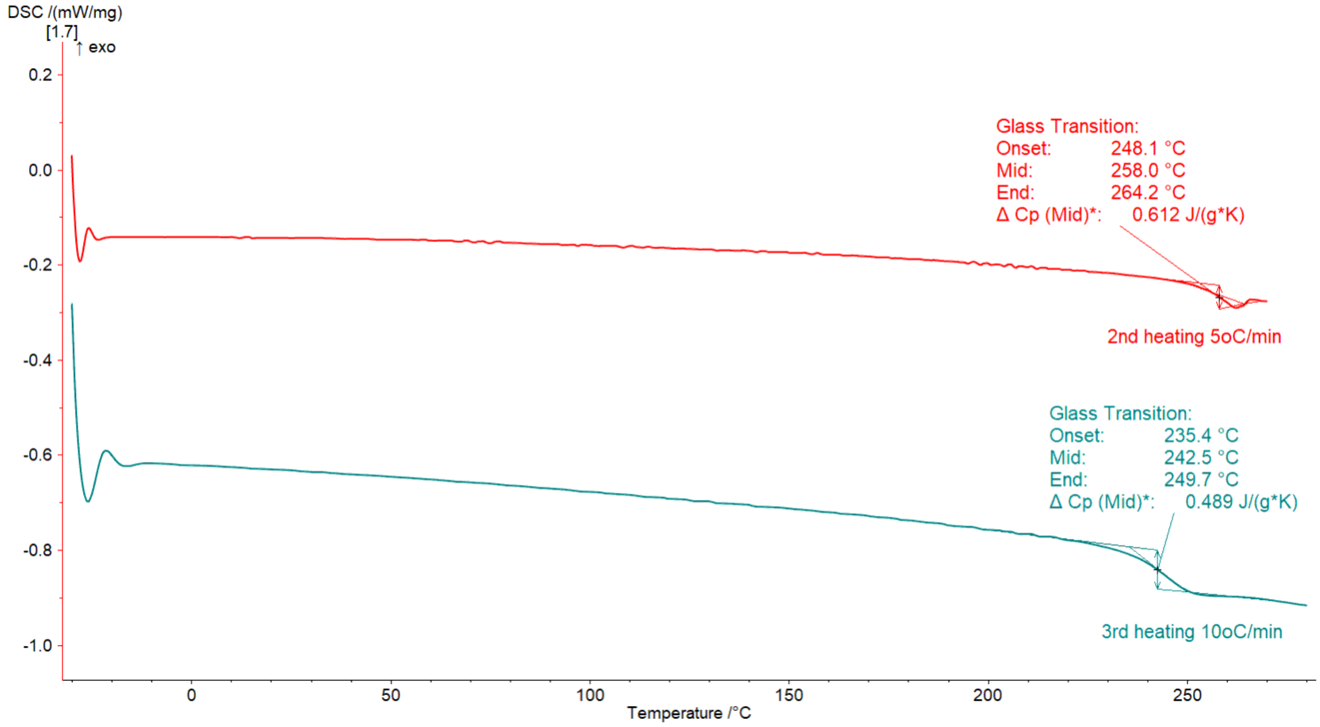

Figure S49. DSC plot of **PHU**$_4$.

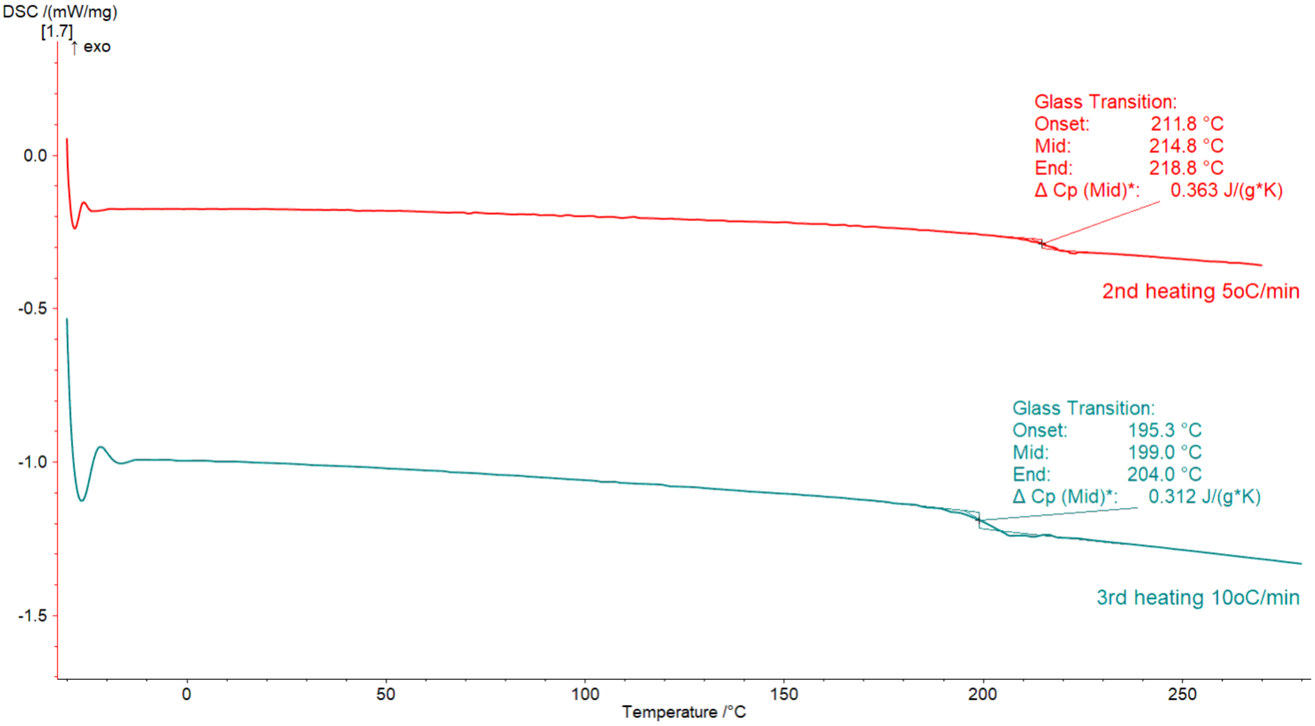

Figure S50. DSC plot of **PHU$_5$**.

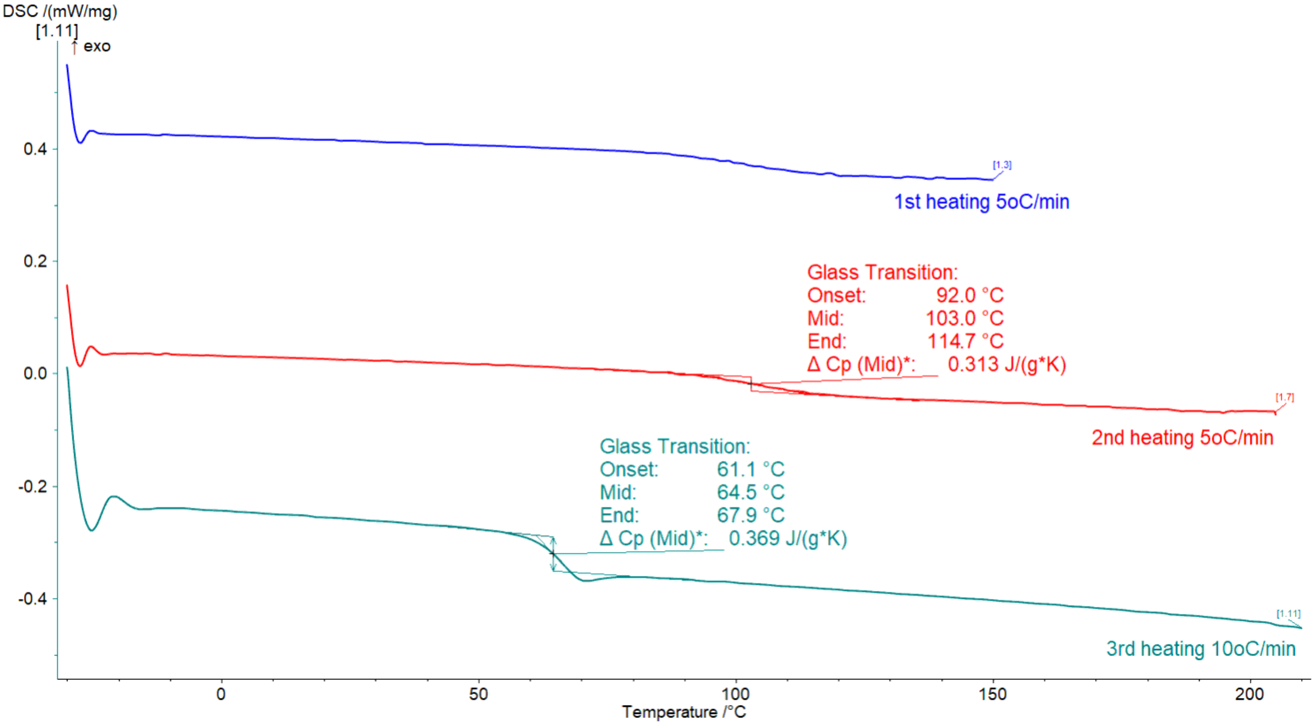

Figure S51. DSC plot of **PHU$_6$**.

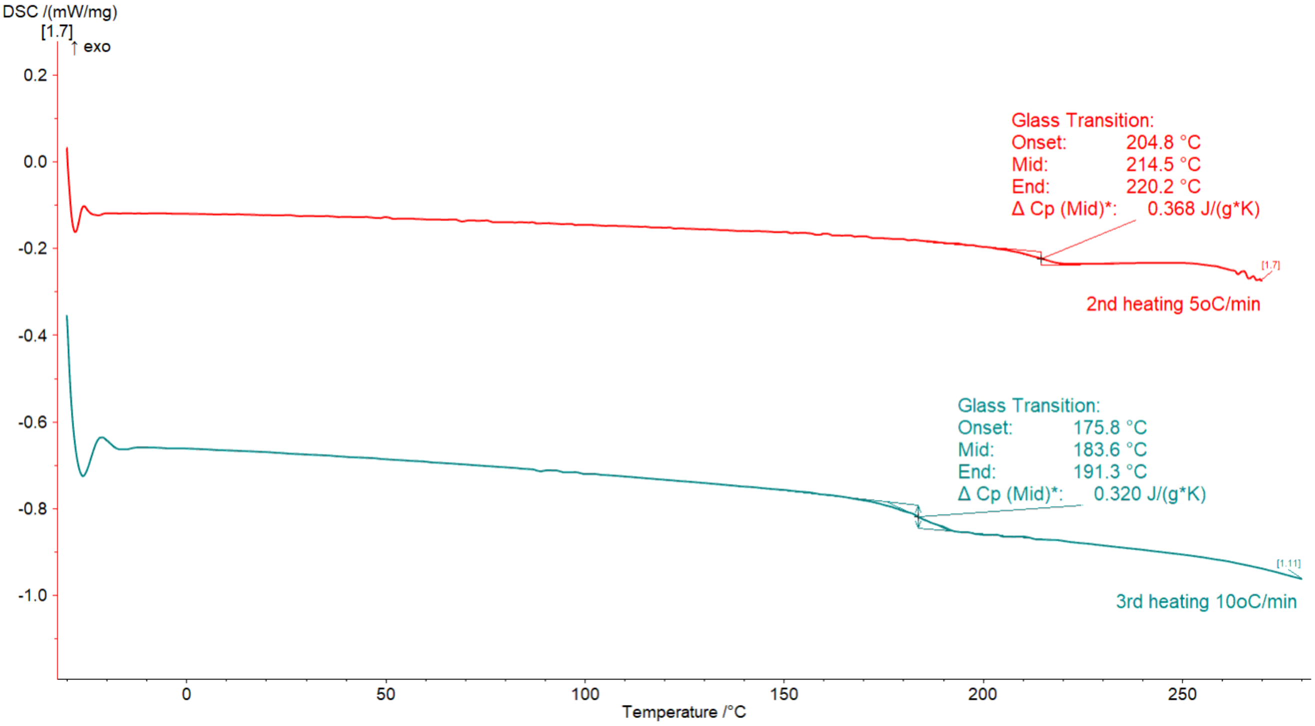

Figure S52. DSC plot of **PHU**$_{\mathbf{8}}$.

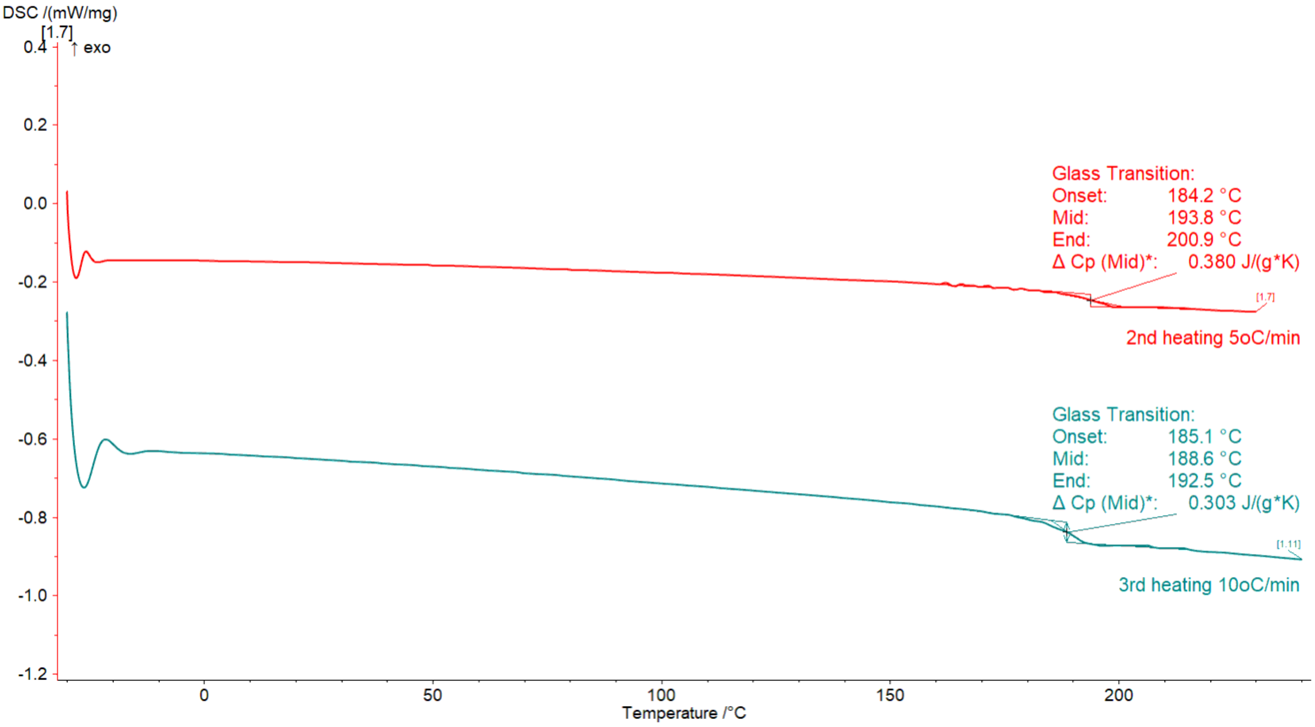

Figure S53. DSC plot of **PHU**$_{\mathbf{9}}$.

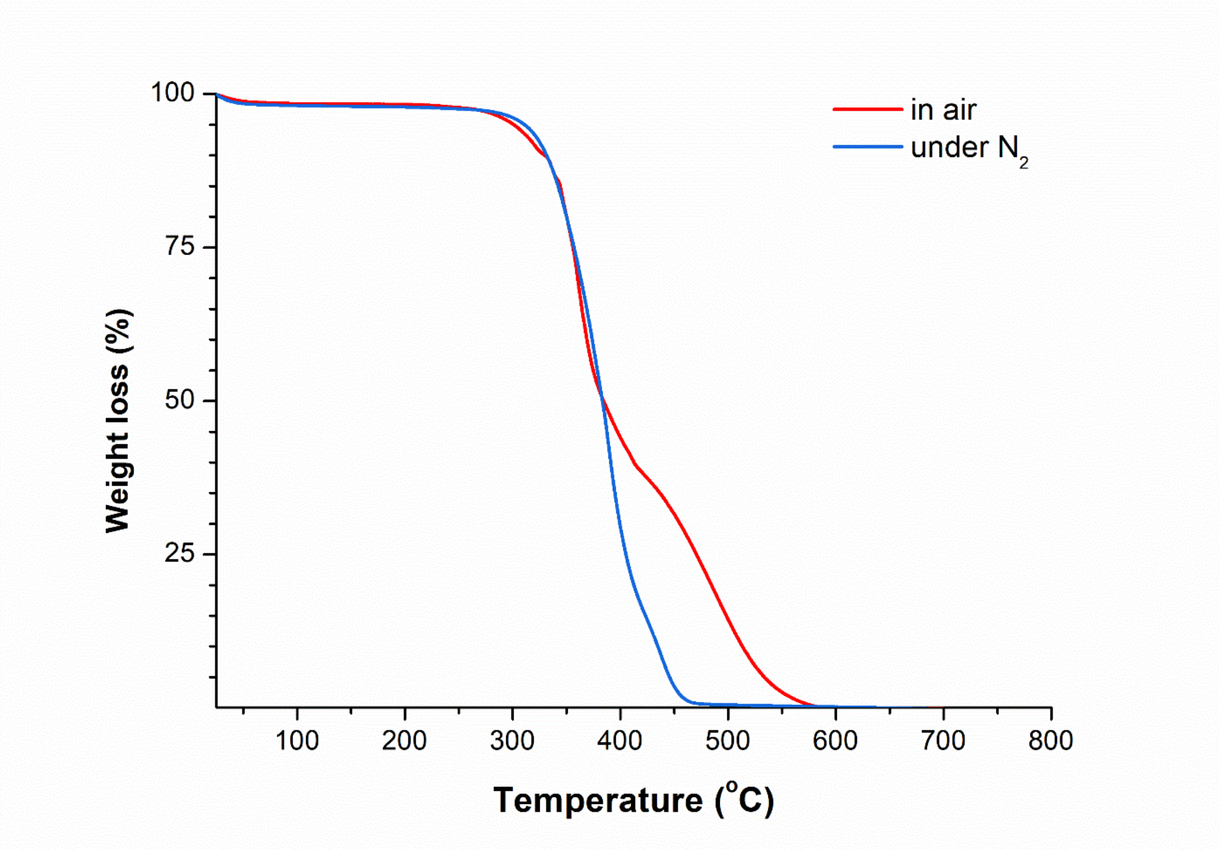

Figure S54. Representative TGA plots of **PHU$_3$** at a heating rate of 5°C/min.

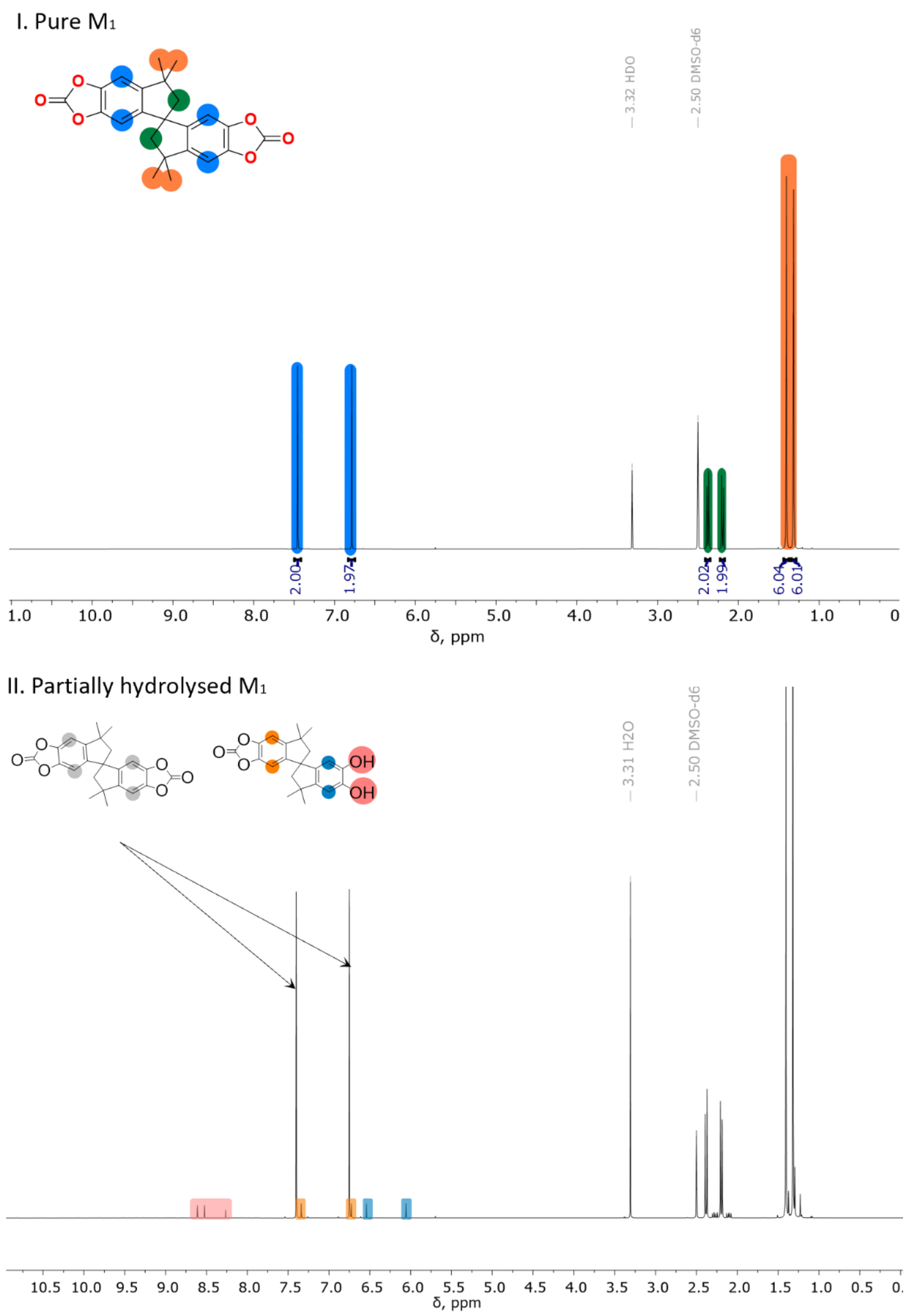

Figure S55. Overlay of ^{1}H NMR traces for pure **M_1** and hydrolysed **M_1** (0.8 mL DMSO-d_6, 0.05 mL H_2O, 50°C, 12 h).

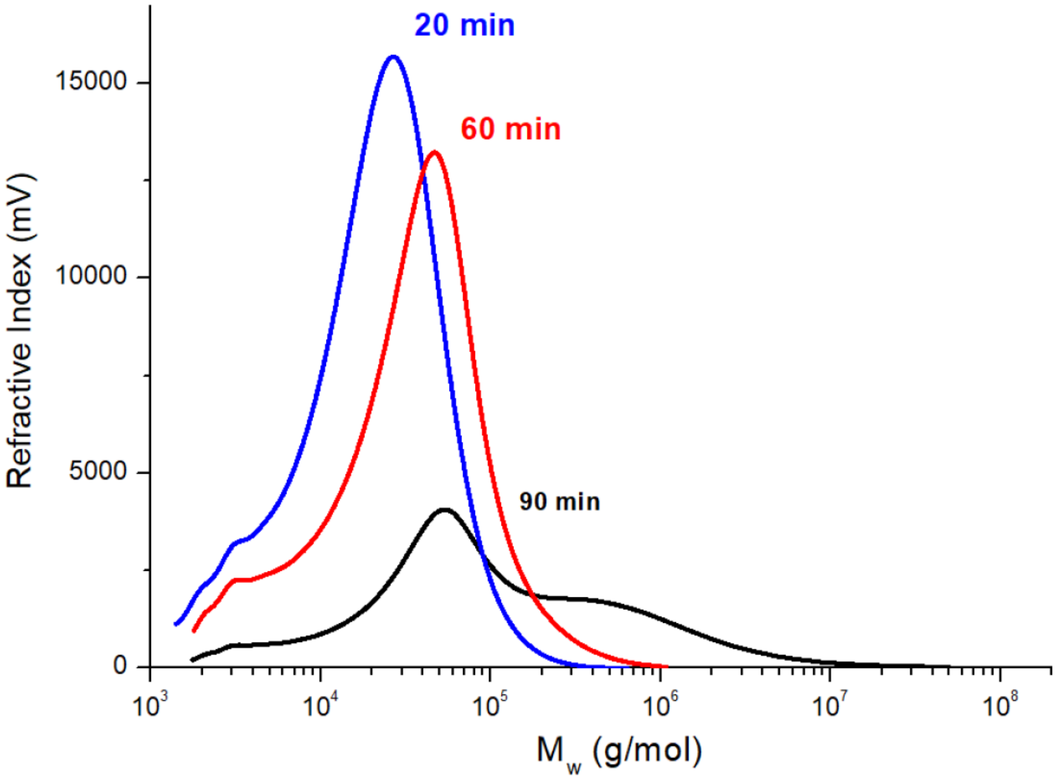

Figure S56. GPC traces of **PHU_1** in 0.1 M $Li(CF_3SO_2)_2N$ (LiTFSI) solution in DMF at 50°C ($DMSO_{anh}$, [M]=0.25 mmol/ml, 50°C).

XIII. References

(1) Fulmer, G. R.; Miller, A. J. M.; Sherden, N. H.; Gottlieb, H. E.; Nudelman, A.; Stoltz, B. M.; Bercaw, J. E.; Goldberg, K. I. NMR Chemical Shifts of Trace Impurities: Common Laboratory Solvents, Organics, and Gases in Deuterated Solvents Relevant to the Organometallic Chemist. *Organometallics* **2010**, *29* (9), 2176–2179. https://doi.org/10.1021/om100106e.

(2) Huggins, M. L. The Viscosity of Dilute Solutions of Long-Chain Molecules. IV. Dependence on Concentration. *J. Am. Chem. Soc.* **1942**, *64* (11), 2716–2718. https://doi.org/10.1021/ja01263a056.

(3) Schulz, G. V.; Blaschke, F. Eine Gleichung Zur Berechnung Der Viscositätszahl Für Sehr Kleine Konzentrationen, [Molekulargewichtsbestimmungen an Makromolekularen Stoffen, IX]. *J. Prakt. Chem.* **1941**, *158* (1–8), 130–135. https://doi.org/10.1002/prac.19411580112.

(4) Ihata, O.; Kayaki, Y.; Ikariya, T. Synthesis of Thermoresponsive Polyurethane from 2-Methylaziridine and Supercritical Carbon Dioxide. *Angew. Chem. Int. Ed.* **2004**, *43* (6), 717–719. https://doi.org/10.1002/anie.200352215.

(5) Ihata, O.; Kayaki, Y.; Ikariya, T. Aliphatic Poly(Urethane−amine)s Synthesized by Copolymerization of Aziridines and Supercritical Carbon Dioxide. *Macromolecules* **2005**, *38* (15), 6429–6434. https://doi.org/10.1021/ma050549o.

(6) Shen, Z.; Zhang, J.; Zhu, W.; Zheng, L.; Li, C.; Xiao, Y.; Liu, J.; Wu, S.; Zhang, B. A Solvent-Free Route to Non-Isocyanate Poly(Carbonate Urethane) with High Molecular Weight and Competitive Mechanical Properties. *Eur. Polym. J.* **2018**, *107*, 258–266. https://doi.org/10.1016/j.eurpolymj.2018.08.006.

(7) Prömpers, G.; Keul, H.; Höcker, H. Polyurethanes with Pendant Hydroxy Groups: Polycondensation of D-Mannitol-1,2:5,6-Dicarbonate with Diamines. *Des. Monomers Polym.* **2005**, *8* (6), 547–569. https://doi.org/10.1163/156855505774597830.

(8) Annunziata, L.; Diallo, A. K.; Fouquay, S.; Michaud, G.; Simon, F.; Brusson, J.-M.; Carpentier, J.-F.; Guillaume, S. M. α,ω-Di(Glycerol Carbonate) Telechelic Polyesters and Polyolefins as Precursors to Polyhydroxyurethanes: An Isocyanate-Free Approach. *Green Chem.* **2014**, *16* (4), 1947–1956. https://doi.org/10.1039/C3GC41821A.
(9) Lambeth, R. H.; Henderson, T. J. Organocatalytic Synthesis of (Poly)Hydroxyurethanes from Cyclic Carbonates and Amines. *Polymer* **2013**, *54* (21), 5568–5573. https://doi.org/10.1016/j.polymer.2013.08.053.
(10) Deng, Y.; Li, S.; Zhao, J.; Zhang, Z.; Zhang, J.; Yang, W. Crystallizable and Tough Aliphatic Thermoplastic Poly(Ether Urethane)s Synthesized through a Non-Isocyanate Route. *RSC Adv.* **2014**, *4* (82), 43406–43414. https://doi.org/10.1039/C4RA05880A.
(11) Li, C.; Li, S.; Zhao, J.; Zhang, Z.; Zhang, J.; Yang, W. Synthesis and Characterization of Aliphatic Poly(Amide Urethane)s Having Different Nylon 6 Segments through Non-Isocyanate Route. *J. Polym. Res.* **2014**, *21* (7), 498. https://doi.org/10.1007/s10965-014-0498-0.
(12) Ochiai, B.; Inoue, S.; Endo, T. Salt Effect on Polyaddition of Bifunctional Cyclic Carbonate and Diamine. *J. Polym. Sci. A Polym. Chem.* **2005**, *43* (24), 6282–6286. https://doi.org/10.1002/pola.21081.
(13) Tomita, H.; Sanda, F.; Endo, T. Polyaddition of Bis(Seven-membered Cyclic Carbonate) with Diamines: A Novel and Efficient Synthetic Method for Polyhydroxyurethanes. *J. Polym. Sci. A Polym. Chem.* **2001**, *39* (23), 4091–4100. https://doi.org/10.1002/pola.10058.
(14) Neffgen, S.; Keul, H.; Höcker, H. Cationic Ring-Opening Polymerization of Trimethylene Urethane: A Mechanistic Study. *Macromolecules* **1997**, *30* (5), 1289–1297. https://doi.org/10.1021/ma9610774.
(15) Schmidt, S.; Gatti, F. J.; Luitz, M.; Ritter, B. S.; Bruchmann, B.; Mülhaupt, R. Erythritol Dicarbonate as Intermediate for Solvent- and Isocyanate-Free Tailoring of Bio-Based Polyhydroxyurethane Thermoplastics and Thermoplastic Elastomers. *Macromolecules* **2017**, *50* (6), 2296–2303. https://doi.org/10.1021/acs.macromol.6b02787.
(16) Sheng, X.; Ren, G.; Qin, Y.; Chen, X.; Wang, X.; Wang, F. Quantitative Synthesis of Bis(Cyclic Carbonate)s by Iron Catalyst for Non-Isocyanate Polyurethane Synthesis. *Green Chem.* **2015**, *17* (1), 373–379. https://doi.org/10.1039/C4GC01294A.
(17) Kihara, N.; Endo, T. Synthesis and Properties of Poly(Hydroxyurethane)s. *J. Polym. Sci. A Polym. Chem.* **1993**, *31* (11), 2765–2773. https://doi.org/10.1002/pola.1993.080311113.
(18) Hosokawa, S.; Nagao, A.; Hashimoto, Y.; Matsune, A.; Okazoe, T.; Suzuki, C.; Wada, H.; Kakiuchi, T.; Tsuda, A. Non-Isocyanate Polyurethane Synthesis by Polycondensation of Alkylene and Arylene Bis(Fluoroalkyl) Bis(Carbonate)s with Diamines. *Bull. Chem. Soc. Jpn.* **2023**, *96* (7), 663–670. https://doi.org/10.1246/bcsj.20230066.
(19) Tomita, H.; Sanda, F.; Endo, T. Polyaddition Behavior of Bis(Five- and Six-Membered Cyclic Carbonate)s with Diamine. *J. Polym. Sci. A Polym. Chem.* **2001**, *39* (6), 860–867. https://doi.org/10.1002/1099-0518(20010315)39:6%253C860::AID-POLA1059%253E3.0.CO;2-2.
(20) Gennen, S.; Grignard, B.; Tassaing, T.; Jérôme, C.; Detrembleur, C. CO_2 -Sourced α-Alkylidene Cyclic Carbonates: A Step Forward in the Quest for Functional Regioregular Poly(Urethane)s and Poly(Carbonate)s. *Angew. Chem. Int. Ed.* **2017**, *56* (35), 10394–10398. https://doi.org/10.1002/anie.201704467.
(21) Unverferth, M.; Kreye, O.; Prohammer, A.; Meier, M. A. R. Renewable Non-Isocyanate Based Thermoplastic Polyurethanes via Polycondensation of Dimethyl Carbamate Monomers with Diols. *Macromol. Rapid Commun.* **2013**, *34* (19), 1569–1574. https://doi.org/10.1002/marc.201300503.
(22) Kihara, N.; Kushida, Y.; Endo, T. Optically Active Poly(Hydroxyurethane)s Derived from Cyclic Carbonate andL-Lysine Derivatives. *J. Polym. Sci. A Polym. Chem.* **1996**, *34* (11), 2173–2179. https://doi.org/10.1002/(SICI)1099-0518(199608)34:11%253C2173::AID-POLA10%253E3.0.CO;2-C.
(23) Besse, V.; Foyer, G.; Auvergne, R.; Caillol, S.; Boutevin, B. Access to Nonisocyanate Poly(Thio)Urethanes: A Comparative Study. *J. Polym. Sci. Part A: Polym. Chem.* **2013**, *51* (15), 3284–3296. https://doi.org/10.1002/pola.26722.
(24) Fidalgo, D. M.; Kolender, A. A.; Varela, O. Stereoregular Poly- *O* -methyl [*m,n*]-polyurethanes Derived from D -mannitol. *J. Polym. Sci. A Polym. Chem.* **2013**, *51* (2), 463–470. https://doi.org/10.1002/pola.26406.
(25) Sardon, H.; Engler, A. C.; Chan, J. M. W.; Coady, D. J.; O'Brien, J. M.; Mecerreyes, D.; Yang, Y. Y.; Hedrick, J. L. Homogeneous Isocyanate- and Catalyst-Free Synthesis of Polyurethanes in Aqueous Media. *Green Chem.* **2013**, *15* (5), 1121. https://doi.org/10.1039/c3gc40319j.
(26) Duval, C.; Kébir, N.; Charvet, A.; Martin, A.; Burel, F. Synthesis and Properties of Renewable Nonisocyanate Polyurethanes (NIPU s) from Dimethylcarbonate. *J. Polym. Sci. Part A: Polym. Chem.* **2015**, *53* (11), 1351–1359. https://doi.org/10.1002/pola.27568.

(27) Ubaghs, L.; Fricke, N.; Keul, H.; Höcker, H. Polyurethanes with Pendant Hydroxyl Groups: Synthesis and Characterization. *Macromol. Rapid Commun.* **2004**, *25* (3), 517–521. https://doi.org/10.1002/marc.200300064.
(28) Tomita, H.; Sanda, F.; Endo, T. Self-Polyaddition of Six-Membered Cyclic Carbonate Having Fmoc-Protected Amino Group: Novel Synthetic Method of Polyhydroxyurethane. *Macromolecules* **2001**, *34* (22), 7601–7607. https://doi.org/10.1021/ma010622k.
(29) More, A. S.; Gadenne, B.; Alfos, C.; Cramail, H. AB Type Polyaddition Route to Thermoplastic Polyurethanes from Fatty Acid Derivatives. *Polym. Chem.* **2012**, *3* (6), 1594. https://doi.org/10.1039/c2py20123b.
(30) Sharma, B.; Keul, H.; Höcker, H.; Loontjens, T.; Benthem, R. V. Synthesis and Characterization of Alternating Poly(Amide Urethane)s from ε-Caprolactone, Diamines and Diphenyl Carbonate. *Polymer* **2005**, *46* (6), 1775–1783. https://doi.org/10.1016/j.polymer.2004.11.024.
(31) Deepa, P.; Jayakannan, M. Polyurethane–Oligo(Phenylenevinylene) Random Copolymers: π-Conjugated Pores, Vesicles, and Nanospheres via Solvent-induced Self-organization. *J. Polym. Sci. A Polym. Chem.* **2008**, *46* (17), 5897–5915. https://doi.org/10.1002/pola.22907.
(32) Zhang, D.; Zhang, Y.; Fan, Y.; Rager, M.-N.; Guérineau, V.; Bouteiller, L.; Li, M.-H.; Thomas, C. M. Polymerization of Cyclic Carbamates: A Practical Route to Aliphatic Polyurethanes. *Macromolecules* **2019**, *52* (7), 2719–2724. https://doi.org/10.1021/acs.macromol.9b00436.
(33) Palaskar, D. V.; Boyer, A.; Cloutet, E.; Alfos, C.; Cramail, H. Synthesis of Biobased Polyurethane from Oleic and Ricinoleic Acids as the Renewable Resources via the AB-Type Self-Condensation Approach. *Biomacromolecules* **2010**, *11* (5), 1202–1211. https://doi.org/10.1021/bm100233v.
(34) Kuran, W.; Rokicki, A.; Romanowska, D. A New Route for Synthesis of Oligomeric Polyurethanes. Alternating Copolymerization of Carbon Dioxide and Aziridines. *J. Polym. Sci. Polym. Chem. Ed.* **1979**, *17* (7), 2003–2011. https://doi.org/10.1002/pol.1979.170170710.
(35) Charles J. Pouchert. *The Aldrich Library of FT-IR Spectra: FT-IR*, Ed. 2.; Aldrich: Milwaukee, Wis., 1997.
